# Methylomes of human CD4 and CD8 memory T lymphocytes reveal tissue-specific epigenetic signatures for maintenance and recall function

**DOI:** 10.1007/s44466-025-00009-x

**Published:** 2025-10-01

**Authors:** Xiangyi Deng, Weijie Du, Gilles Gasparoni, Abdulrahman Salhab, Karl Nordström, Jinchan Li, Viktoria Wagner, Erping Zhang, Joachim Wachtlin, Juliane Bodo, Simon Reinke, Hong Lei, Carsten Perka, Sebastian Hardt, Thomas Dörner, Christoph Holmer, Mario Tönnies, Torsten Bauer, Hyun-Dong Chang, Julia K. Polansky, Jörn Walter, Pawel Durek, Andreas Radbruch, Jun Dong

**Affiliations:** 1 Department of Cell Biology, Deutsches Rheuma-Forschungszentrum Berlin, Institute of the Leibniz Association, Berlin, Germany; 2 Department of Genetics, University of Saarland, Saarbrücken, Germany; 3 Sankt Gertrauden Krankenhaus, Berlin, Germany; 4 Medizinische Hochschule Brandenburg, Neuruppin, Germany; 5 Plastische Und Ästhetische Chirurgie, Berlin, Germany; 6 Berlin-Brandenburg Center for Regenerative Therapies , Charité - Universitätsmedizin Berlin, Corporate Member of Freie Universität Berlin, Humboldt-Universität Zu Berlin and Berlin Institute of Health, Berlin, Germany; 7 Shaanxi Institute for Pediatric Diseases, Xi’an Children’s Hospital, Affiliated Children’s Hospital of Xi’an Jiaotong University, Xi’an, China; 8 Center for Musculoskeletal Surgery, Charité - Universitätsmedizin Berlin, Corporate Member of Freie Universität Berlin, Humboldt-Universität Zu Berlin and Berlin Institute of Health, Berlin, Germany; 9 Department of Rheumatology and Clinical Immunology, Charité - Universitätsmedizin Berlin, Corporate Member of Freie Universität Berlin, Humboldt-Universität Zu Berlin and Berlin Institute of Health, Berlin, Germany; 10 Department of General, Visceral and Vascular Surgery, Charité - Universitätsmedizin Berlin, Corporate Member of Freie Universität Berlin, Humboldt-Universität zu Berlin and Berlin Institute of Health, Berlin, Germany; 11 Respiratory Diseases Clinic Heckeshorn, Helios Klinikum Emil Von Behring GmbH, Berlin, Germany; 12 Schwiete Laboratory for Microbiota and Inflammation, Deutsches Rheuma-Forschungszentrum Berlin, Institute of the Leibniz Association, Berlin, Germany; 13 Institute for Biotechnology, Technische Universität Berlin, Berlin, Germany; 14 Berlin-Brandenburg Center for Regenerative Therapies, Charité - Universitätsmedizin Berlin, Corporate Member of Freie Universität Berlin, Humboldt-Universität zu Berlin and Berlin Institute of Health; Department of Immuno-Epigenetics, Deutsches Rheuma-Forschungszentrum Berlin, Institute of the Leibniz Association, Berlin, Germany; 15 Genomics Data Science Core, Integrated Genomics Services, Sidra Medicine, Doha, Qatar; 16 Department of Neurology and Neurological Sciences, Wu Tsai Neurosciences Institute, Stanford University School of Medicine, Stanford, CA USA; 17 Caritas-Klinik Dominikus Berlin, Allgemein- Und Viszeralchirurgie, Berlin, Germany

**Keywords:** Epigenetic memory, Tissue-resident memory T cells, human tissues, Maintenance, Chemokine receptors, Integrins, Transcription factors, DNA methylation

## Abstract

Adaptive immunity relies on antibodies and memory B and T cells, with memory T cells providing "reactive memory". These cells either circulate in the blood or remain as tissue-resident memory T cells, yet the epigenetic mechanisms underlying their recall function and maintenance are not well understood. Here, we present a comprehensive analysis of 56 reduced representation bisulfite sequencing (RRBS) datasets from 22 memory CD4 and CD8 T-cell populations isolated from human bone marrow, intestine, spleen, lung, skin, and peripheral blood, including surface CD69-positive and CD69-negative cells. Our study reveals unique DNA hypomethylation patterns in tissue-resident memory T cells, particularly in regions associated with genes involved in tissue homing, residency, and transcription factors regulating recall effector memory. The methylomes and differential methylation signatures identified here serve as a valuable resource for understanding the epigenetic program of memory T lymphocytes, their roles in immunological recall, and their maintenance within specific tissues.

**Graphical Abstract:**

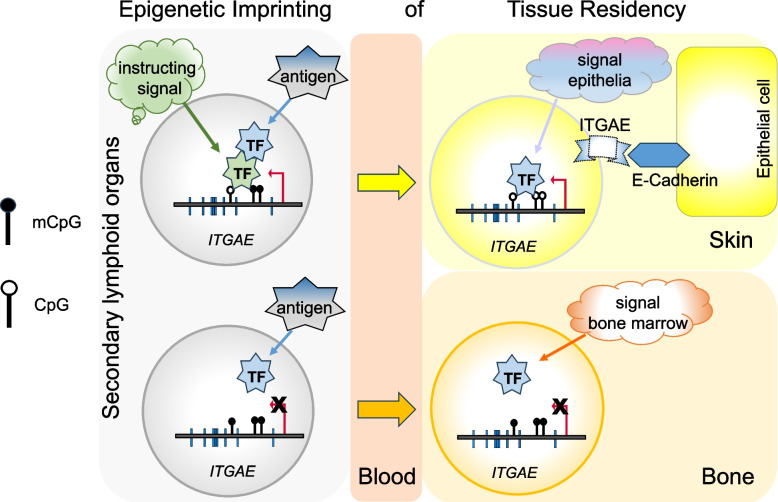

**Supplementary Information:**

The online version contains supplementary material available at 10.1007/s44466-025-00009-x.

## Introduction

Memory (CD4 and CD8) T lymphocytes (Tm) play an important role in protecting the host against recurrent pathogens. While some Tm are circulating through the body, others reside and persist in distinct tissues, such as the bone marrow [1–3], skin [4], intestine [5], lung [6, 7], and spleen [8, 9]. In particular, Tm expressing CD69 on the cell surface are regarded as tissue-resident memory T cells (Trm) [10–12]. CD69^+^ Trm of different tissues share signatures of gene expression [8, 9, 13], but they also differ in expression of other genes, in particular genes encoding chemokine receptors and adhesion molecules [14]. While Trm of epithelial barrier tissues are suggested to provide enhanced local immunity [6, 15], we have shown earlier that Trm in the bone marrow provide polyfunctional long-term memory for systemic challenges, particularly against viruses such as measles, mumps, rubella, and SARS-COV-2 [2, 16]. Bone marrow Trm not only protect the bone marrow itself [17] but also are mobilized into circulation, contributing significantly to systemic secondary immune reactions [18]. Recently, distinct transcriptional changes in chromatin accessibility and functional adaptations have been described for murine CD8^+^ Trm from various tissues in response to viral infection, underscoring the diversity and tissue-specificity of Trm [19, 20].

 Imprinting of Tm genes for recall expression in secondary challenges has been shown to correlate to demethylation of regulatory regions of those genes. We have shown this earlier for the genes encoding interferon-gamma (IFN-γ), interleukin-4 (IL-4), and IL-17A in Th1, Th2, and Th17 cells, respectively [21–25].

In the present study, we have extended upon the German Epigenome Programme (DEEP) and our prior work, which focused on Tm circulating through the blood [26]. Here, we determine the global DNA methylomes of 22 human CD4 and CD8 Tm populations (surface CD69^+^ and CD69^−^) directly isolated from viable bone marrow, intestine, spleen, lung, skin, and peripheral blood. This atlas provides a unique dataset representing the epigenetic profile of human Tm. Our analysis of global epigenetic patterns, as reflected by differentially methylated regions (DMRs), reveal tissue-specific imprinting signatures. These signatures distinguish Tm from skin and lung, relate those from intestine to spleen, and indicate similarities between Tm from spleen and those in blood. Notably, Tm from bone marrow exhibit a distinct DMR signature. In particular, DMRs associated with gene loci encoding chemokine receptor and integrin genes demonstrate tissue-specific imprinting of those genes. Beyond offering a molecular atlas of the epigenetic profile of human Tm, the identified signatures will be instrumental for fate-mapping of tissue-resident Tm, enabling insights into their roles in secondary immune responses and contributions to immunopathology.

## Results

### A methylome atlas of 22 human Tm populations across six somatic tissues

Human Tm were isolated from surgical operation-derived tissue samples of bone marrow, intestine, spleen, lung, and skin, both with and without paired peripheral blood samples, obtained from adult female individuals (18–79 years) without apparent inflammatory immune reactions (Table S1). Among CD4 and CD8 Tm in intestine, lung, and skin, bone marrow, and spleen, Tm expressing surface CD69 range from 20 to 90%, while less than 1% of CD4 and CD8 Tm in blood express CD69 (Fig. S1a and S1b), consistent with previous observations [2]. Except for CD137 and CD38, which were not analyzed in certain tissue groups due to sample limitations, both CD69^+^ (conventional Trm) and CD69^−^ Tm from all analyzed tissues showed no expression of proliferation and activation markers, such as Ki-67, CD25, CD154, HLA-DR, and CD38 (Fig. S1c). CD69^+^ Tm were isolated as bona fide Trm populations, as CD69 expression alone defines tissue residency irrespective of CCR7 status [27].

**Fig. 1:**
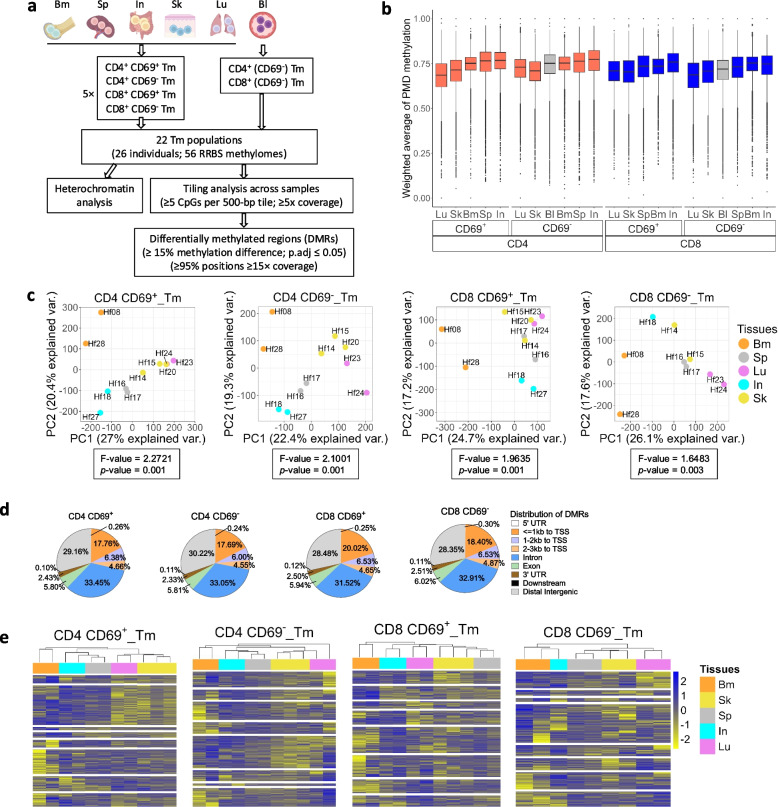
Experimental setup and display of tissue- and lineage-specific methylomes of CD69^+^ and CD69^−^ in CD4 and CD8 Tm populations. **a** Schematic representation illustrating six human somatic tissue types encompassing 22 Tm populations and the corresponding methylome analysis pipeline. **b** Weighted average DNA methylation in CD69^+^ and CD69^−^ CD4 and CD8 Tm from tissues and blood across previously reported partially methylated domains (PMDs) defined by CD4 [26] and CD8 [30] Tem based on the whole genome bisulfite sequencing (WGBS) data. **c** PCA plots of CD69^+^ and CD69^−^ CD4 and CD8 tissue Tm populations, with blood counterparts (> 98% CD69^−^) projected for comparison. Each PCA was generated from DMRs derived exclusively from tissue Tm pairwise comparisons: CD4 CD69^+^ (*n*_DMRs_ = 83,016), CD4 CD69^−^ (*n*_DMRs_ = 70,912), CD8 CD69^+^ (*n*_DMRs_ = 77,715), and CD8 CD69^−^ (*n*_DMRs_ = 103,038). MANOVA results (F- and *P*-values) for tissue groups are shown on each plot. **d** Composition (frequencies) of DMRs (as in C) across various genomic features. **e** Unsupervised hierarchical clustering DMR values across CD69^+^ and CD69^−^ CD4 and CD8 Tm populations in tissues (as in c, but including only ± 3 kb to TSS and intronic DMRs): CD4 CD69^+^ (*n*_DMRs_ = 55,873), CD4 CD69^−^ (*n*_DMRs_ = 46,859), CD8 CD69^+^ (*n*_DMRs_ = 52,987), and CD8 CD69^−^ (*n*_DMRs_ = 65,536). Each row represents a DMR and each column represents a sample. Colors indicate relative DNA methylation levels (Z-score scaled across samples). White lines separate clusters of DMRs identified by hierarchical clustering. Bm, bone marrow; In, intestine; Sp, spleen; Lu, lung; Sk, skin. In **a**, the schematic representation of tissue types were created using BioRender.com

A total of 20 tissue-derived and 2 blood-derived Tm, CD69^+^ and CD69^−^, CD4 and CD8 (Fig. S2a and S2b), were isolated from 26 individuals, including 4 Tm populations which had been partly described previously [18, 26] (Table S1). Methylome data of CD69^+^ and CD69^−^, CD4 and CD8 Tm were generated by reduced representation bisulfite sequencing (RRBS) (*n* = 56) (Fig. 1a) for genome-wide analysis of methylation at the single nucleotide level [28].

Of the 56 methylomes, 22 were obtained from Tm isolated from individual donors, while 34 were generated from pooled samples, 3 donors per pool. In total, 2–3 replicate datasets were obtained for each tissue-derived CD69^+^ and CD69^−^ CD4/CD8 Tm population, with 7 replicate datasets for blood CD4/CD8 CD69^−^ Tm. For intestine, we had one pooled sample for CD8 CD69^−^ Tm. A total of 3.83 billion reads were mapped onto the genome (Table S1), covering approximately 40% of all the CpGs across the entire genomes of the 22 tissue and blood Tm.

The methylome data have been deposited to the European Genome-Phenome Archive (https://ega-archive.org) under the accession numbers EGAS00001001624 [26], EGAS00001005475 [18], and EGAS50000000085.

### Global demethylation of heterochromatin

We first compared the overall extent of genome-wide CpG methylation profiles of long-range partially methylated domains (PMDs) [29] of our various Tm populations. These regions, which span up to hundreds of kilo-base pairs and can encompass up to 75% of the genome, are transcriptionally inactive [30] and associated with late replication timing [31, 32]. Progressive demethylation of PMDs has been associated with extended cellular proliferation [26].

Methylation of PMDs is shown for CD4 and CD8 Tm in Fig. 1b, comparing CD4 Tm populations from tissues to PMD levels of blood CD4 Tm, as published before [26]. For CD8 Tm, we used the baseline PMDs of blood CD8 Tm [30]. The average methylation levels of PMDs for both CD69^+^ and CD69^−^ CD4 Tm from the lung and skin are slightly lower than from bone marrow and blood, while methylation levels for CD4 Tm of from intestine and spleen are slightly higher than from bone marrow and blood. For CD8 Tm, PMD methylation levels are overall low, with slight differences among the various tissues, which is similar to the patterns observed for CD4 Tm. In summary, the analysis of methylation of heterochromatin did not reveal significant differences between Tm from different tissues.

### Tissue- and lineage-specific Tm methylome signatures

To compare methylation across shorter DNA regions, we identified DMRs of 500 base pairs in length, containing at least 5 CpGs, with at least 15% of them differentially methylated between two or more of each CD69^+^ and CD69^−^ populations from both CD4 and CD8 Tm (Fig. 1a). For each donor within each subset we calculated the variance of methylation across compiled tissue-specific DMRs from all comparisons: 50 out of 56 samples (89%) lay within a narrow range of 0.044 and 0.06 (maximum 0.07), corresponding to coefficients of variation of 4–10%, and thus demonstrate highly similar methylation stability between donors (Fig. S3c). To discriminate between Tm tissue-specific and donor-specific differences, we performed principal component analyses (PCAs), based on the compiled tissue-specific DMRs derived from pairwise comparisons of 4 populations of Tm analyzed (Fig. S3a and S3b; Table S2). The resulting tissue-to-donor variance ratios (F-value) range from 1.65 to 2.27, indicating that the variation between tissues is roughly twice that between donors, and identifying DMRs contribute to the tissue-specific variation. The corresponding *p*-values range from 0.003 to 0.001 (Fig. 1c), indicating the high significance of the differences. Moreover, significance remain the same for pooled donor samples and individual donors.

In the PCA, Tm from different tissues form distinct clusters, with bone marrow significantly differing from intestine, spleen, lung, and skin (Fig. 1c). Tissue-specific separation is primarily captured by first principal component (PC1), while second principal component (PC2) primarily reflects donor-specific differences. For CD69^−^ CD4 Tm, the differences are less pronounced compared to their CD69^+^ counterparts (F-value: 2.1, p-value: 0.001 versus F-value: 2.27, p-value: 0.001). The separation of CD8 Tm along PC1 between bone marrow and other tissues is more pronounced than that of CD4 Tm (Fig. 1c). Notably, for CD69^−^ Tm populations, Tm cluster from blood is close to those from spleen (Fig. S3d).

The compiled tissue-specific DMRs of Tm from this analysis represent 3–6% of the entire genome. About 70% of these DMRs cover DNA regions with at least one of five annotated genomic features: 5’ untranslated region (UTR), promoter, exon, intron, or 3’ UTR (Fig. 1d). Notably, over 60% of the DMRs are located within 3 kb upstream or downstream of a transcriptional start site (TSS) or within gene introns, in line with a putative role in regulating the expression of the associated gene.

Hierarchical clustering according to these 70% DMRs reveals distinct tissue-specific epigenetic signatures for Tm (Fig. 1e). Tm from the same tissue of different donors cluster together, confirming the tissue-specificity of the DMR signatures, with only few exceptions, in particular CD4 Tm from intestine (CD69^+^ and CD69^−^) and lung (CD69^−^), and CD8 Tm from skin (CD69^+^), as well as bone marrow (CD69^−^). Tm from skin and lung are closely related, as are those from intestine and spleen. Tm from bone marrow show an exclusive epigenetic signature. Quantitatively, this is reflected by the number of DMRs in pairwise comparisons of CD4 and CD8 Tm from different tissues (Fig. S3a and S3b). Tm signatures from blood are similar to those from spleen, but in general no significant demethylation of any DMR that discriminate between Tm of the various tissues is shown (Fig. S4a and S4b). In summary, tissue-specific DMR signatures are evident from pairwise comparisons, PCA, and hierarchical clustering.

### Tissue-specific imprinting of recall function genes

**Fig. 2:**
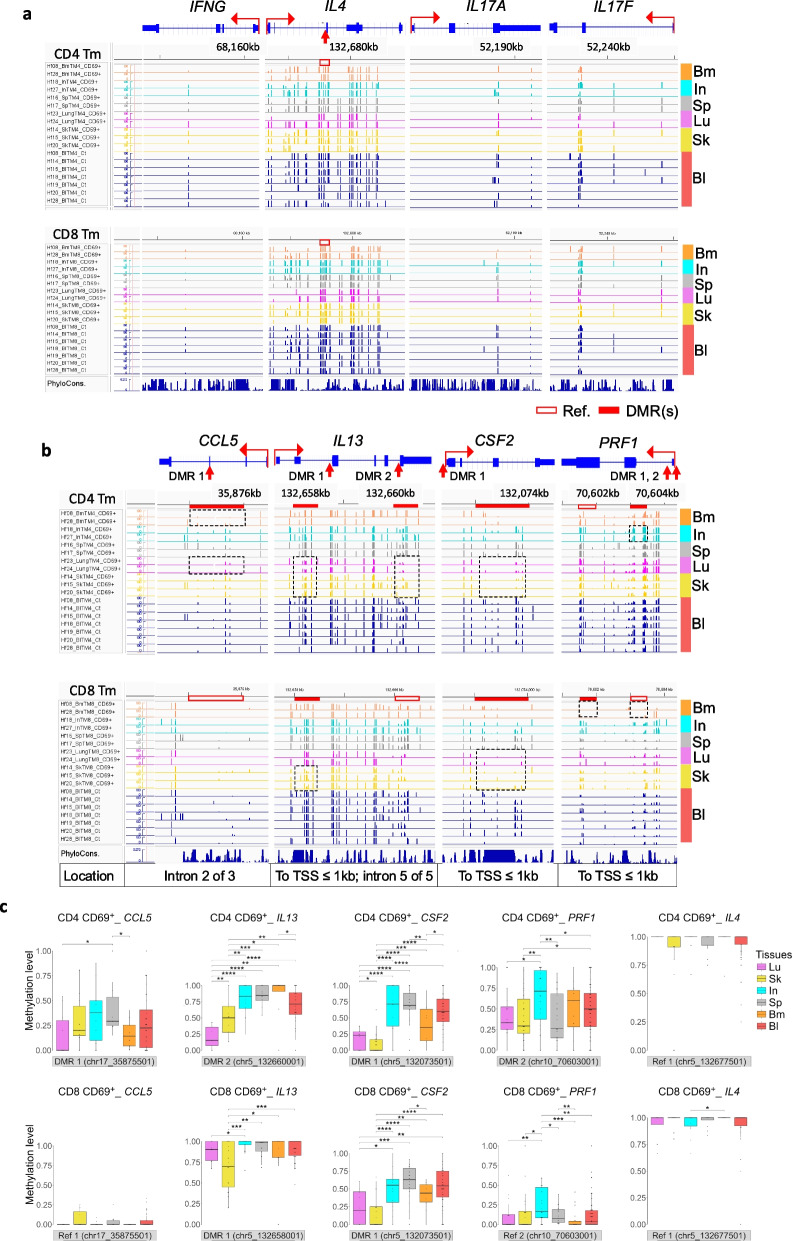
Epigenetic memory of non-expressed effector function genes under steady-state conditions. **a** and **b** IGV visualization of non-significant (**a**) and significant (**b**) tissue-specific hypo- or hyper-DMRs in CD4 and CD8 Tm, including tissue CD69^+^ and blood samples. Differential methylation regions are highlighted by red filled bars, and corresponding comparison tiles are labeled as "Ref", as shown by open bars for visual reference. Methylation is plotted on a 0–100 scale, representing the percentage of methylated reads at each CpG site (0 = unmethylated, 100 = fully methylated). Loci without CpG sites—due to sequence context or RRBS coverage—appear as gaps. CpG sites within defined DMRs or corresponding tiles showing low methylation (values near 0) indicate demethylation. Multiple DMRs may appear for the same gene, oriented by transcription direction. PhyloCons track shows nucleotide-level conservation across multiple species, with higher bars indicating greater evolutionary conservation. **c** Quantification of DNA methylation levels at the indicated DMRs or corresponding positions (labeled "Ref") in panels a and b. Methylation levels were compared across distinct tissue CD69^+^ Tm populations and blood Tm using rank-based statistics. Box plots display median, interquartile range, and whiskers, with Wilcoxon test significance: ****p < 0.0001, *** *p* < 0.001, ** *p* < 0.01, * *p* < 0.05

For more detailed inspection, we initially focused on DMRs associated with genes encoding recall effector functions as a correlate of functional memory. Genes encoding cytokines and chemokines are not expressed by resting Tm, but imprinted for re-expression upon cognate reactivation of Tm ("Cytokine memory") [22]. Interestingly, we observe no significant difference exceeding 15% in the methylation of 500 bp tiles containing more than 5 CpGs associated with *IFNG*, *IL4*, and *IL17A/F* gene loci of CD4 Tm (Fig. 2a; Table S3). This finding indicates that Th1, Th2, and Th17 lineage cells are present in similar proportions across tissues. However, for gene loci encoding other silent genes of functional memory of Th1, Th2 and Th17 lineage, the various Tm populations show tissue-specific DMRs, associated with genes such as *CCL5* (*RANTES*), *IL13*, and *CSF2* (Fig. S5a and S5b). Differential methylation of these tissue-specific DMRs was quantitated by Integrative Genomics Viewer (IGV) (Fig. 2b). CD69^+^ CD4 Tm from bone marrow and lung show hypomethylation of DMR1 in the second intron of *CCL5* (*RANTES*), a secondary Th1 chemokine [33]. This DMR is essentially demethylated in all CD69^+^ CD8 Tm. DMR1 of the secondary Th2 cytokine *IL13* is selectively demethylated in CD69^+^ CD4 Tm from lung and skin, and CD69^+^ CD8 Tm from skin, and completely methylated in all other CD8 Tm. DMR1 of *CSF2*, encoding GM-CSF, a secondary Th17 cytokine [34], is demethylated selectively in CD69^+^ and CD69^−^ CD4 and CD8 Tm from lung and skin, as well as bone marrow with less degree (Fig. 2c and S5c). Methylation of DMR1 at the promoter and 5’ flank of *PRF1* [35], a region with binding sites for STAT, NF-κB, and AP-1 [36], is significantly higher in CD4 and CD8 CD69^+^ Tm from intestine, as compared to other tissues. Taken together, these results reveal differential imprinting of the recall genes *CCL5*, *IL13*, *CSF2*, and *PRF1* in Tm from lung, skin, and bone marrow.

### Tissue-specific imprinting of navigation, mobility and adhesion genes

**Fig. 3:**
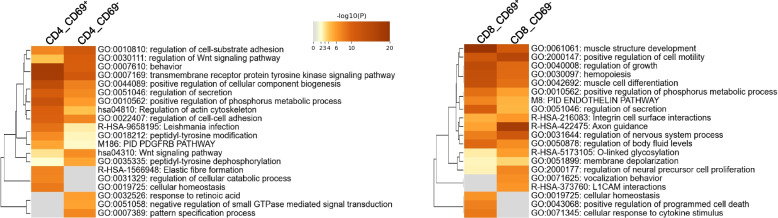
Gene enrichment analysis of tissue-specific methylation patterns in Tm populations. Top 20 enriched pathways and gene ontology (GO) terms for the top 3000 genes derived from the compiled tissue-specific promoter/intron DMRs across all comparisons of the four tissue-specific DMR set: CD69^+^ or CD69^−^ CD4 or CD8 Tm populations, as shown in Fig. 1e. Enriched terms were identified using Metascape, clustered based on Kappa-statistical similarities, and each cluster is represented by the term with the best p-value. Heatmap cells are colored by *p*-values, with white cells indicating no enrichment for that term in the corresponding gene list

We next focused on DMRs annotated in promoters and introns of genes involved in tissue navigation and adhesion in Tm. Gene enrichment analysis (e.g. GO) of the top 3000 genes (Table S4) derived from the compiled tissue-specific DMRs across all comparisons (Fig. 1e), points to genes involved in homing and retention within tissues. CD69^+^ and CD69^−^ CD4 Tm share about half of their associated DMR genes, as well as most of functional pathways annotated (Fig. S6). These include GO:0010810 (regulation of cell-substrate adhesion), GO:0030111 (regulation of Wnt signaling pathway), hsa04810 (regulation of actin cytoskeleton), and GO:0022407 (regulation of cell-cell adhesion) (Fig. 3). Alike, DMR genes annoted to functional pathways of CD69^+^ and CD69^−^ CD8 Tmare relevant to tissue residency and maintenance, such as GO:2000147 (positive regulation of cell motility), GO:0051046 (regulation of secretion), R-HSA-216083 (integrin cell surface interactions), and GO:0031644 (regulation of nervous system process) (Fig. 3 and S6).

Previous studies had claimed transcriptional gene expression signatures of tissue-resident Tm in humans and mice [8, 9, 13]. Our present study reveals that, of 31 human signature genes described in those studies, 25 genes in CD69^+^ CD4 Tm (Fig. S7a; Table S5) and 21 genes in CD69^+^ CD8 Tm (Fig. S7b; Table S5) are not uniformly imprinted in CD69^+^ Tm isolated from the various tissues. Those genes rather show tissue-specific differential methylation, as is evident from the hierarchical clustering according to DMRs associated with 25 genes in CD69^+^ CD4 Tm and 21 genes in CD69+ CD8 Tm (Fig. S7a and S7b). For *PDCD1* encoding PD-1, DMRs 1-4 are hypomethylated in CD69^+^ CD4 Tm from spleen and bone marrow, while Tm from skin and intestine exhibit progressive hypomethylation, mainly in DMRs 2-4. CD69^+^ and CD69^−^ CD4 Tm from lung show methylation or hypermethylation across all DMRs (Fig. S7c). In spleen and intestine, Tm display an inverse correlation between hypo-/hyper-methylation of DMR1 and gene transcription (Fig. S7e). CD69^−^ CD4 Tm from spleen, bone marrow, and skin are significantly demethylated at DMR4, while Tm from intestine show intermediate methylation (Fig. S7c). Additionally, CD69^+^ CD8 Tm are demethylated at DMR3 in skin and bone marrow, while CD69^−^ CD8 Tm from skin show demethylation at DMR4 (Fig. S7d). In summary, tissue CD69^+^ (and to some extent tissue CD69^−^) CD4 and CD8 Tm exhibit significant tissue-specific demethylation of genes identified as signature markers of tissue-resident Tm. This epigenetic profile highlights the remarkable heterogeneity of Tm populations across different tissues.

**Fig. 4:**
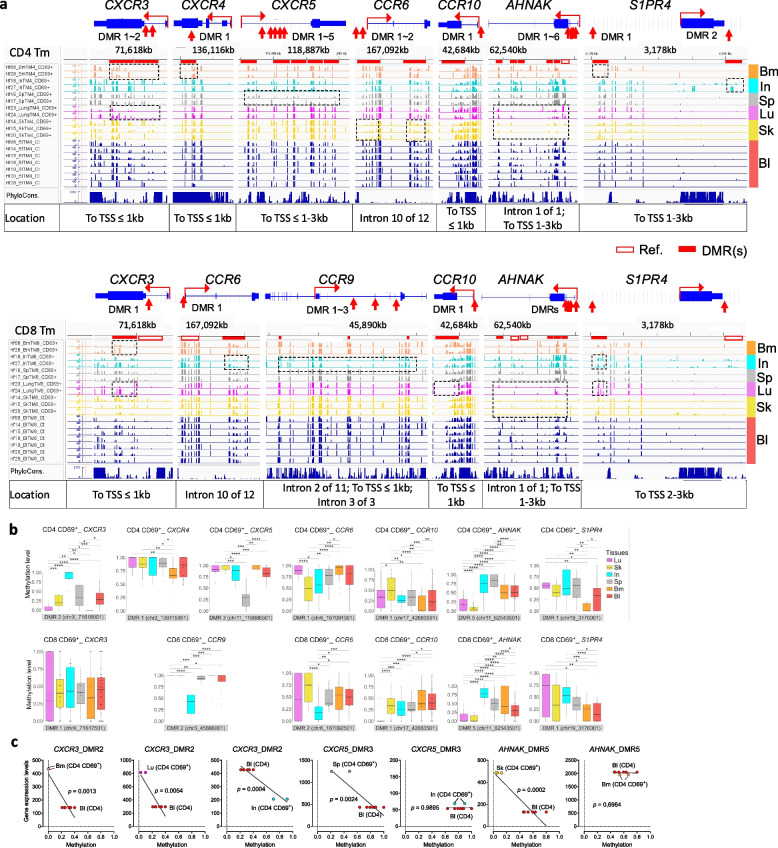
Epigenetic memory of genes involved in homing to tissues. **a** IGV visualization of tissue-specific hypo- or hyper-DMRs for both CD4 and CD8 Tm, including tissue CD69+ and blood samples. **b** Quantification of methylation difference for exemplar DMRs shown in panel a. In **a** and **b**, Key symbols and methodologies are as described in Fig. 2. **c** Correlation analysis between DMRs and their corresponding gene expression levels in the indicated tissue CD69^+^ CD4 Tm and blood cells, using simple linear regression in GraphPad Prism. The line of best fit is shown, with *p*-values indicating significance. Gene expression levels on y-axes were calculated from non-log-transformed values derived from different platforms: linear-scale microarray intensity, DESeq2-normalized counts, or counts per million (CPM), as detailed in the Methods

Attraction to and migration into distinct tissues involve the differential expression of genes encoding chemokine receptors and factors regulating cellular mobility. The DMRs of such gene loci (Table S6) are shown in Fig. S8 for CD69^+^ CD4 and CD8 Tm. Each tissue exhibits a specific methylation profile, with similarities observed between intestine and spleen, as well as lung and skin, while bone marrow displays a distinct pattern. For specific chemokine receptor genes, DMR2 of *CXCR3* is selectively demethylated in CD4 Tm from bone marrow and lung (Fig. 4a, b, and S8b). The imprinting of *CXCR3* in CD4 Tm from bone marrow and lung correlates with gene expression in *ex vivo* cells, as compared to Tm from blood (Fig. 4c). Similarly, hypomethylation is observed for *CXCR5* in CD4 Tm from spleen, correlating with transcriptional activity in *ex vivo* cells (Fig. 4c). Bone marrow CD4 Tm show selective imprinting of *CXCR4* and *S1PR4* gene loci, whereas skin Tm exhibit demethylation of DMRs associated with *CCR6* (Fig. 4a and b). CD8 Tm from intestine display hypomethylated DMRs for *CCR9* and *CCR6* (Fig. 4a and b).

Among genes possibly involved in cellular mobility, we highlight the neuroblast differentiation-associated protein gene *AHNAK*, which shows selective demethylation of associated DMRs in CD69^+^ and CD69^−^ CD4 and CD8 Tm from lung and skin (Fig. 4a, b, S8d, and S8e). The demethylation correlates with transcriptional activity of *AHNAK* gene in CD69^+^ CD4 Tm from skin (Fig. 4c).

In summary, imprinting by demethylation marks the chemokine receptor genes *CXCR3* and *CXCR4* in CD69^+^ CD4 Tm from bone marrow, *CXCR5* in those from spleen, *CCR6* in those from skin, and *CCR9* in CD69^+^ CD8 Tm from intestine. Interestingly, the mobility-related gene *AHNAK* is uniquely imprinted in Tm from skin and lung.

Further, in the context of navigation and mobility, hierarchical clustering of DMRs of 21 genes in CD69^+^ CD4, and 22 integrin genes in CD69^+^ CD8 Tm (Table S7) reveals tissue-specific demethylation patterns (Fig. S9a). As with previous data, this analysis relates Tm from lung to those of skin, and Tm from intestine to those of spleen, while bone marrow Tm show distinct patterns unrelated to any of tissue.

**Fig. 5:**
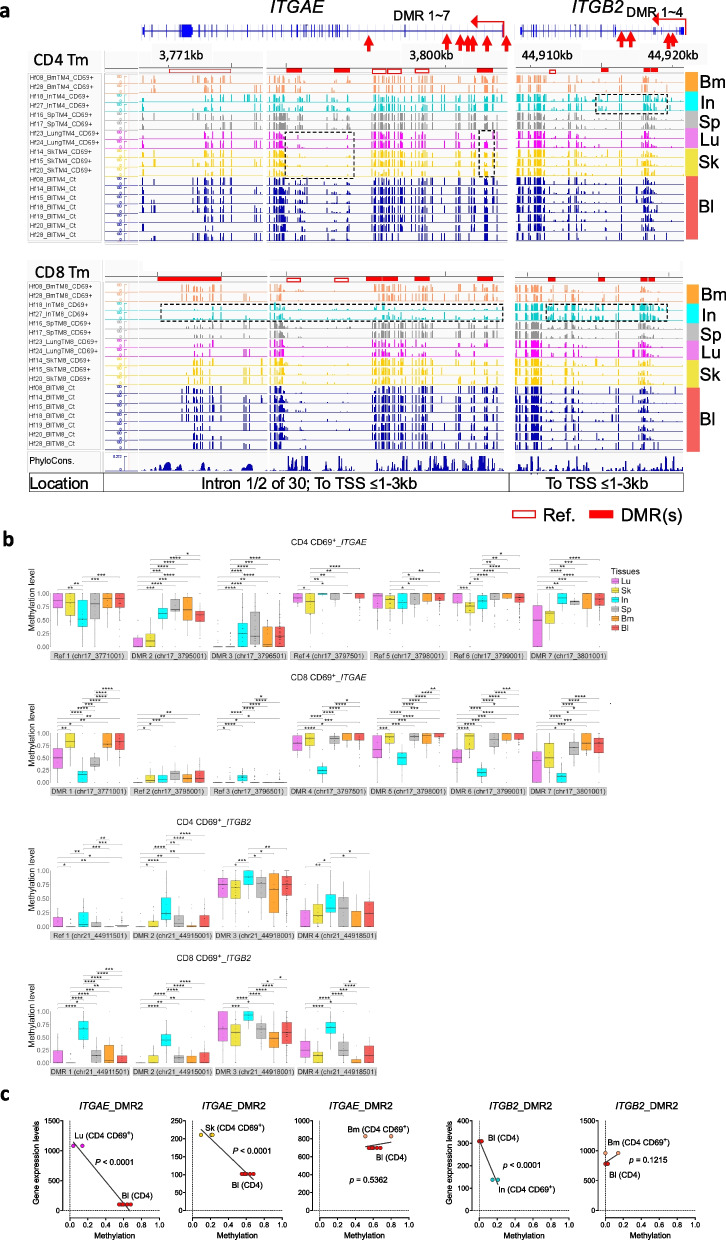
Epigenetic memory of integrins involved in staying in specific tissues. **a** IGV visualization of tissue-specific hypo- or hyper-integrin DMRs for both CD4 and CD8 Tm, including tissue CD69^+^ and blood samples. **b** Quantification of methylation difference for exemplar DMRs shown in panel A. Key symbols and methodologies are as described in Fig. 2. **c** Correlation analysis between DMRs and their corresponding gene expression levels in the indicated tissue CD69^+^ CD4 and blood cells. Key symbols and methodologies are as described in Fig. 4

At higher resolution, we analyzed the methylation of *ITGAE* locus, encoding integrin subunit alpha E, also known as CD103, a ligand of E-cadherin on epithelial cells. CD103 expression has been previously described in CD8^+^ Tm [37] and CD4^+^ Tm [38] residing in epithelial tissues such as skin and lung. Accordingly, CD69^+^ CD4 Tm from lung and skin show significant demethylation of DMR2, DMR3, and DMR7 (Fig. 5a and b). CD69^−^ CD4 Tm from lung and skin also exhibit demethylation of DMR2 (Fig. S9b). The demethylation of DMR2 in *ITGAE* locus correlates with gene transcription in CD69^+^ CD4 Tm from lung and skin in steady-state conditions (Fig. 5c). Interestingly, in CD69^+^ CD8 Tm, DMR1 and DMR4-7 are significantly demethylated in intestine, but not in Tm from skin, blood, and bone marrow (Fig. 5a and b).

*ITGB2* encoded integrin beta 2, also known as CD18, along with CD11a (integrin alpha L) form the leukocyte adhesion molecules (LFA-1) in Tm, which binds to the intercellular adhesion molecule 1 (ICAM1) expressedby stromal cells of bone marrow [1, 3]. All 4 DMRs of *ITGB2* genes are located within introns. In CD4 Tm, DMR1, DMR2, and DMR4 are largely demethylated, while DMR3 remains largely methylated in Tm from all tissues except intestine. Tm from intestine, particularly CD8 Tm, show significantly higher methylation at these loci (Fig. 5a, b, and S9c). The selective methylation of DMRs in intestinal Tm correlates with reduced gene transcription in these cells.

Taken together, imprinting via demethylation of integrin genes is tissue-specific for Tm and correlates with transcriptional activity of the respective genes in steady-state conditions.

### Differential methylation of gene encoding transcription factors

**Fig. 6:**
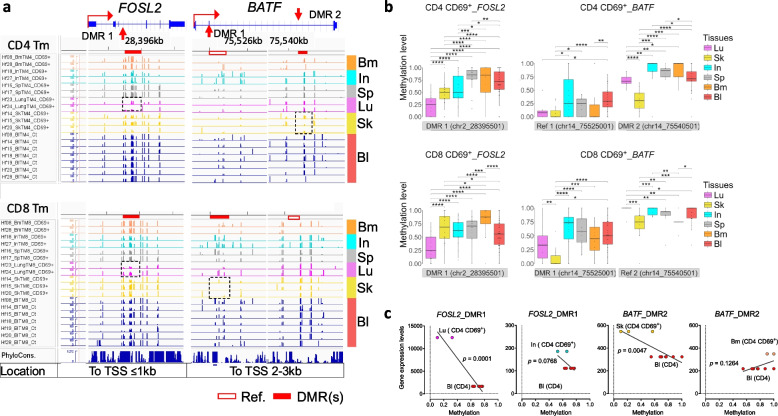
Epigenetic memory of AP-1 family member genes. **a** IGV visualization of tissue-specific hypo- or hyper-AP-1 family member DMRs for both CD4 and CD8 Tm, including tissue CD69^+^ and blood samples. **b** Quantification of methylation difference for exemplar DMRs shown in a. Key symbols and methodologies are as described in Fig. 2. **c** Correlation analysis between DMRs and their corresponding gene expression levels in the indicated tissue CD69^+^ CD4 Tm and blood cells. Key symbols and methodologies are as described in Fig. 4

While epigenetic program via demethylation of recall and maintenance genes likely facilitates their regulation by methylation-sensitive transcription factors (TFs), the expression of methylation-insensitive TFs might instead be governed directly by DNA methylation. In line with this, gene loci encoding TFs differentially expressed by Tm from different tissues, such as AP-1 family members [39] and *RUNX3* [19] are also differentially methylated. DMR1 of *FOSL2* is selectively demethylated in CD69^+^ CD4 and CD8 Tm from lung, and *BATF* is selectively demethylated in CD69^+^ CD4 (DMR2) and CD8 (DMR1) Tm from skin (Fig. 6a and b), correlating with transcription of *FOSL2* gene in CD69^+^ CD4 Tm from lung, and transcription of *BATF* in CD69^+^ CD4 Tm from skin (Fig. 6c). DMR1-5 of *RUNX3* are selectively demethylated in CD69^+^ CD8 Tm from intestine, which is less significant in CD69^+^ CD4 Tm from intestine and lung, as compared to Tm from other tissues (Fig. S10a and S10b).

Tissue-specific demethylation was also observed for DMRs associated with genes encoding zinc finger (ZNF) transcription factors which are involved in memory lineage fate determination [13]. CD4 and CD8 CD69^+^ Tm from various tissues analyzed display differential methylation for 254 and 198 *ZNF* gene loci, respectively, in particular with DMRs ranking among the top 10% differentially methylated in pairwise comparisons (Fig. S11a; Table S8). *ZNF683* shows significant hypomethylation of the associated DMR1 within the highly conserved first intron only in CD69^+^ CD8 Tm from lung (Fig. S11b and S11c). CD69^+^ CD4 Tm from lung and skin display hypomethylation of *ZNF276*, while CD69^+^ CD4 Tm from intestine and spleen are hypomethylated at DMR1 and DMR2 of *ZNF148*, and those Tm from bone marrow and intestine demonstrate hypomethylation of *ZNF814*. While *ZNF28* is demethylated in most Tm, except for CD69^+^ CD8 Tm from lung (Fig. S11b and S11c).

Another family of TFs displaying tissue-specific differential methylation is Krüppel-like Factors (KLF) (Fig. S12a; Table S9). *KLF6* has recently been identified as selectively expressed in skin resident Tm [39]. Accordingly, CD69^+^ and CD69^−^ CD4 Tm and CD69^+^ CD8 Tm from skin show selective demethylation of *KLF6* (Fig. S12b and S12c). Likewise, *KLF13* is expressed by intestinal CD69^+^ CD8 Tm [40], and correspondingly, DMR5 and DMR6 of *KLF13* gene are selectively demethylated in CD69^+^ CD8 Tm. Interestingly, CD69^+^ CD4 Tm from intestine and spleen exhibit selective hypermethylation at DMR1 and DMR2 of *KLF13* (Fig. S12b and S12c). CD69^−^ CD4 and CD8 Tm display a similar pattern to their CD69^+^ counterparts, although less pronounced (Fig. S12b-d).

In summary, tissue-specific demethylation of *AP-1*, *ZNF*, and *KLF* correlates with their tissue-specific expression in tissue-resident Tm.

## Discussion

A hallmark of adaptive immunological memory is the imprinting of memory lymphocytes for immediate recognition and response to recurrent antigenic challenges [22]. The molecular basis of this imprinting involves rewiring of TF networks [42, 43] and epigenetic modifications, including DNA demethylation at regulatory regions of target genes [23–25]. Here, we present a global methylome atlas of 22 distinct populations of human Tm, including CD4 and CD8 Tm from bone marrow, intestine, spleen, lung, skin, and blood, both CD4 and CD8, and of CD69^+^ and CD69^−^ Tm. This dataset provides a comprehensive overview of the epigenetic memory of “recall” genes associated with Th1, Th2, and Th17 Tm, and reveals tissue-specific differences among bulk ex vivo Tm from distinct tissues. Notably, differentially expressed genes involved in homing and persistence are differentially methylated, i.e. Tm are epigenetically imprinted for maintenance within their respective tissues. Tissue-specific methylation signatures are observed in both surface CD69^+^ and CD69^−^ Tm subsets from a given tissue. These findings underscore the extensive compartmentalization of T cell memory, highlight the diversity of tissue-resident Tm populations [44], and in practical terms, provide a means to track tissue-resident Tm when they migrate out of their original tissues [18].

Among 56 methylomes presented (generated from 22 individuals and 34 pooled samples), we observed a high inter-individual variability as the second principal component byPCA. This probably reflects imprinting of memory cells by the different individual immunological challenges and thus the heterogeneous composition of memory T cells in each tissue compartment as such. The prime component of variability reflects differences among Tm isolated from different tissues, i.e. the common denominator of tissue-specific epigenetic program. These tissue-specific epigenetic signatures also reveal relatedness of tissue compartments. Hierarchical clustering relates Tm from lung to those from skin, and Tm from intestine to those from spleen, with an exclusive signature for Tm of bone marrow.

This atlas serves as a foundational resource, providing global methylome landscapes of Tm isolated from these six different human tissues. While earlier studies on the methylomes of circulating Tm emphasized epigenetic modifications in heterochromatin, likely reflecting their proliferating history [26], our comparison of Tm methylomes across tissues reveals extensive differential methylation in euchromatin as well. 70 to 72% of 70,000–100,000 DMRs of Tm are associated with gene bodies (∼9%) or, more commonly, with putative regulatory regions of genes (∼61-63%). In this initial overview of the dataset, we here present examples of epigenetic memory of genes involved in Tm recall and genes that are relevant for and expressed by Tm in steady-state memory within specific tissues.

Regarding the epigenetic memory of signature recall genes in Th1, Th2, and Th17 Tm, including *IFNG*, *IL4*, and *IL17*, we observe no difference between Tm from the analyzed tissues. This suggests that Th1, Th2, and Th17 Tm are equally represented across tissues, and these genes are uniformly imprinted for re-expression. However, when examining the epigenetic memory of secondary genes in Th1, Th2, and Th17 Tm, we note significant differences. For example, DMR2 of the Th2 cytokine gene *IL13* [45] is significantly less methylated in CD69^+^ CD4 Tm from lung and skin, while DMR1 of the Th1 chemokine *CCL5* [46] shows similar hypomethylation patterns in Tm from bone marrow and lung. Interestingly, DMR1 of *CSF2*, a secondary Th1/Th17 cytokine gene [47] encoding GM-CSF, is significantly demethylated in lung and skin in both CD69^+^ CD4 and CD8 Tm, suggesting these tissues contain more Th2 memory cells than other tissues, such as the bone marrow. Additionally, DMRs of *PRF1*, encoding perforin, are significantly demethylated in CD69^+^ CD8 Tm from spleen, bone marrow, lung, skin, and blood, while less demethylated in Tm from intestine. Previous studies have shown that hypomethylation at the DMR regions of *CSF2* and *PFR1* regulate re-expression of GM-CSF and perforin respectively upon T cell receptor (TCR) activation [35, 48] in tissue-resident Tm [49].

Our analysis reveals that core signature genes expressed by the bona fide tissue-resident CD69^+^ Tm are not uniformly marked in CD69^+^ Tm of all tissues. For instance, the signature gene *PDCD1* shows graded demethylation in Tm from spleen and bone marrow (DMR1-4), intestine, skin and blood (DMR2-4), while remains methylated (DMR1-4) in lung Tm. These (differential) methylation patterns align with the inverse correlation between *PDCD1* gene expression (as observed in Tm from spleen) and its proposed role in regulating local immune responses and promoting persistence within these tissue environments [50]. ZNF683, suggested to be critical for Trm residency [13], is demethylated selectively in lung CD69^+^ CD8 Tm.

Navigation of Tm and their precursors into (and out of) distinct tissues is tightly regulated by various factors, including tissue-specific homing signals differentially expressed by Tm and their precursors [51, 52]. Interestingly, while the genes responsible for tissue homing are probably no longer essential in steady-state conditions, they exhibit differential methylation patterns. This suggests that these genes are epigenetically imprinted in Tm, potentially preparing them for future navigation back to their respective tissues. For instance, the pro-inflammatory chemokine receptor gene *CXCR3* is consistently demethylated in CD69^+^ CD4 Tm across most tissues, except those within the anti-inflammatory environment of intestine. Similarly, *CXCR4*, which encodes the protein to attract cells into the bone marrow [53, 54], is demethylated exclusively in CD69^+^ CD4 Tm from bone marrow. As previously shown [1, 3], these Tm from bone marrow are likely individually conjugated to CXCL-12-expressing stromal cells. In contrast, *CXCR5* is demethylated solely in CD69^+^ CD4 Tm from spleen which encodes the membrane receptor responsible for recruiting Tm into germinal centers [55]. These demethylation patterns significantly correlate with the transcriptional activity of their respective genes [52]. Moreover, *AHNAK*, a gene involved in formation of pseudopodia and reorganization of the actin cytoskeleton [56], shows significant demethylation in both CD69^+^ and CD69^−^ CD4 and CD8 Tm from skin and lung. This observation points to a different functional adaptation of Tm in skin and lung compared to bone marrow. In steady-state conditions, Tm in bone marrow adopt a more quiescent phenotype [1, 3, 17], while those in skin are motile, as noted previously [57]. This divergence underscores the tissue-specific lifestyle and functional requirements of Tm.

The trafficking of Tm from the blood into tissues, followed by maintenance in specialized niches, is critically mediated by integrins expressed on Tm [58] and their interaction with ligands on niche-supporting cells [59]. Our findings reveal that these integrin-related genes are also epigenetically imprinted through selective demethylation. For example, *ITGAE*, the gene encoding integrin-alpha E, also known as CD103, is a hallmark of tissue-resident Tm in peripheral tissues, including skin [8, 60]. It has been proposed as a generic signature gene of tissue-resident Tm [8], except for Tm from bone marrow [61], spleen [8], and liver [62]. The ligand for ITGAE is E-cadherin, which is expressed by epithelial cells but not by stromal cells of bone marrow [63]. Correspondingly, *ITGAE* exhibits selective demethylation in CD69^+^ CD4 Tm from skin and lung, as well as CD69^+^ CD8 Tm from intestine, lung, skin, and spleen, but not in Tm from bone marrow. In contrast, *ITGB2* is differentially demethylated in Tm from bone marrow, aligning with its co-expression with integrin alpha-L (CD11a) in these cells [2], which together form the ligand for ICAM expressed stromal cells in bone marrow [64]. CD69^+^ CD8 Tm from intestine express either CD103 or ITGB2 [41], reflecting the heterogeneity within this population. This is consistent with our observation of intermediate levels of demethylation across most of the DMRs associated with both genes in the mixed populations analyzed here.

Epigenetic memory through the demethylation of CpGs is believed to enable the binding of "methylation-sensitive" TFs, which do not bind to methylated DNA although present in a given cell [65]. Consequently, demethylation serves to individualize the transcriptomes of cells expressing TFs. In contrast, the activity of "methylation-insensitive" TFs is regulated primarily by the expression of their respective genes, as we have demonstrated previously for GATA3 [23]. As we show here, such TF genes are subject to epigenetic memory in resting Tm as well. In accordance with the selective expression of the AP-1 monomers *FOSL2* in CD69^+^ Tm from lung, and *BATF* in Tm from skin, both CD4 and CD8 Tm display selective demethylation of these TF genes. AP-1 has been described as a key TF for memory (T cell) maintenance [66–69], with studies in murine models showing upregulation of AP-1 genes in CD69^+^ CD8 Tm from skin and intestine [40]. Here, we further demonstrate that selective demethylation and expression of the AP-1 monomer genes *FOSL2* and *BATF* in Tm, along with the concurrent demethylation and expression of the AP-1-dependent *ITGAE* gene, represents the epigenetic memory of a regulon that governs tissue-specific traits and function of epithelial Tm. Additionally, for intestinal CD69^+^ CD8 Tm, we find selective demethylation of *RUNX3*, which encodes a TF known to regulate *ITGAE* expression [19]. This observation is consistent with prior findings that *RUNX3* hypomethylation enhances expression of *ITGAE* in CD8 T cells [70].

We also report an initial analysis of differential methylation of *KLF* and *ZNF* genes in Tm from various tissues. The observed epigenetic memory correlates well with the reported expression patterns of these TFs, although the precise implications of this differential methylation for tissue-specific maintenance of Tm remain to be elucidated. Epigenetic memory by demethylation highlights the relevance of *KLF6* for CD69^+^ CD8 Tm from skin [40], *ZNF276* for CD69^+^ Tm from skin [71], *KLF13* and *ZNF148* for CD69^+^ Tm from intestine [41, 72]. Our results support a model in which tissue-specific DNA methylation patterns contribute to the functional specialization of Trm by modulating chromatin accessibility and transcription factor binding at key regulatory regions. These epigenetic changes may influence the expression of genes involved in retention, migration, and effector functions, thereby reinforcing tissue residency. This model is consistent with prior work demonstrating close relationships between DNA methylation, chromatin structure, and transcriptional regulation in T cells [19, 73].

Tissue-specific cues such as local cytokines, antigenic exposure, and metabolic context likely drive the establishment of these epigenetic programs, while inter-individual differences may reflect immune history, tissue environment, age, or hormonal status. Although we lack chromatin accessibility data to directly test regulatory activity, significant correlations between DMRs and gene expression—based on publicly available transcriptome datasets—support the functional relevance of our findings. Future studies integrating matched multi-omic profiles (e.g., methylome, ATAC-seq, transcriptome) from both sexes and incorporating functional validation (e.g., CRISPR-mediated methylation editing or gene perturbation) will be essential to further define how tissue-specific DNA methylation programs shape Trm identity and function.

In summary, the methylome atlas presented here for human Tm across various tissues provides a unique global overview of epigenetic memory characteristic of these cells, which play a critical role in maintaining adaptive immunity. This atlas reveals tissue-specific epigenetic signatures, including differential imprinting of the same genes in CD4 and CD8 Tm, even within the same tissue. It also highlights the epigenetic regulation of genes essential for Tm homing to, and residing in a given tissue in the steady state of memory, i.e. in the maintenance phase. These findings offer a valuable foundation for investigating methylation patterns in DNA regions crucial for gene expression and chromatin organization, defining the interaction of regulatory proteins with their target sequences, and advancing the molecular understanding of "imprinting" of immunity and immunopathology. Beyond this, the tissue-specific methylation signatures of resident Tm (Table S10) provide a means to track these cells upon reactivation and migration, even as they undergo significant transcriptomic changes [18].

## Supplementary Information

Supplementary Material 1: Materials and Methods.

Supplementary Material 2: Fig. S1-S4, related to Fig. 1 and Tables S1 and S2. Fig. S5, related to Fig. 2 and Table S3. Fig. S6, related to Fig. 3 and Table S4. Fig. S7, related to Table S5. Fig. S8, related to Fig. 4 and Table S6. Fig. S9, related to Fig. 5 and Table S7. Fig. S11, related to Table S8. Fig. S12, related to Table S9.

Supplementary Material 3: Tables S1-S10.

## Data Availability

The methylomes generated during this study have been deposited in the European Genome-Phenome Archive (EGA) with the accession number EGAS50000000085 (https://ega-archive.org/). The data support the findings of this study are available on reasonable request from the corresponding author Dr. Jun Dong. The data are not publicly available due to restrictions e.g. their containing information that could compromise the privacy of research participants. Code used for PMD analysis was the same from our previous work (10.1186/s13059-018-1510-5), and the remaining codes are freely available at source.
