# Supplementary material for: Methylomes of human CD4 and CD8 memory T lymphocytes reveal tissue-specific epigenetic signatures for maintenance and recall function
Source: Immun Inflamm. 2025 Oct 1;1(1):13. doi: 10.1007/s44466-025-00009-x (PMC12623507; doi:10.1007/s44466-025-00009-x)
Supplement: Supplementary file 1 — Supplementary Material 1: Materials and Methods. [file 44466_2025_9_MOESM1_ESM.pdf]

Materials and Methods

KEY RESOURCES TABLE

**REAGENTS**	SOURCE	IDENTIFIER
**Antibodies**		
anti-CD3 A700	Biolegend	Cat#300324; RRID: AB_493738
anti-CD3 APC-Cy7	Biolegend	Cat#344818; RRID: AB_10644011
anti-CD4 PE-Cyanine5.5	eBioscience	Cat#35-0047-42; RRID: AB_11218283
anti-CD8 BV785	Biolegend	Cat#301046; RRID: AB_2563264
anti-CD45 FITC	Biolegend	Cat#304006; RRID: AB_314394
anti-CD45RA BV570	Biolegend	Cat#304132; RRID: AB_314410
anti- CD45PE-Vio770	Miltenyi Biotec	Cat#130-110-639; RRID:AB_2658247
anti-CD45ROBrilliant Violet 650™	Biolegend	Cat#304231; RRID: AB_2563462
anti-CD69 Brilliant Violet 421™	Biolegend	Cat# 310930; RRID: AB_10933255
anti-CD69 PE-CF594	BD Biosciences	Cat# 562617; RRID:AB_2737680
anti-CD154 BV421	Biolegend	Cat#310824; RRID: AB_2562721
anti-CD154 APC	Miltenyi Biotec	Cat#130-113-610; RRID:AB_2733342
anti-CD14 Pacific Orange	House conjugate	Clone TM1
anti-CD19 Pacific Orange	House conjugate	Clone BU12
anti-CD137 FITC	eBioscience	Cat#11-1379-42; RRID:AB_1272070
anti-KI67 PE	eBioscience	Cat#12-5699-42; RRID: AB_10688373
anti-CD127 VioBright FITC	Miltenyi Biotec	Cat# 130-109-439; RRID: AB_2654830
anti-CD127 A488	Biolegend	Cat# 311313; RRID: AB_10895911
anti-CD25 APC	Miltenyi Biotec	Cat# 130-109-021; RRID: AB_2656665

anti-CD25 BV605	Biolegend	Cat#302632; RRID: AB_11218989
anti-CD38 PE-Cy7	Biolegend	Cat#303515; RRID: AB_1279235
anti-HLA-DR APC-Cy7	Biolegend	Cat# 307618; RRID: AB_493586
DAPI	Thermo Fisher Scientific	Ca# D1306
**Chemicals, Peptides, Proteins, and Others**		
Qiazol Lysis Reagent	Qiagen	Cat# 79306
Q5 High-Fidelity DNA Polymerase	New England Biolabs	Cat# M0491L
Uracil-DNA glycosilase (UDG)	New England Biolabs	Cat# M0280L
Human male Ab serum	Sigma-Aldrich	Cat# H4522-100ML
Live/Dead^{TM}Fixable Aqua Dead Cell Stain Kit (Pacific Orange)	ThermoFischer	L34957
Penicillin-Streptomycin-Glutamine (100X)	ThermoFischer	10378016
RPMI Medium 1640 - GlutaMax	ThermoFischer	Cat# 21875-091
RNAse inhibitor	ThermoFischer	Cat# N8080119
*Hae*III restriction enzyme	New England Biolabs	Cat# R0108M
Klenow fragment exo-	New England Biolabs	Cat# M0212M
T4-ligase	New England Biolabs	Cat# M0202M
AMPure XP beads	Beckham Coulter	Cat# A63881
**Critical Commercial Assays**		

EZ-DNA methylation Gold kit	Zymo research	Cat# D5005
**Deposited data**		
Multiplex reduced representation of bisulfite sequencing (mRRBS) data	EGA	EGAS50000000085 (https://ega-archive.org/)
**Software and algorithms**		
MACSQuantify^{TM} software	Miltenyi Biotec	https://www.miltenyibiotec.com/products/macsquantify-software.html#130-094-556
FlowJo 10 Software	BD Biosciences	https://www.flowjo.com
GraphPad Prism 5.04	GraphPad Software	https://www.graphpad.com
Adobe Illustrator	Adobe Systems	https://www.adobe.com
R version 4.2.1	Cran	https://cran.rstudio.com/
RStudio	Posit.co	https://posit.co/download/rstudio-desktop/
pheatmap (version 1.0.12)	CRAN	https://cran.r-project.org/web/packages/ pheatmap/index.html
ggplot2 (version 3.5.0)	CRAN	https://cran.r-project.org/web/packages/ggplot2/index.html
Metascape	Zhou et al.[1]	http://metascape.org/gp/index.html#/main/step1
Integrative Genomics Viewer software (version 2.16.2)	Robinson et al.[2]	http://www.broadinstitute.org/igv/
UCSC Genome Browser	University of California, Santa Cruz	http://genome.ucsc.edu
bcl2fastq	Illumina	https://emea.support.illumina.com/sequencing/sequencing_software/bcl2fastq-conversion-software.html
cutadapt (version 1.3)	GitHub	https://github.com/marcelm/cutadapt
Trim Galore! (version 0.4.2)	GitHub	https://github.com/FelixKrueger/TrimGalore

FLASh tool	The Center for Computational Biology at Johns Hopkins University	http://ccb.jhu.edu/software/FLASH/
HT software 22	BiQAnalyzer	http://biq-analyzer-ht.bioinf.mpi-inf.mpg.de/
hg38/GRCh38 reference genome (Consortium, 2015)	the University of California, Santa Cruz (UCSC)	https://hgdownload.soe.ucsc.edu/goldenPath/hg38/bigZips/
methylCtools (version 0.9.2)	GitHub	https://github.com/hovestadt/methylCtools
Samtools (version 1.3)	GitHub	https://github.com/samtools/samtools
Picard tools (version 1.115)	GitHub.	https://github.com/broadinstitute/picard
Bis-SNP (dbSNP, version 151)	National Center for Biotechnology Information	https://people.csail.mit.edu/dnaase/bissnp2011/ or: https://github.com/dnaase/Bis-tools
MethylSeekR (version 1.38.0)	Bioconductor	http://www.bioconductor.org/packages/release/bioc/html/MethylSeekR.html
Methylkit (Version 1.20.0)	Bioconductor	https://www.bioconductor.org/packages/release/bioc/html/methylKit.html
ChIPseeker (version 1.34.1)	Bioconductor	https://www.bioconductor.org/packages/release/bioc/html/ChIPseeker.html
MethReg (version 1.8.0)	Bioconductor	https://www.bioconductor.org/packages/release/bioc/html/MethReg.html
Limma package (version 3.54.2)	Bioconductor	https://www.bioconductor.org/packages/release/bioc/html/limma.html
**Other**		
FACSArial	BD Biosciences	N/A
gentleMACS™ dissociator	Miltenyi Biotec	N/A
HiSeq 2500	Illumina	https://www.illumina.com/
Qubit Fluorometer	Invitrogen	https://www.fishersci.de/shop/products/qubit-4-fluorometer/15723679

2100 Bioanalyzer	Agilent	https://www.agilent.com/en/product/automated-electrophoresis/bioanalyzer-systems/bioanalyzer-instrument
BioRender	BioRender	BioRender.com

RESOURCE AVAILABILITY

Lead contact

Further information and requests for resources and reagents should be directed to and will be fulfilled by the Lead Contact, Jun Dong, German Rheumatism Research Center, (dong@drfz.de).

Materials availability

This study did not generate new unique reagents.

Data and code availability

The methylomes generated during this study have been deposited in the European GenomePhenome Archive (EGA) with the accession number EGAS50000000085 (https://ega-archive.org/). The data support the findings of this study are available on reasonable request from the corresponding author Dr. Jun Dong. The data are not publicly available due to restrictions e.g. their containing information that could compromise the privacy of research participants. Code used for PMD analysis was the same from our previous work [3] and the remaining codes are freely available at source.

EXPERIMENTAL MODEL AND STUDY PARTICIPANT DETAILS

Human samples

Peripheral blood and/or paired tissue samples were collected from anonymous female adult donors without ongoing immune reactions (Table S1). Bone marrow samples were obtained from donors undergoing hip replacement operations. Spleen cells were isolated from previously cryopreserved spleen tissue samples obtained for diagnostic purpose. Skin samples were obtained from discarded tissues from healthy donors during plastic surgeries, and the lung and intestine samples were taken from the adjacent healthy parts of respective tissues removed during organ operations. Written informed consent in accordance with the Declaration of Helsinki was obtained from all study subjects with local ethical committee (Ethikkommission der Charité-Universitätsmedizin Belrin) approval (EA1/105/09).

METHOD DETAILS

Human peripheral blood and tissue sample collection and mononuclear cell isolation

Mononuclear cells of blood, bone marrow, and spleen were isolated by density gradient sedimentation using Ficoll-Hypaque (Sigma-Aldrich). Skin samples were prepared as described previously [4]. In brief, skin samples were initially washed two times with PBS buffer, and then the subcutaneous fat and hair were removed. This was followed by mechanical dissection into 2-4 mm fragments and digestion in cRPMI 1640 medium (supplemented with 100 U/mL penicillin, 100 µg/mL streptomycin, and 10% Ab serum) containing 1 mg/mL collagenase IV (Worthington) and 20

µg/mL DNAse I (Sigma) at 37 °C, 5% CO_2 for 12h. The digested fragments were dissociated using a gentleMACS™ dissociator (Miltenyi Biotec), followed by filtration through a 70-µm filter (Corning). Intestine samples were minced into 4mm small fragments and digested in cRPMI 1640 medium (as described above) containing 0.5 mg/mL collagenase II, 100 µg/mL DNAse I and 100 µg/ml trypsin inhibitor (Sigma) at 37 °C with a slow shaking (50 rpm) for 1h. The digested samples were filtered through a 70-µm filter, and the remaining fragments were dissociated using a gentleMACS™ dissociator and filtered again. For lung samples, minced tissue fragments were squeezed through a sterile sieve by the upside of a 25mL sterile syringe. Filtrates were centrifuged for 10min at 1000rpm and 4°C. The cell pellets were resuspended in a 40% Percoll (Ge Healthcare; in PBS) solution and loaded over a 80% Percoll solution, and centrifuged at room temperature with 1000 × *g* for 30min without brake. Afterwards, the interphase was transferred into a 50mL falcon tube through a 100-µm filter (Corning) and washed with cold PBS/BSA (0.5%) /EDTA (2mM) buffer. Remaining erythrocytes were lysed for 5min on ice using an erythrocyte lysis buffer (QIAGEN) and washed again with cold PBS/EDTA (2mM) buffer.

Flow cytometry and cell sorting

In this study, flow cytometry analysis was performed according to the guidelines described previously [5]. Single-cell suspension was stained with surface antibodies for 10 min at 4 °C. The fluorochrome-conjugated mouse anti-human antibodies used for flow cytometry experiments are listed in the above Key Resources Table. DAPI (1 µg/mL) was used as a dead cell exclusion marker. *Ex vivo* $CD4^+CD69^{+/-}$memory T-cell subsets were purified by fluorescence-activated cell sorting (FACS) using a BD FACSAria II cell sorter (BD Biosciences). During cell sorting, memory T cells were gated on $CD45^+CD3^+CD4^+/CD8^+$ and excluded for regulatory T cells and monocytes and B cells by gating on the $CD25^-DAPI^-CD14^-CD19^-$ ($DUMP^-$) cells. Thus, blood memory $CD4^+/CD8^+$ T cells were sorted as $CD45^+CD3^+DUMP^-CD25^-CD4^+/CD8^+CD45RO^+CD69^-$; tissue-derived memory $CD4^+$ T cells were sorted from each tissue as $CD45^+CD3^+DUMP^-CD25^-CD4^+/CD8^+CD45RO^+CD69^+$. Purity of the sorted populations was determined as <98% by post-sort check. Data were analyzed using the FlowJo V10 software (BD Biosciences).

Genomic DNA preparation

Genomic (g) DNA from purified cells was extracted directly from purified memory T cell subsets without prior *in vitro* stimulation, using the Qiagen DNeasy blood and tissue kit according to the manufacturers' protocol. The purity and concentration of the extracted gDNA were assessed by a Qubit fluorometer (Invitrogen). Equal amount of gDNA samples from the same tissue type of three donors were pooled as one replicate of that tissue type. Alternatively, blood or some tissue samples were isolated from individual donors without pooling and analyzed as replicate of indicated tissue type (detailed in Supplementary Table 1).

Multiplex reduced representation of bisulfite sequencing (RRBS)

Multiplex RRBS was applied for higher throughout and low amounts of input DNA according to the published procedure,[6] with minor modifications. In brief, 50-100 ng of gDNA was digested overnight at 37°C with 50 U *Hae*III restriction enzyme (NEB) in a 30-µL reaction. After A-tailing reaction with Klenow fragment exo-, sample-specific NGS adaptors were ligated using T4-DNA ligase. The libraries were then bisulfite converted with an EZ-DNA

methylation Gold kit and PCR amplified for 12-18 cycles using Hot Star Taq polymerase (Qiagen). After purification with Agencourt AMPure XP beads (Beckman Coulter), libraries went through quality checks (qPCR and Agilent Bioanalyzer) and were sequenced with 1x 100 nt single reads on an Illumina HiSeq 2500 system platform.

Mapping of multiplex RRBS data

Generated multiplex RRBS data were demultiplexed with bcl2fastq (Illumina) and then processed as described previously (Durek et al., 2016; Wang et al., 2013). In brief, the sequencing reads were trimmed with Trim Galore! (v0.4.2) (https://github.com/FelixKrueger/TrimGalore) for adapter contamination and 3' ends with base quality below 20 in RRBS mode. The resulting reads were aligned to the 1000 genomes version of the human hg38/GRCh38 reference genome (Consortium, 2015) using the BWA (v0.6.2) (Li and Durbin, 2010), and the wrapper methylCtools (v0.9.2), Samtools (v1.3) (Li et al., 2009) and Picard tools (v1.115) ("Picard Tools - By Broad Institute" 2017) for converting, merging and indexing of alignment files. Bis-SNP was used for SNP (dbSNP, v138 or v151, National Center for Biotechnology Information, National Library of Medicine 2013) aware re-alignment, quality recalibration and methylation calls.

Genome segmentation analysis of DNA methylation patterns in Tm populations across tissues

We utilized MethylSeekR (version 1.38.0) to segment the genome based on DNA methylation patterns, leveraging predefined genome segments as reference. In particular, partial methylated domains (PMDs), were previously defined by us using effector memory T cells (Tem) from blood samples of CD4 [7] and CD8 [3] T cells, predominantly CD69$^-$. Since in the previous study involving CD4$^+$ Tem reference, bone marrow CD69$^+$ subsets exhibited similar positioning in PC1, indicating a shared proliferation history, we employed these reference segments to directly calculate the methylation levels of CpG sites within our RRBS data from CD4 and CD8 Tm populations across various tissues and blood samples. In this analysis, we averaged the methylation levels of CD69$^+$ and CD69$^-$ subsets for tissues separately, while for blood samples, methylation levels were averaged only for CD69$^-$ cells [3].

Calling of DNA methylation values and identification of differentially methylated regions (DMRs)

From qualified primary data, 164105 sites were removed due to their overlap with SNPs. To eliminate the effect of sex-specific methylation, sites from Y-chromosome were also removed from further analysis. Beta-values of 500-bp genomic tiles of 22 memory CD4 and CD8 T cell subsets isolated from the bone marrow, spleen, lung, intestine, skin and blood samples were calculated using R (Version 4.2.1) and Methylkit package (Version 1.20.0) [8]. Beta values range from 0 to 1 and represent methylation values from 0% to 100%, respectively. DMRs were detected in de-novo mode with treating missing sites as zero-covered sites in the aggregation step of the tiled analysis. We searched for DMRs with ≥5 CpGs covered within 500-bp tiles using default setting. After merging both strands, sites were filtered to retain only those with an average of per-CpG coverage of at least 3 x, corresponding to a minimum of 15x total coverage per region. DMRs were defined based on a q-value < 0.05and a minimum methylation difference of ≥ 15% between any two comparison groups. On average, >95% of all identified DMRs sets met these criteria. Mitochondria chromosomes were excluded.

The mRRBS data of $CD69^+$ memory $CD4^+$ T cells from bone marrow and spleen samples were taken from our previous work and from the European Genome-Phenome Archive under the accession numbers EGAS0000100624 [7] and EGAS00001005475 [9]. Overall, analysis was conducted on beta-values of 667400 tiles from $CD4^+$ samples and 776782 tiles from $CD8^+$ samples. In this study, 52 methylome data sets were generated and compared with these published four methylome data sets.

Principal-component analysis

Principal Component Analysis (PCA) was performed using the built-in function prcomp in R. The data was processed to extract principal components, representing the major patterns of methylation variability. The results were then visualized using the ggplot2 package (version 3.5.0) to create a scatter plot, distributing the distribution and relationships of the data points in reduced-dimensional space, i.e. PC1 versus PC2 in this study.

Genomic features

The identified DMRs were related to gene locations with five gene structure prioritized categories: 5'UTRs, promoters (+/- 3kb from transcription start sites (TSSs)), exons, introns, 3'UTR, downstream, and intergenic regions, using hg38. The resulting DMRs were annotated using the R package ChIPseeker (version 1.34.1) and their intersection with putative transcription factor binding site(s) (TFBS) were annotated using the R package MethReg (version 1.8.0).

Heatmap analysis

In this study, a set of DMRs or a set of genes associated with various functional categories, including chemokine receptors, transcription factors, and others, was extracted as promoter/intron DMRs from the compiled DMRs specific to $CD4^+$ and $CD8^+$ Trm populations. Unsupervised heatmap analysis was then employed to visualize and interpret patterns within the dataset. Heatmaps were generated using the pheatmap package (verson 1.0.12) in R programming language, with rows and columns representing variables or tissue types. The color intensity in the heatmap indicated the strength or direction of the relationships between variables, facilitating the identification of trends and clusters within the data.

DMR-linked gene enrichment analysis using Metascape

Top 3000 genes from compiled $CD69^+$ and $CD69^-$ tissue DMRs were subjected to gene enrichment analysis using Metascape [1]. The species "Homo sapiens" was selected for analysis. Default setting were used, with a *p*-value cutoff of 0.01 and false discovery rate (FDR) correction. Enrichment analysis included most available gene set databases (e.g., GO Biological Processes, KEGG Pathways, Reactome Gene Sets). Enrichment readouts provided by Metascape were reviewed to identify significantly enriched pathways, biological processes, and molecular functions. Comparisons were made to identify shared and unique enriched pathways between tissue $CD69^+$ and $CD69^-$ of CD4 and CD8 T cells. Metascape's visualization tools were used to generate enrichment heatmaps.

Visualization of differentially methylated regions by the Integrative Genomics Viewer (IGV)

To assess and visualize the distribution of DMRs across the genome, we utilized the Integrative Genomics Viewer (IGV) version 2.16.2. Preprocessed and normalized methylation data, including DMRs identified through Methylkit,

were loaded into IGV for visualization. Methylation percentage data in bigWig format were used. The human hg38 genome assembly was selected within IGV for alignment and annotation. IGV was configured to display DNA methylation levels as tracks, allowing for the simultaneous visualization of multiple samples from different tissues. Tracks were color-coded for clarity, with customizable settings for track height and display options. Additionally, to gain insights into genomic regions conserved across species, we incorporated the hg38.phastCons4way track (available at UCSC) as a secondary reference. This dataset includes alignments of the human genome with those of the mouse (Mus musculus), Chinese tree shrew (Tupaia chinensis), and Malayan flying lemur (Galeopterus variegatus).

Methylation analysis of DMRs between tissues

To assess methylation differences between the analyzed groups, we calculated CpG beta values for the differentially methylated regions (DMRs) of interest. Beta values were computed by merging replicate values within each group. Statistical significance of methylation differences between groups was determined using the Wilcoxon rank-sum test, implemented in R (Version: 4.2.3). The results were visualized using box plots, which display the distribution of beta values across groups.

Correlation between DMRs with associated gene expression

Annotated DMRs were included for correlation with corresponding gene expressions by $CD4^+$ Tm. Four gene expression datasets were obtained from the Gene Expression Omnibus (GEO) database. Expression datasets GSE50677[10], GSE94964[11], GSE131770[12], and GSE49877[13] were used to correlate with bone marrow versus blood DMRs, spleen or lung versus blood DMRs, skin versus blood DMRs, and intestine versus blood DMRs, respectively. Microarray data (bone marrow, intestine) were converted back to linear scale by reversing $\log_2$ transformation. RNA-seq data were processed using either DESeq2 normalization (spleen, lung; raw counts scaled by size factors, without log transformation) or counts per million (skin; CPM, not log-transformed). All expression values are presented as non-log-transformed gene expression levels.

Correlation analysis between DNA methylation and gene expression levels in tissue versus blood samples was performed using a built-in simple linear regression model in GraphPad Prism.

QUANTIFICATION AND STATISTICAL ANALSIS

Statistical analysis was performed using R (Version: 4.2.3). MethylSeekR (version 1.38.0) was applied to identify differences between paired comparisons of various tissues and blood. Adjust *p*-values less than 0.05 were considered statistically significant. The data matrix was normalized using z-score normalization. Both Manhattan and Euclidean distance matrices were calculated to analyze the similarities and relationships between tissues or methylation regions. For statistical details, please refer to the respective figure legends.

REFERENCES

[1] Y. Zhou, B. Zhou, L. Pache, M. Chang, A. H. Khodabakhshi, O. Tanaseichuk, C. Benner, and S. K. Chanda. 2019. Metascape provides a biologist-oriented resource for the analysis of systems-level datasets. *Nat Commun* 10, 1, (20190403), https://doi.org/ 10.1038/s41467-019-09234-6

[2] J. T. Robinson, H. Thorvaldsdóttir, W. Winckler, M. Guttman, E. S. Lander, G. Getz, and J. P. Mesirov. 2011. Integrative genomics viewer. *Nat Biotechnol* 29, 1, https://doi.org/ 10.1038/nbt.1754

[3] A. Salhab, K. Nordstrom, G. Gasparoni, K. Kattler, P. Ebert, F. Ramirez, L. Arrigoni, F. Muller, J. K. Polansky, C. Cadenas, G. Hengstler J, T. Lengauer, T. Manke, Deep Consortium, and J. Walter. 2018. A comprehensive analysis of 195 DNA methylomes reveals shared and cell-specific features of partially methylated domains. *Genome Biol* 19, 1, (20180928), https://doi.org/ 10.1186/s13059-018-1510-5

[4] W. Du, D. Lenz, R. Kohler, E. Zhang, C. Cendon, J. Li, M. Massoud, J. Wachtlin, J. Bodo, A. E. Hauser, A. Radbruch, and J. Dong. 2021. Rapid Isolation of Functional ex vivo Human Skin Tissue-Resident Memory T Lymphocytes. *Front Immunol* 12(2021/04/09), https://doi.org/ 10.3389/fimmu.2021.624013

[5] A. Cossarizza, H. D. Chang, A. Radbruch, S. Abrignani, R. Addo, M. Akdis, I. Andra, F. Andreata, F. Annunziato, E. Arranz, P. Bacher, S. Bari, V. Barnaba, J. Barros-Martins, D. Baumjohann, C. G. Beccaria, D. Bernardo, D. A. Boardman, J. Borger, C. Bottcher, L. Brockmann, M. Burns, D. H. Busch, G. Cameron, I. Cammarata, A. Cassotta, Y. Chang, F. G. Chirdo, E. Christakou, L. Cicin-Sain, L. Cook, A. J. Corbett, R. Cornelis, L. Cosmi, M. S. Davey, S. De Biasi, G. De Simone, G. Del Zotto, M. Delacher, F. Di Rosa, J. Di Santo, A. Diefenbach, J. Dong, T. Dorner, R. J. Dress, C. A. Dutertre, S. B. G. Eckle, P. Eede, M. Evrard, C. S. Falk, M. Feuerer, S. Fillatreau, A. Fiz-Lopez, M. Follo, G. A. Foulds, J. Frobel, N. Gagliani, G. Galletti, A. Gangaev, N. Garbi, J. A. Garrote, J. Geginat, N. A. Gherardin, L. Gibellini, F. Ginhoux, D. I. Godfrey, P. Gruarin, C. Haftmann, L. Hansmann, C. M. Harpur, A. C. Hayday, G. Heine, D. C. Hernandez, M. Herrmann, O. Hoelsken, Q. Huang, S. Huber, J. E. Huber, J. Huehn, M. Hundemer, W. Y. K. Hwang, M. Iannacone, S. M. Ivison, H. M. Jack, P. K. Jani, B. Keller, N. Kessler, S. Ketelaars, L. Knop, J. Knopf, H. F. Koay, K. Kobow, K. Kriegsmann, H. Kristyanto, A. Krueger, J. F. Kuehne, H. Kunze-Schumacher, P. Kvistborg, I. Kwok, D. Latorre, D. Lenz, M. K. Levings, A. C. Lino, F. Liotta, H. M. Long, E. Lugli, K. N. MacDonald, L. Maggi, M. K. Maini, F. Mair, C. Manta, R. A. Manz, M. F. Mashreghi, A. Mazzoni, J. McCluskey, H. E. Mei, F. Melchers, S. Melzer, D. Mielenz, L. Monin, L. Moretta, G. Multhoff, L. E. Munoz, M. Munoz-Ruiz, F. Muscate, A. Natalini, K. Neumann, L. G. Ng, A. Niedobitek, J. Niemz, L. N. Almeida, S. Notarbartolo, L. Ostendorf, L. J. Pallett, A. A. Patel, G. I. Percin, G. Peruzzi, M. Pinti, A. G. Pockley, K. Pracht, I. Prinz, I. Pujol-Autonell, N. Pulvirenti, L. Quatrini, K. M. Quinn, H. Radbruch, H. Rhys, M. B. Rodrigo, C. Romagnani, C. Saggau, S. Sakaguchi, F. Sallusto, L. Sanderink, I. Sandrock, C. Schauer, A. Scheffold, H. U. Scherer, M. Schiemann, F. A. Schildberg, K. Schober, J. Schoen, W. Schuh, T. Schuler, A. R. Schulz, S. Schulz, J. Schulze, S. Simonetti, J. Singh, K. M. Sitnik, R. Stark, S. Starossom, C. Stehle, F. Szelinski, L. Tan, A. Tarnok, J. Tornack, T. I. M. Tree, J. J. P. van Beek, W. van de Veen, K. van Gisbergen, C. Vasco, N. A. Verheyden, A. von Borstel, K. A. Ward-Hartstonge, K. Warnatz, C. Waskow, A. Wiedemann, A. Wilharm, J. Wing, O. Wirz, J. Wittner, J. H. M. Yang, and J. Yang. 2021. Guidelines for the use of flow cytometry and cell sorting in immunological studies (third edition). *Eur J Immunol* 51, 12, (2021/12/16), https://doi.org/ 10.1002/eji.202170126

[6] Patrick Boyle, Kendell Clement, Hongcang Gu, Zachary D Smith, Michael Ziller, Jennifer L Fostel, Laurie Holmes, Jim Meldrim, Fontina Kelley, and Andreas Gnirke. 2012. Gel-free multiplexed reduced representation bisulfite sequencing for large-scale DNA methylation profiling. *Genome biology* 13, 10,

[7] P. Durek, K. Nordstrom, G. Gasparoni, A. Salhab, C. Kressler, M. de Almeida, K. Bassler, T. Ulas, F. Schmidt, J. Xiong, P. Glazar, F. Klironomos, A. Sinha, S. Kinkley, X. Yang, L. Arrigoni, A. D. Amirabad, F. B. Ardakani, L. Feuerbach, O. Gorka, P. Ebert, F. Muller, N. Li, S. Frischbutter, S. Schlickeiser, C. Cendon, S. Frohler, B. Felder, N. Gasparoni, C. D. Imbusch, B. Hutter, G. Zipprich, Y. Tauchmann, S. Reinke, G. Wassilew, U. Hoffmann, A. S. Richter, L. Sieverling, Deep Consortium, H. D. Chang, U. Syrbe, U. Kalus, J. Eils, B. Brors, T. Manke, J. Ruland, T. Lengauer, N. Rajewsky, W. Chen, J. Dong, B. Sawitzki, H. R. Chung, P. Rosenstiel, M. H. Schulz, J. L. Schultze, A. Radbruch, J. Walter, A. Hamann, and J. K. Polansky. 2016.

Epigenomic Profiling of Human CD4+ T Cells Supports a Linear Differentiation Model and Highlights Molecular Regulators of Memory Development. *Immunity* 45, 5, https://doi.org/ 10.1016/j.immuni.2016.10.022

[8] A. Akalin, M. Kormaksson, S. Li, F. E. Garrett-Bakelman, M. E. Figueroa, A. Melnick, and C. E. Mason. 2012. methylKit: a comprehensive R package for the analysis of genome-wide DNA methylation profiles. *Genome Biol* 13, 10, (2012/10/05), https://doi.org/ 10.1186/gb-2012-13-10-r87

[9] C. Cendon, W. Du, P. Durek, Y. C. Liu, T. Alexander, L. Serene, X. Yang, G. Gasparoni, A. Salhab, K. Nordstrom, T. Lai, A. R. Schulz, A. Rao, G. A. Heinz, A. L. Stefanski, A. Claussnitzer, K. Siewert, T. Dorner, H. D. Chang, H. D. Volk, C. Romagnani, Z. Qin, S. Hardt, C. Perka, S. Reinke, J. Walter, M. F. Mashreghi, K. Thurley, A. Radbruch, and J. Dong. 2022. Resident memory CD4+ T lymphocytes mobilize from bone marrow to contribute to a systemic secondary immune reaction. *Eur J Immunol* (2022/03/05), https://doi.org/ 10.1002/eji.202149726

[10] A. Okhrimenko, J. R. Grun, K. Westendorf, Z. Fang, S. Reinke, P. von Roth, G. Wassilew, A. A. Kuhl, R. Kudernatsch, S. Demski, C. Scheibenbogen, K. Tokoyoda, M. A. McGrath, M. J. Raftery, G. Schonrich, A. Serra, H. D. Chang, A. Radbruch, and J. Dong. 2014. Human memory T cells from the bone marrow are resting and maintain long-lasting systemic memory. *Proc Natl Acad Sci U S A* 111, 25, https://doi.org/ 10.1073/pnas.1318731111

[11] B. V. Kumar, W. Ma, M. Miron, T. Granot, R. S. Guyer, D. J. Carpenter, T. Senda, X. Sun, S. H. Ho, H. Lerner, A. L. Friedman, Y. Shen, and D. L. Farber. 2017. Human Tissue-Resident Memory T Cells Are Defined by Core Transcriptional and Functional Signatures in Lymphoid and Mucosal Sites. *Cell Rep* 20, 12, (2017/09/21), https://doi.org/ 10.1016/j.celrep.2017.08.078

[12] M. M. Klicznik, P. A. Morawski, B. Hollbacher, S. R. Varkhande, S. J. Motley, L. Kuri-Cervantes, E. Goodwin, M. D. Rosenblum, S. A. Long, G. Brachtl, T. Duhen, M. R. Betts, D. J. Campbell, and I. K. Gratz. 2019. Human CD4(+)CD103(+) cutaneous resident memory T cells are found in the circulation of healthy individuals. *Sci Immunol* 4, 37, (2019/07/07), https://doi.org/ 10.1126/sciimmunol.aav8995

[13] T. Raine, J. Z. Liu, C. A. Anderson, M. Parkes, and A. Kaser. 2015. Generation of primary human intestinal T cell transcriptomes reveals differential expression at genetic risk loci for immune-mediated disease. *Gut* 64, 2, (20140505), https://doi.org/ 10.1136/gutjnl-2013-306657
