# Supplementary material for: Methylomes of human CD4 and CD8 memory T lymphocytes reveal tissue-specific epigenetic signatures for maintenance and recall function
Source: Immun Inflamm. 2025 Oct 1;1(1):13. doi: 10.1007/s44466-025-00009-x (PMC12623507; doi:10.1007/s44466-025-00009-x)
Supplement: Supplementary file 2 — Supplementary Material 2: Fig. S1-S4, related to Fig. 1 and Tables S1 and S2. Fig. S5, related to Fig. 2 and Table S3. Fig. S6, related to Fig. 3 and Table S4. Fig. S7, related to Table S5. Fig. S8, related to Fig. 4 and Table S6. Fig. S9, related to Fig. 5 and Table S7. Fig. S11, related to Table S8. Fig. S12, related to Table S9. [file 44466_2025_9_MOESM2_ESM.zip › SI_Figures_Legends_R2.pdf]

Fig. S1

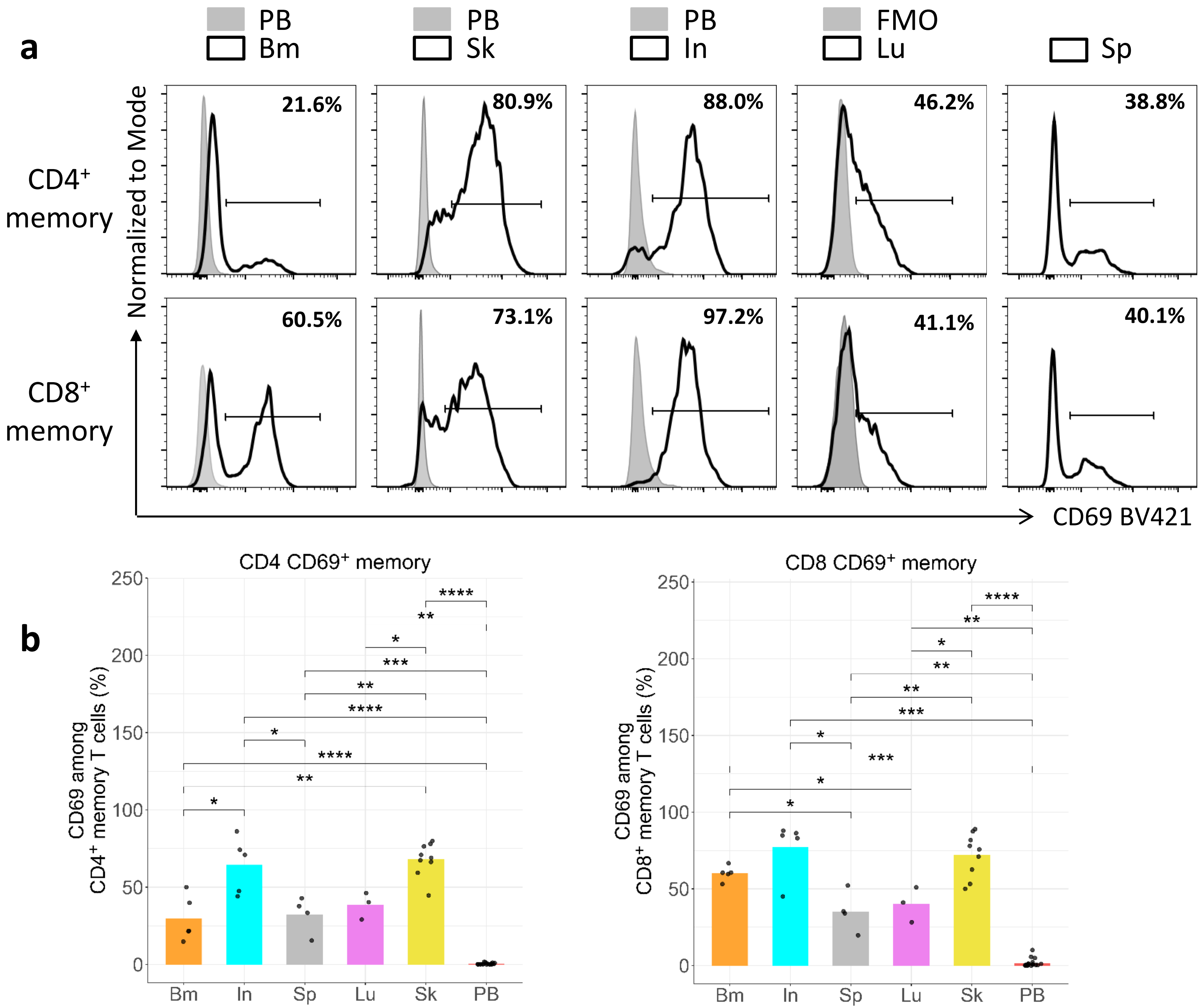
a
PB
Bm
PB
Sk
PB
In
FMO
Lu
Sp
CD4+ memory
CD8+ memory
Normalized to Mode
21.6%
80.9%
88.0%
46.2%
38.8%
60.5%
73.1%
97.2%
41.1%
40.1%
CD69 BV421
b
CD4 CD69+ memory
CD8 CD69+ memory
CD69 among CD4+ memory T cells (%)
CD69 among CD8+ memory T cells (%)
Bm
In
Sp
Lu
Sk
PB

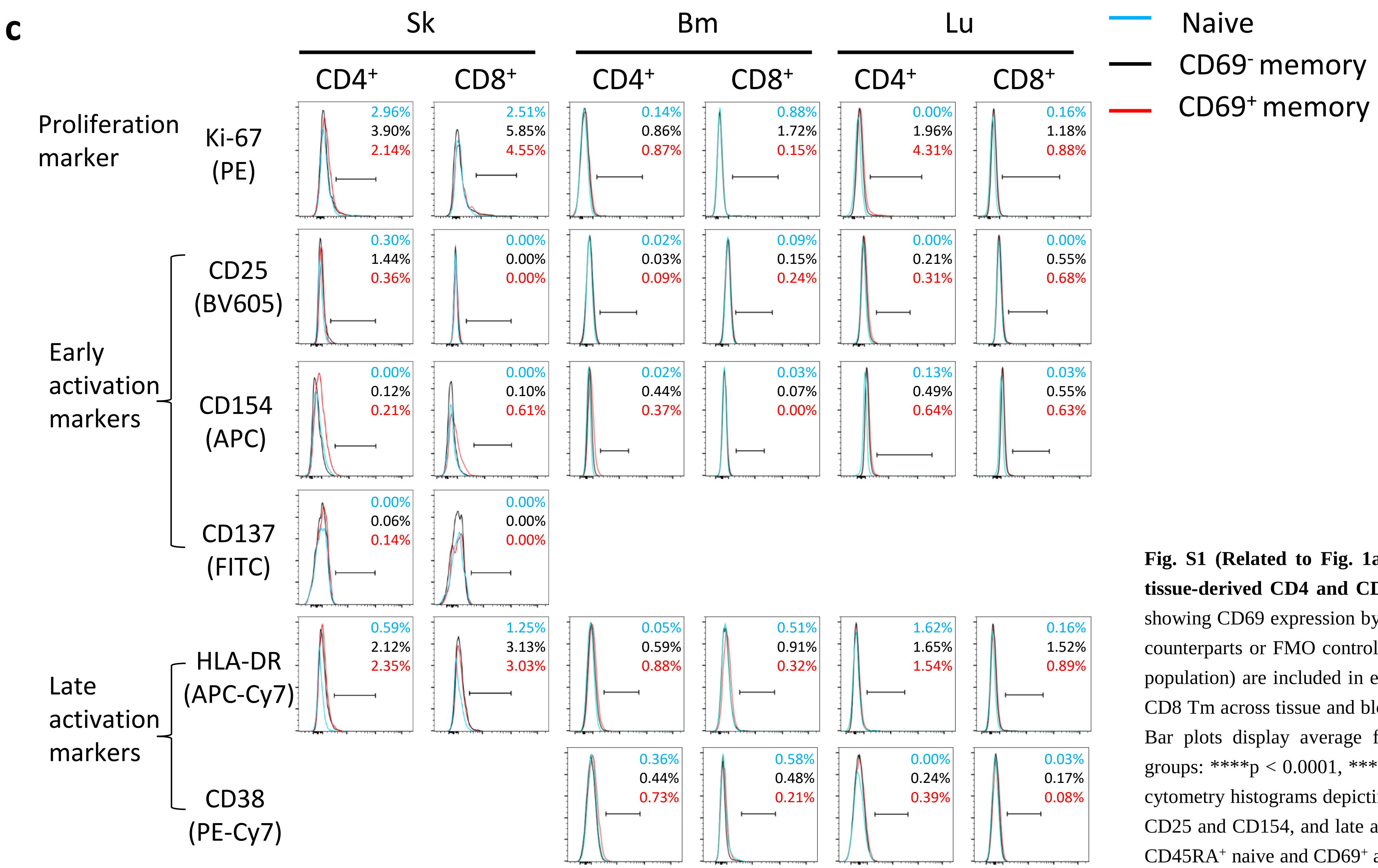

Fig. S1 (Related to Fig. 1a) Selective expression of CD69 by human ex vivo tissue-derived CD4 and CD8 Tm. (**a**) Representative flow cytometry histograms showing CD69 expression by human ex vivo tissue-derived Tm (lines) versus blood counterparts or FMO controls (grey areas). The number of positive cells (% parent population) are included in each plot. (**b**) Average of $CD69^{+}$ cells among CD4 and CD8 Tm across tissue and blood samples. Each dot represents an individual sample. Bar plots display average frequencies with Wilcoxon test significance between groups: ****$p < 0.0001$, *** $p < 0.001$, ** $p < 0.01$, * $p < 0.05$. (**c**) Exemplar flow cytometry histograms depicting proliferation marker KI-67, early activation markers CD25 and CD154, and late activation markers such as HLA-DR on indicated tissue $CD45RA^{+}$ naive and $CD69^{+}$ and $CD69^{-}$ CD4 Tm subsets.

Fig. S2

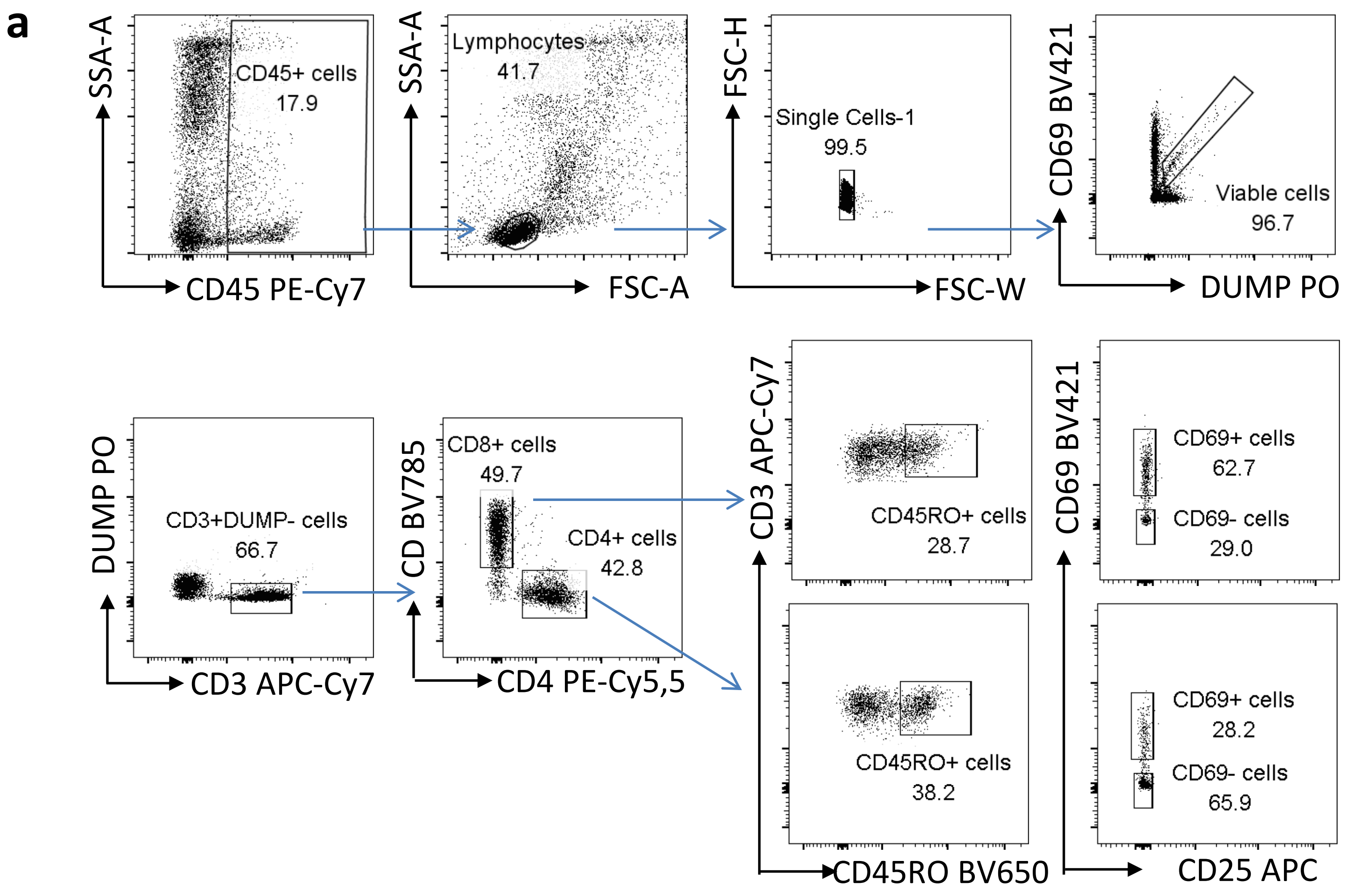
a
SSA-A
CD45+ cells
17.9
CD45 PE-Cy7
SSA-A
Lymphocytes
41.7
FSC-A
FSC-H
Single Cells-1
99.5
FSC-W
CD69 BV421
Viable cells
96.7
DUMP PO
DUMP PO
CD3+DUMP- cells
66.7
CD3 APC-Cy7
CD BV785
CD8+ cells
49.7
CD4+ cells
42.8
CD4 PE-Cy5,5
CD3 APC-Cy7
CD45RO+ cells
28.7
CD45RO+ cells
38.2
CD45RO BV650
CD69 BV421
CD69+ cells
62.7
CD69- cells
29.0
CD69+ cells
28.2
CD69- cells
65.9
CD25 APC

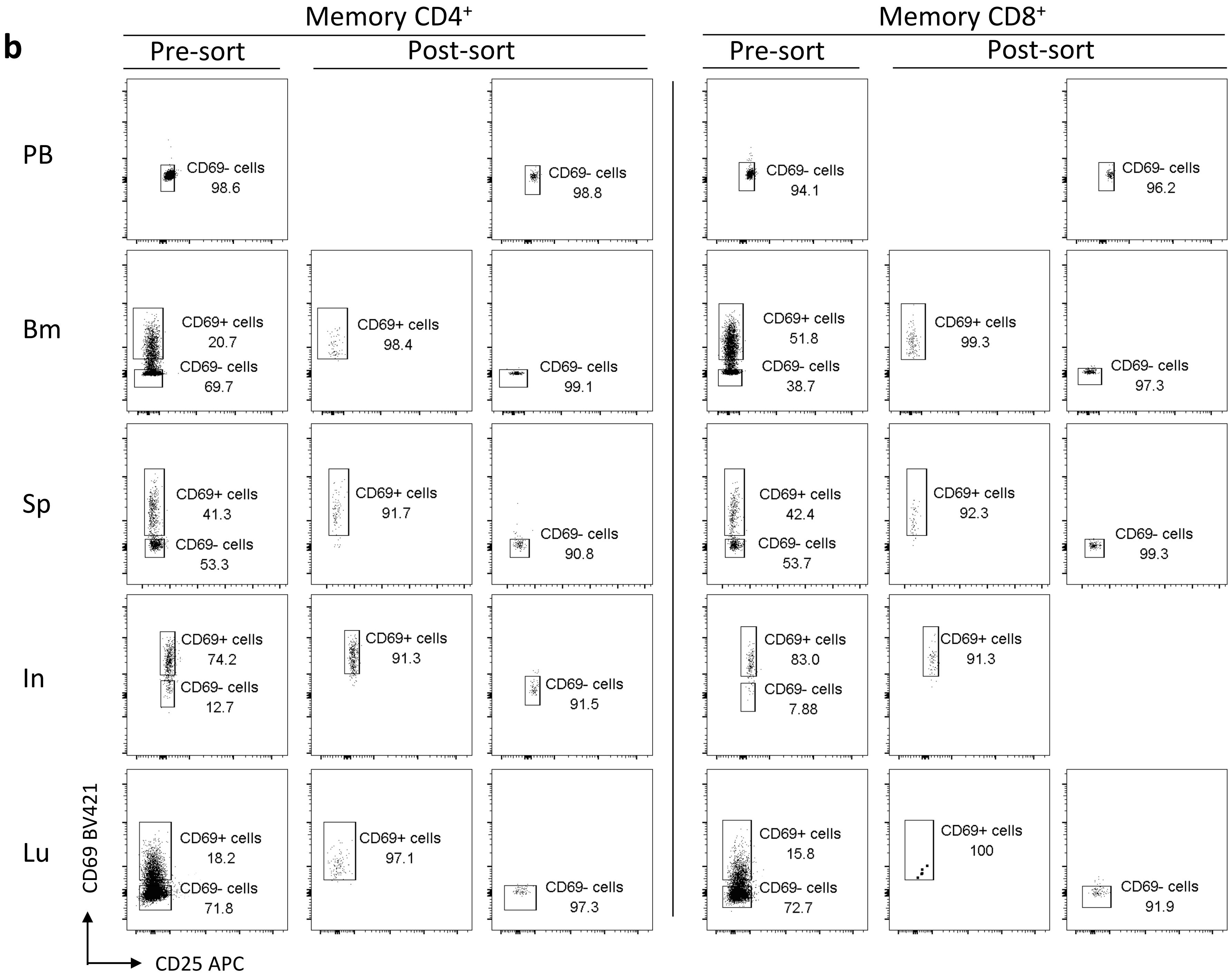
b
Memory CD4+
Pre-sort
Post-sort
Memory CD8+
Pre-sort
Post-sort
PB
Bm
Sp
In
Lu
CD69- cells 98.6
CD69- cells 98.8
CD69- cells 94.1
CD69- cells 96.2
CD69+ cells 20.7
CD69- cells 69.7
CD69+ cells 98.4
CD69- cells 99.1
CD69+ cells 51.8
CD69- cells 38.7
CD69+ cells 99.3
CD69- cells 97.3
CD69+ cells 41.3
CD69- cells 53.3
CD69+ cells 91.7
CD69- cells 90.8
CD69+ cells 42.4
CD69- cells 53.7
CD69+ cells 92.3
CD69- cells 99.3
CD69+ cells 74.2
CD69- cells 12.7
CD69+ cells 91.3
CD69- cells 91.5
CD69+ cells 83.0
CD69- cells 7.88
CD69+ cells 91.3
CD69+ cells 18.2
CD69- cells 71.8
CD69+ cells 97.1
CD69- cells 97.3
CD69+ cells 15.8
CD69- cells 72.7
CD69+ cells 100
CD69- cells 91.9
CD69 BV421
CD25 APC

Fig. S2 (Related to Fig. 1) Isolation of Tm subsets from multiple human tissues and blood. (**a** and **b**) Representative flow cytometry gating strategy (**a**) and purity check plots (**b**) depicting CD69 versus CD25 expression on CD4 Tm before and after isolation of $CD69^{+}$ and $CD69^{-}$ cell subsets, both of which are $CD25^{-}$. The number of positive and/or negative cells (% of parent populations) is included in each plot. Sequential inclusive gates were applied to select $CD45^{+}$, lymphocytes, and live cells. A CD69 vs. DUMP marker exclusion gate was used to remove only events coexpressing CD69 and DUMP markers (diagonal population: $CD3^{-}$, $CD19^{+}$, and $CD14^{+}$). Events within the $CD69^{+}DUMP^{-}$, $CD69^{-}DUMP^{+}$, or $CD69^{-}DUMP^{-}$ quadrants were retained for downstream analysis.

Fig. S3

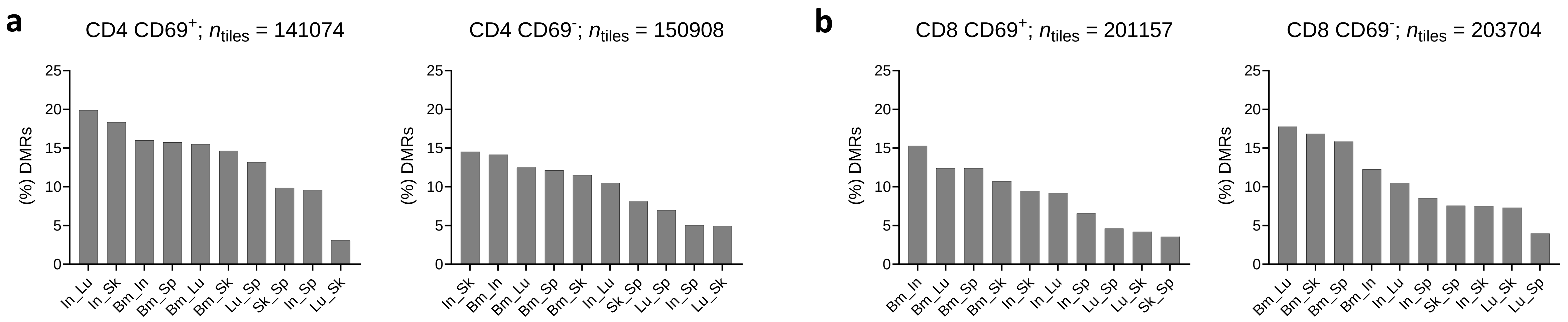
a
CD4 CD69+; ntiles = 141074
(%) DMRs
0
5
10
15
20
25
In_Lu
In_Sk
Bm_In
Bm_Sp
Bm_Lu
Bm_Sk
Lu_Sp
Sk_Sp
In_Sp
Lu_Sk
CD4 CD69-; ntiles = 150908
In_Sk
Bm_In
Bm_Lu
Bm_Sp
Bm_Sk
In_Lu
Sk_Sp
Lu_Sp
In_Sp
Lu_Sk
b
CD8 CD69+; ntiles = 201157
Bm_In
Bm_Lu
Bm_Sp
Bm_Sk
In_Sk
In_Lu
In_Sp
Lu_Sp
Lu_Sk
Sk_Sp
CD8 CD69-; ntiles = 203704
Bm_Lu
Bm_Sk
Bm_Sp
Bm_In
In_Lu
In_Sp
Sk_Sp
In_Sk
Lu_Sk
Lu_Sp

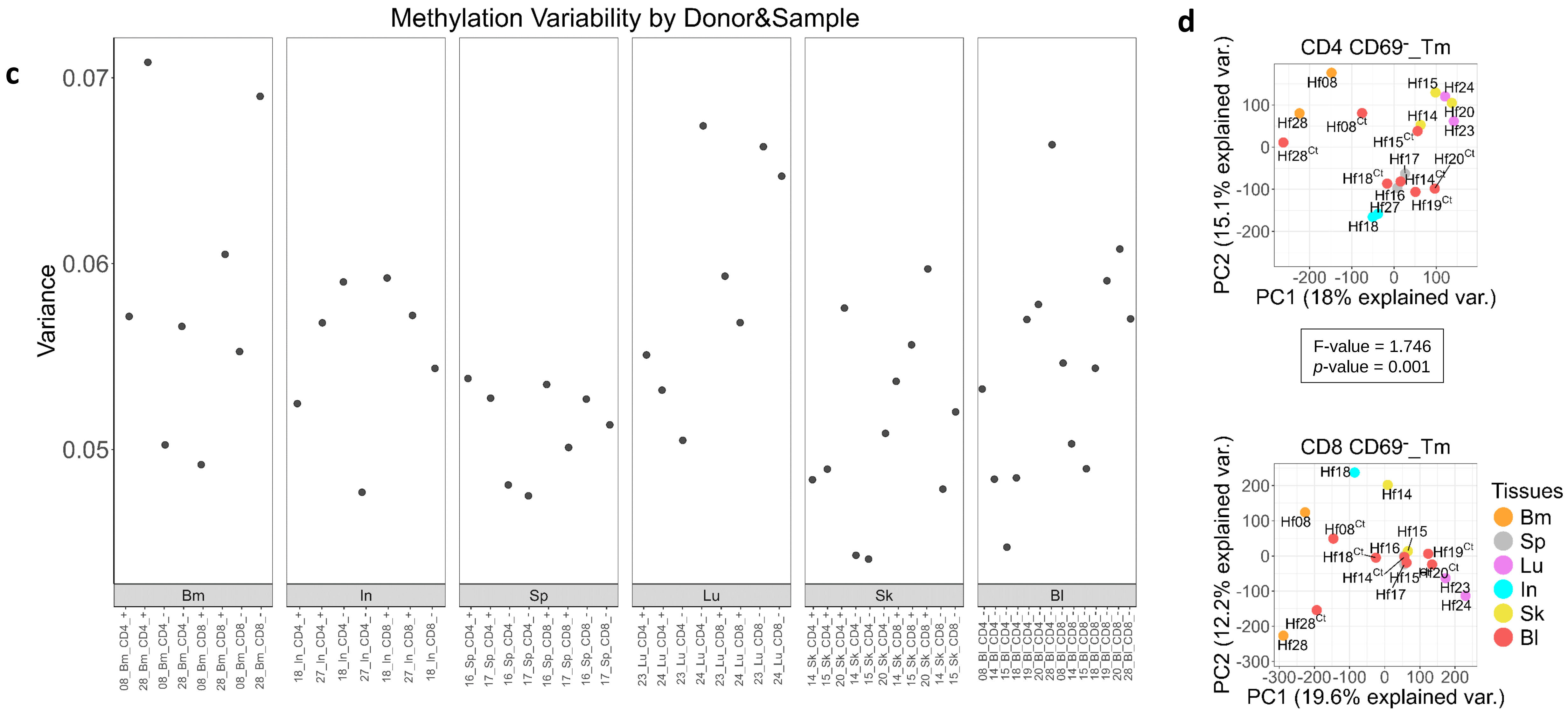

Fig. S3 (**Related to Fig. 1c**) **(a** and **b) Quantification of methylation differences between Tm populations in tissues and/or blood.** The percentage of DMRs among all qualified tiles for each indicated pairwise comparisons between CD69^{+} and CD69^{-} populations in CD4 (**a**) and CD8 (**b**) Tm lineages. (**c**) Interindividual differences in DNA methylation (of those in **c**) across Tm subsets. Variance in DNA methylation (corresponding to the data shown in panel **c**) is displayed for each Tm subset per donor. '+' and '−' refer to CD69 expression, indicating tissue-CD69^{+} and CD69^{-} and blood CD69^{-} Tm. (**d**) PCA plots of CD69^{-} CD4 and CD8 tissue Tm populations, including blood counterparts (labelled as Ct; >98% CD69-) for comparison. Each PCA was generated from DMRs derived exclusively from pairwise comparisons as in **a** and **b**. MANOVA results (F- and P-values) for tissue groups are shown on each plot. Replicates from each tissue types are indicated.

Fig. S4

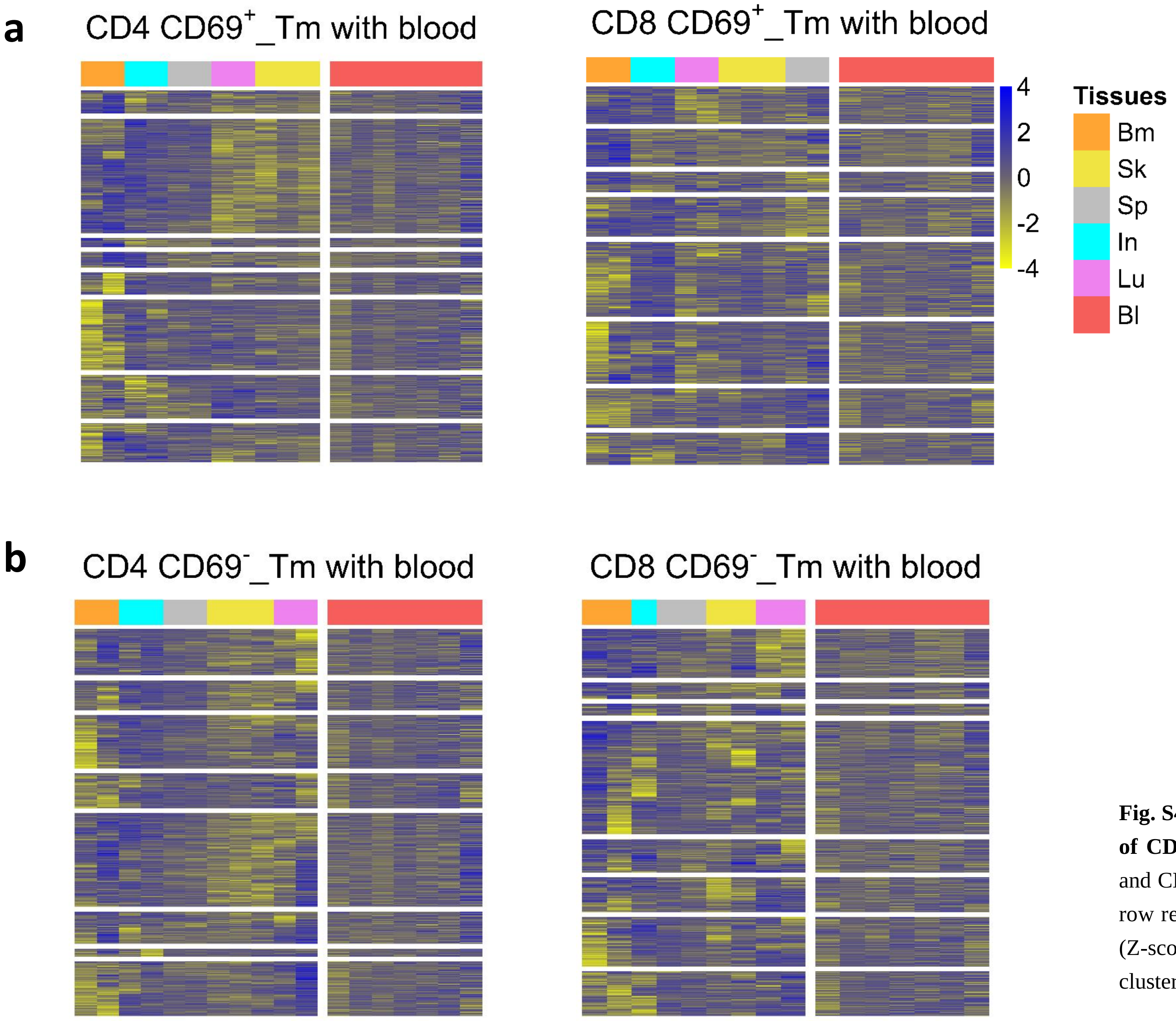

Fig. S4 (**Related to Fig. 1e) Blood-derived Tm data related to tissue-specific methylome patterns of CD69+ and CD69- Tm populations. (a** and **b)** Methylation patterns of blood-derived CD4 (**a**) and CD8 (**b**) Tm populations based on the values of tissue-specific DMRs described in Fig. 1e. Each row represents a DMR and each column a sample. Colors indicate relative DNA methylation levels (Z-score scaled across samples). White lines separate clusters of DMRs identified by hierarchical clustering.

Fig. S5

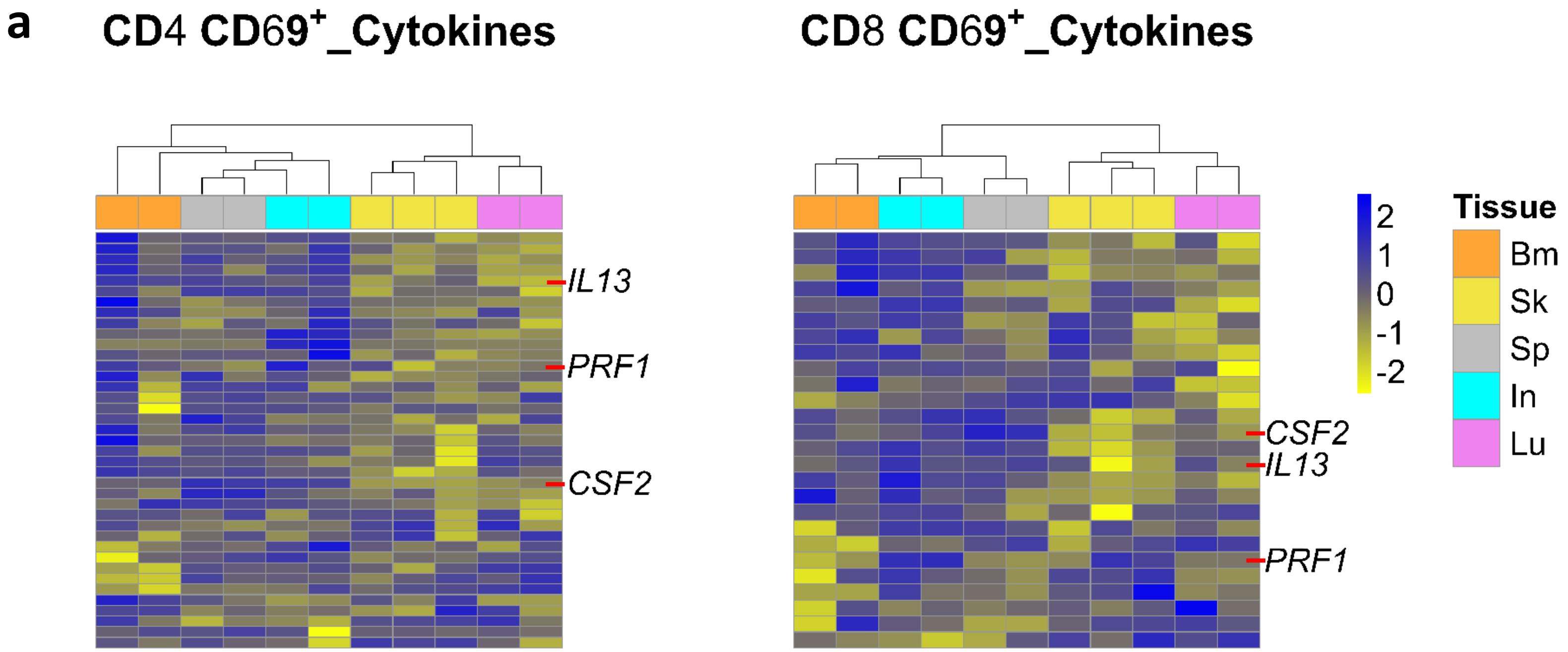
a
CD4 CD69+_Cytokines
IL13
PRF1
CSF2
CD8 CD69+_Cytokines
CSF2
IL13
PRF1
2
1
0
-1
-2
Tissue
Bm
Sk
Sp
In
Lu

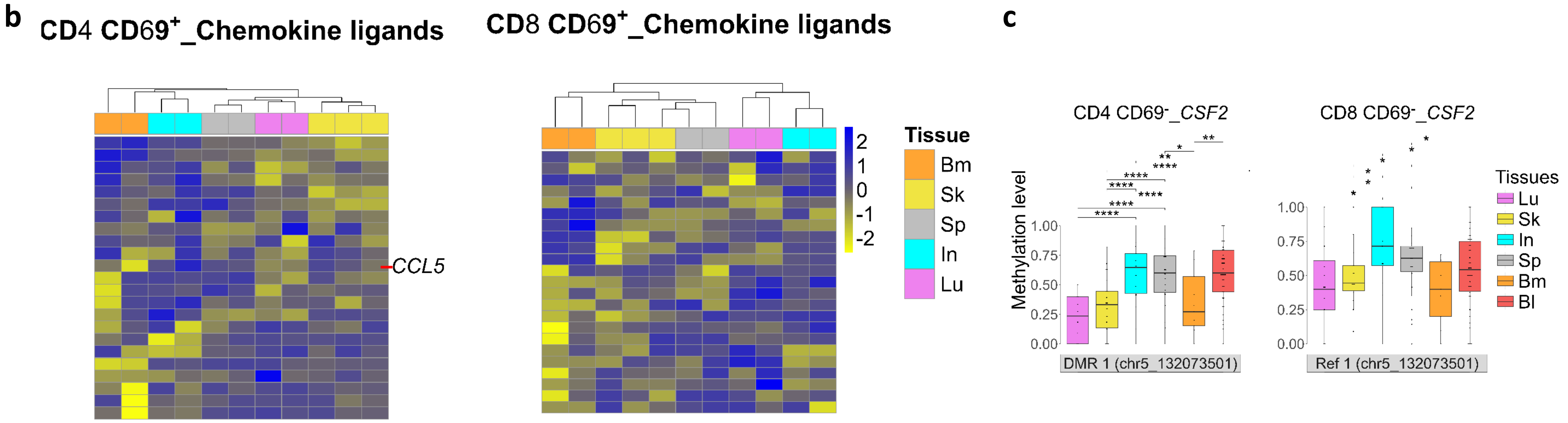

Fig. S5 (Related to Fig. 2 and Table S3) Epigenetic imprinting of silent Th/Tc effector genes in CD69^{+} Tm across tissues. (**a** and **b**) Heatmaps of tissue-specific DMRs in promoters and introns associated with the cytokine (**a**) and chemokine (**b**) genes. Z-score-scaled methylation values are shown, with rows representing DMRs and columns representing individual samples unbiased grouped by tissue type(s). Genes associated with DMRs discussed in this study are labeled according to their positions on the heatmaps. Methylation values shown are scaled. (**c**) Quantification of methylation differences for DMR1 or Ref 1 of *CSF2* in CD69^{-} CD4 and CD8 Tm. Mean methylation (horizontal axis) is compared across distinct tissue CD69^{-} Tm populations and blood Tm. Box plots display averaged methylation levels, with the median, interquartile range, and whiskers showing data distribution. Significance is assessed with the Wilcoxon test: ****$p < 0.0001$, *** $p < 0.001$, ** $p < 0.01$, * $p < 0.05$.

Fig. S6

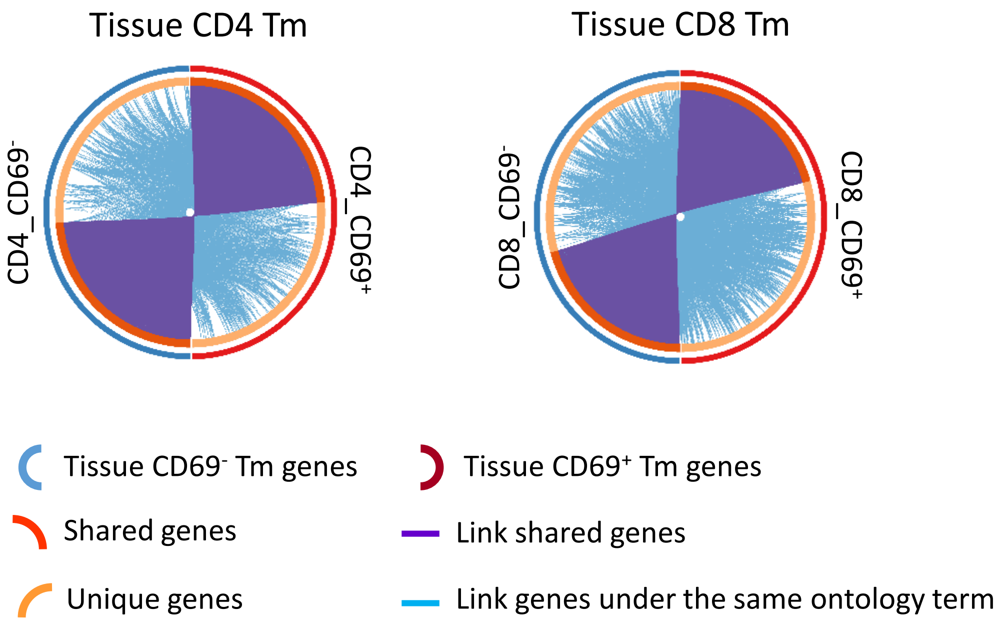

Fig. S6. (**Related to Fig. 3 and Table S4) Overlap of top 3000 gene lists associated with tissue-specific DMRs in CD69$^+$ and CD69$^-$ CD4 and CD8 Tm.** Pie charts illustrate the top 3000 genes associated with tissue-specific promoter/intron DMRs for each group, derived from pairwise comparisons of CD69$^+$ and CD69$^-$ populations in CD4 and CD8 Tm. In each chart, the outer red arc represents the gene list for the CD69$^+$ Tm, while the outer blue arc represents the gene list for the CD69$^-$ Tm. Shared genes between these CD69$^+$ and CD69$^-$ lists are highlighted in dark orange, connected by purple lines. Light blue lines link the genes that differ between the lists but fall under the same ontology term.

Fig. S7

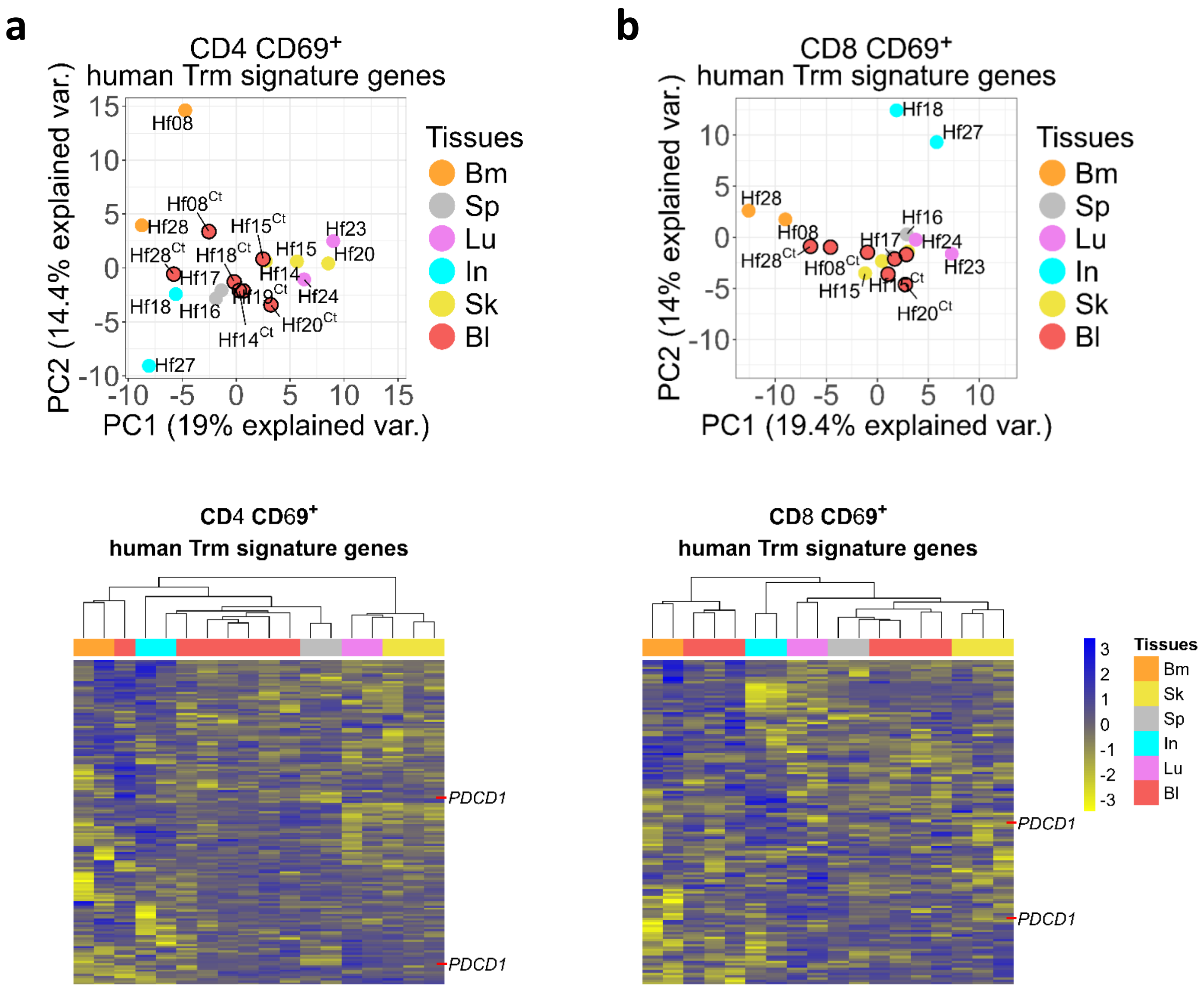
a
CD4 CD69+
human Trm signature genes
PC2 (14.4% explained var.)
PC1 (19% explained var.)
Tissues
Bm
Sp
Lu
In
Sk
Bl
b
CD8 CD69+
human Trm signature genes
PC2 (14% explained var.)
PC1 (19.4% explained var.)
CD4 CD69+
human Trm signature genes
PDCD1
PDCD1
CD8 CD69+
human Trm signature genes
PDCD1
PDCD1
Tissues
Bm
Sk
Sp
In
Lu
Bl

c

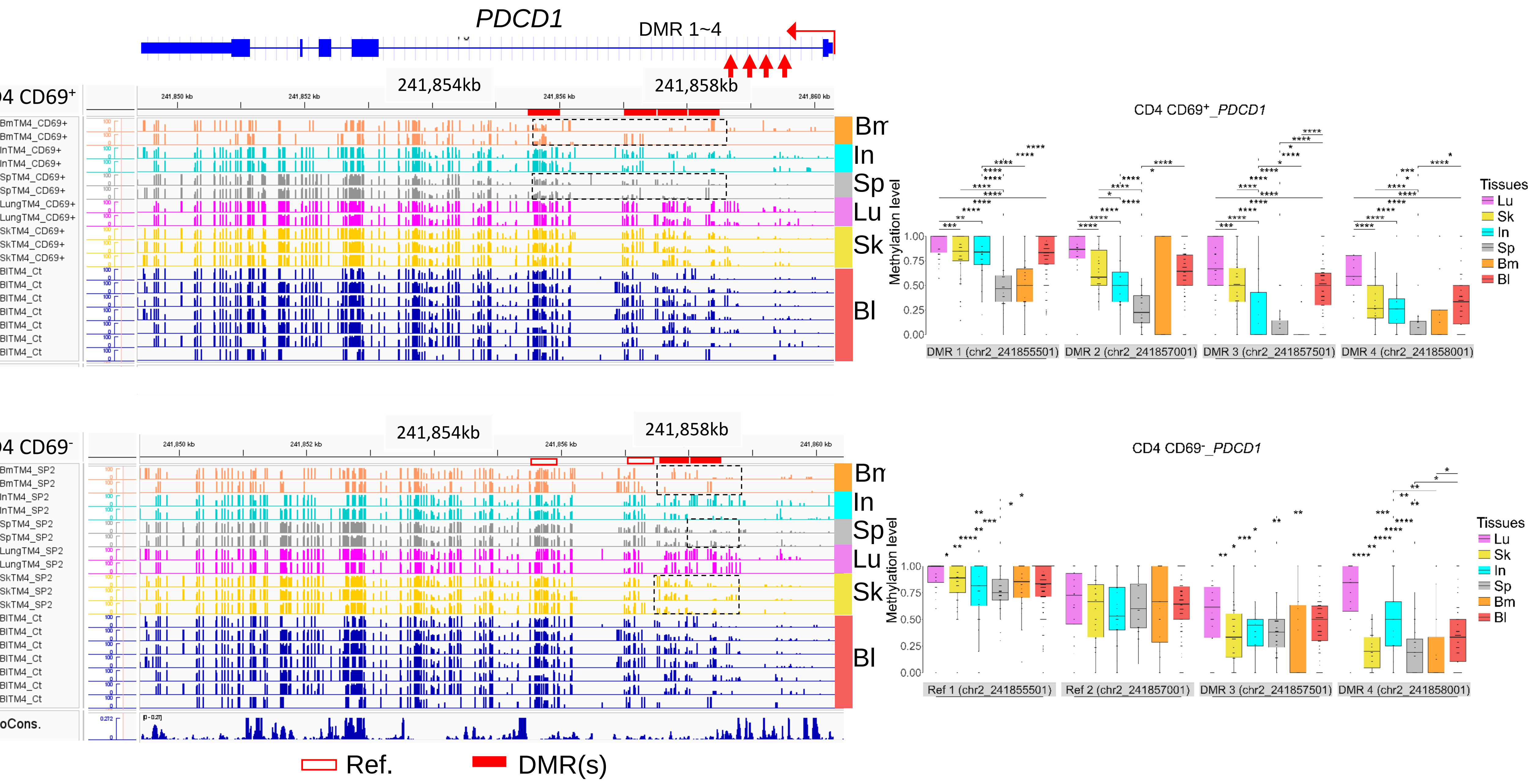
PDCD1
DMR 1~4
CD4 CD69+
241,854kb
241,858kb
241,850 kb
241,852 kb
241,856 kb
241,860 kb
Hf08_BmTM4_CD69+
Hf28_BmTM4_CD69+
Hf18_InTM4_CD69+
Hf27_InTM4_CD69+
Hf16_SpTM4_CD69+
Hf17_SpTM4_CD69+
Hf23_LungTM4_CD69+
Hf24_LungTM4_CD69+
Hf14_SkTM4_CD69+
Hf15_SkTM4_CD69+
Hf20_SkTM4_CD69+
Hf08_BlTM4_Ct
Hf14_BlTM4_Ct
Hf15_BlTM4_Ct
Hf18_BlTM4_Ct
Hf19_BlTM4_Ct
Hf20_BlTM4_Ct
Hf28_BlTM4_Ct
Bm
In
Sp
Lu
Sk
Bl
CD4 CD69+_PDCD1
Methylation level
1.00
0.75
0.50
0.25
0.00
DMR 1 (chr2_241855501)
DMR 2 (chr2_241857001)
DMR 3 (chr2_241857501)
DMR 4 (chr2_241858001)
Tissues
Lu
Sk
In
Sp
Bm
Bl
CD4 CD69-
Hf08_BmTM4_SP2
Hf28_BmTM4_SP2
Hf18_InTM4_SP2
Hf27_InTM4_SP2
Hf16_SpTM4_SP2
Hf17_SpTM4_SP2
Hf23_LungTM4_SP2
Hf24_LungTM4_SP2
Hf14_SkTM4_SP2
Hf15_SkTM4_SP2
Hf20_SkTM4_SP2
PhyloCons.
CD4 CD69-_PDCD1
Ref 1 (chr2_241855501)
Ref 2 (chr2_241857001)
Ref.
DMR(s)

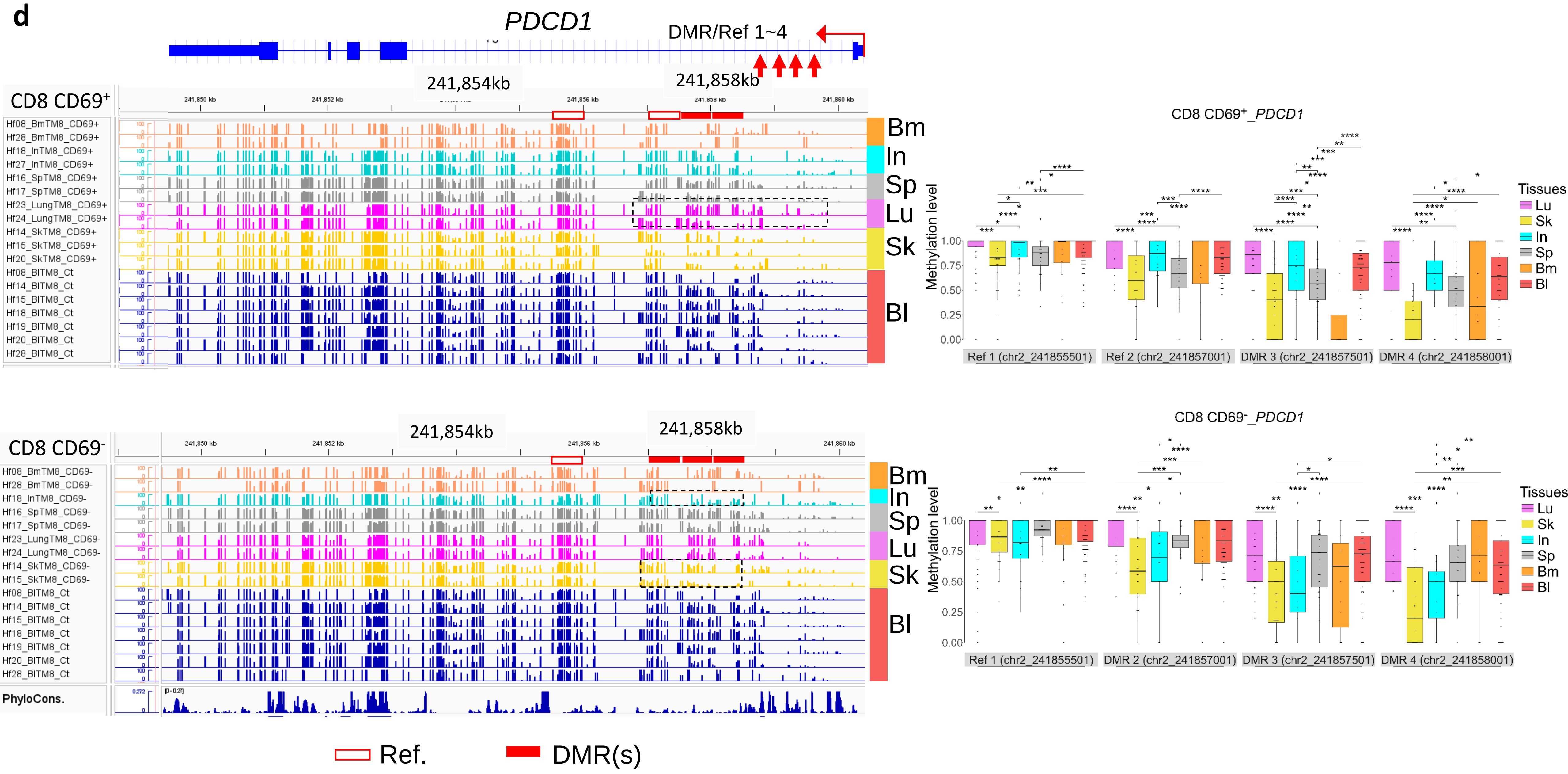

d
PDCD1
DMR/Ref 1~4
241,854kb
241,858kb
CD8 CD69+
Hf08_BmTM8_CD69+
Hf28_BmTM8_CD69+
Hf18_InTM8_CD69+
Hf27_InTM8_CD69+
Hf16_SpTM8_CD69+
Hf17_SpTM8_CD69+
Hf23_LungTM8_CD69+
Hf24_LungTM8_CD69+
Hf14_SkTM8_CD69+
Hf15_SkTM8_CD69+
Hf20_SkTM8_CD69+
Hf08_BlTM8_Ct
Hf14_BlTM8_Ct
Hf15_BlTM8_Ct
Hf18_BlTM8_Ct
Hf19_BlTM8_Ct
Hf20_BlTM8_Ct
Hf28_BlTM8_Ct
Bm
In
Sp
Lu
Sk
Bl
CD8 CD69+_PDCD1
Methylation level
Ref 1 (chr2_241855501)
Ref 2 (chr2_241857001)
DMR 3 (chr2_241857501)
DMR 4 (chr2_241858001)
Tissues
Lu
Sk
In
Sp
Bm
Bl
CD8 CD69-
Hf08_BmTM8_CD69-
Hf28_BmTM8_CD69-
Hf18_InTM8_CD69-
Hf16_SpTM8_CD69-
Hf17_SpTM8_CD69-
Hf23_LungTM8_CD69-
Hf24_LungTM8_CD69-
Hf14_SkTM8_CD69-
Hf15_SkTM8_CD69-
PhyloCons.
CD8 CD69-_PDCD1
Ref 1 (chr2_241855501)
DMR 2 (chr2_241857001)
DMR 3 (chr2_241857501)
DMR 4 (chr2_241858001)
Ref.
DMR(s)

e

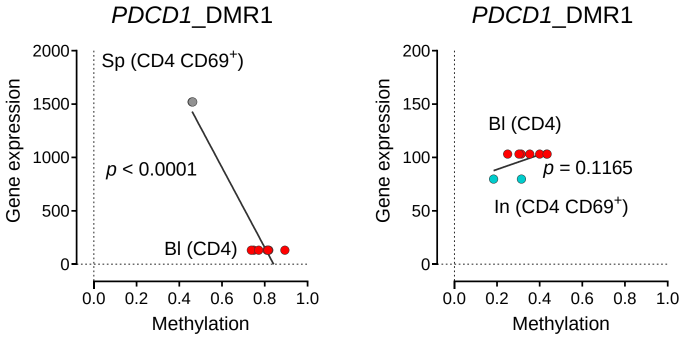

Fig. S7 (Related to Table S5) Differential methylation of core signature genes in Trm populations compared to blood Tm. (**a** and **b**) PCA and heatmap display differentially methylation of 31 core signature genes, previously identified as Trm markers, comparing tissue $CD69^{+}$ Tm to blood Tm in CD4 (**a**) and CD8 (**b**) T cell lineages. Data are based on pairwise comparison between tissue and blood Tm populations. (**c** and **d**) IGV visualization and quantification of DMRs and corresponding reference tiles for the *PDCD1* in both $CD69^{+}$ and $CD69^{-}$ CD4 (**c**) and CD8 (**d**) Tm. Quantification of DNA methylation quantification levels (horizontal axis) at the indicated DMRs or corresponding position tiles (labeled 'Ref') for in panels c and d. Mean methylation is compared across distinct tissue ($CD69^{+}$ and $CD69^{-}$) Tm populations and blood Tm. (**e**) Correlation analysis between DMRs and their corresponding gene expression levels between indicated CD4 $CD69^{+}$ tissue Tm and blood cells, using simple linear regression in GraphPad Prism. The line of best fit is shown, with *p*-values indicating significance. Gene expression levels were calculated from non-log-transformed values derived from different platforms: linear-scale microarray intensity, DESeq2-normalized counts, or CPM, as detailed in the Methods. Key symbols and methodologies are as described in Fig. S5.

Fig. S8

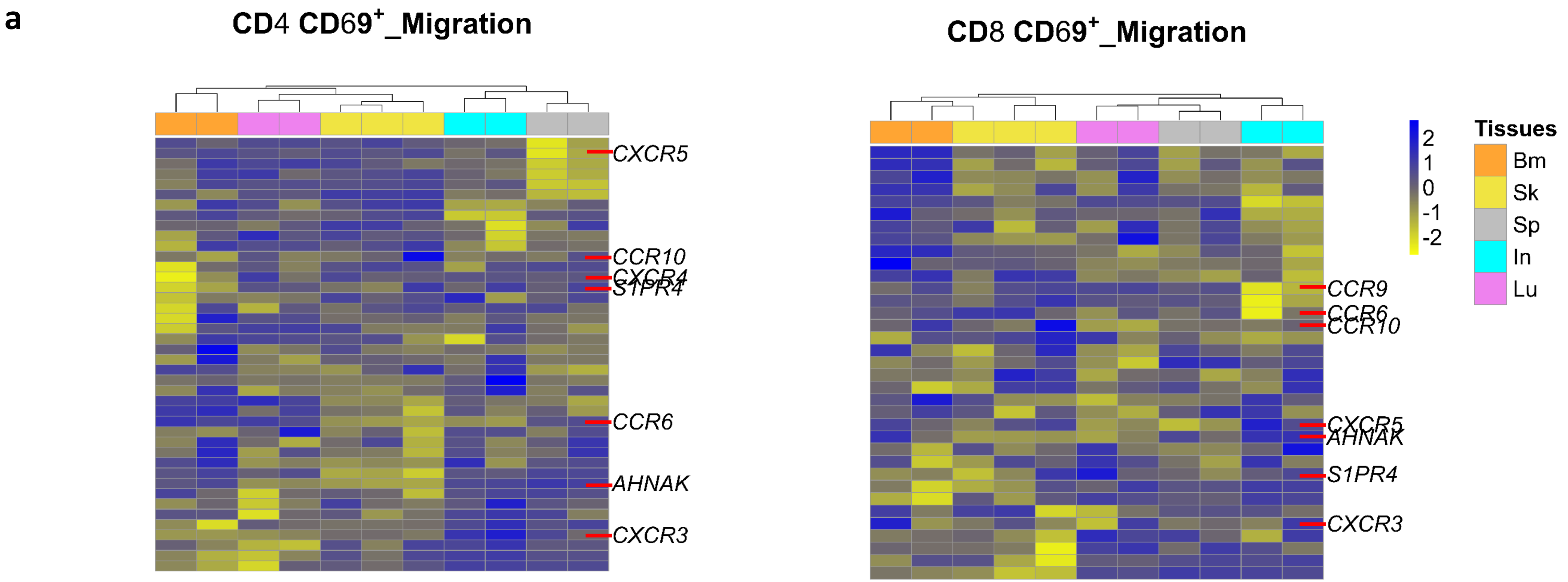
a
CD4 CD69+_Migration
CXCR5
CCR10
CXCR4
S1PR4
CCR6
AHNAK
CXCR3
CD8 CD69+_Migration
CCR9
CCR6
CCR10
CXCR5
AHNAK
S1PR4
CXCR3
2
1
0
-1
-2
Tissues
Bm
Sk
Sp
In
Lu

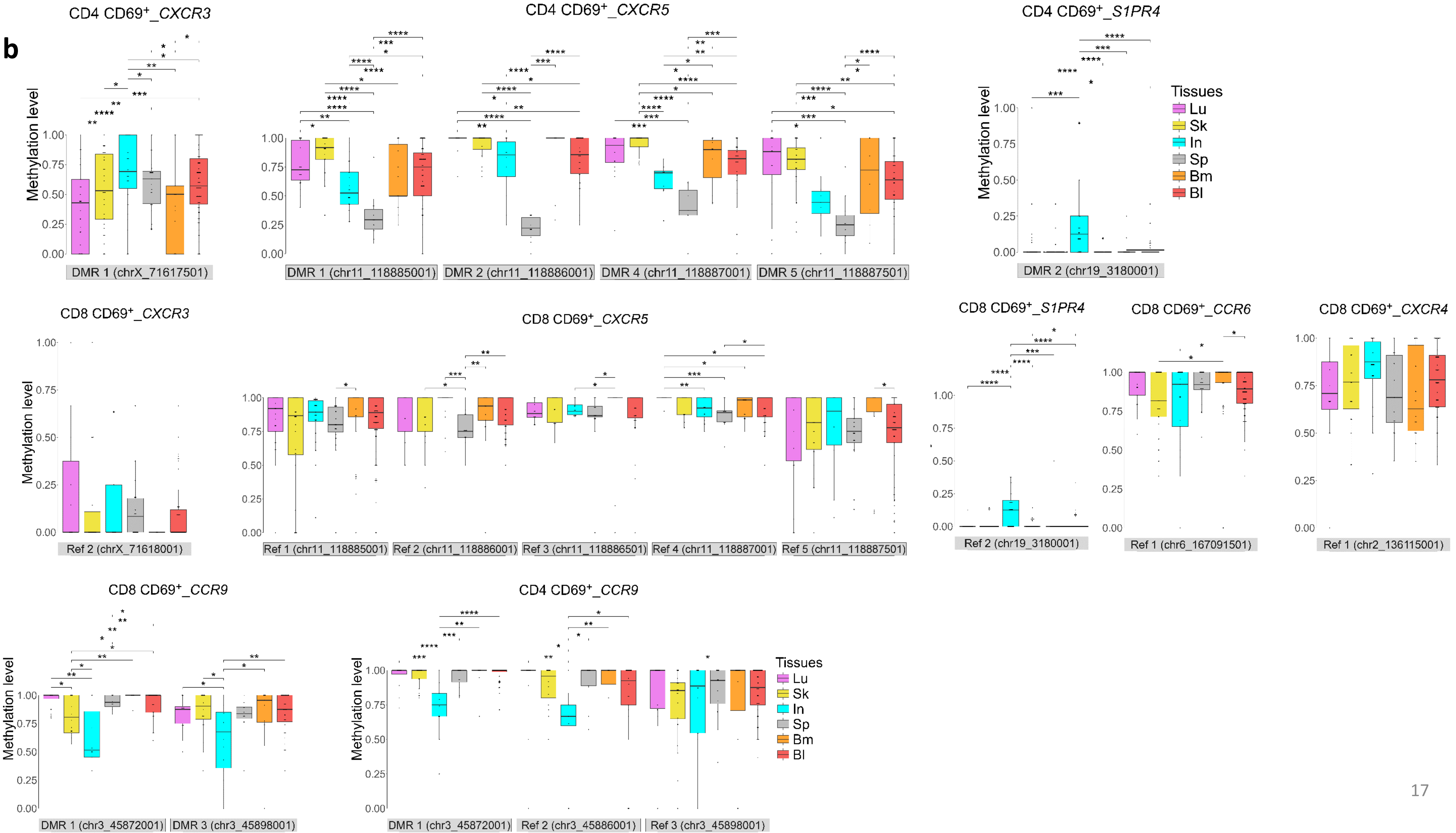
b
CD4 CD69⁺_CXCR3
DMR 1 (chrX_71617501)
CD4 CD69⁺_CXCR5
DMR 1 (chr11_118885001)
DMR 2 (chr11_118886001)
DMR 4 (chr11_118887001)
DMR 5 (chr11_118887501)
CD4 CD69⁺_S1PR4
DMR 2 (chr19_3180001)
Methylation level
Tissues
Lu
Sk
In
Sp
Bm
Bl
CD8 CD69⁺_CXCR3
Ref 2 (chrX_71618001)
CD8 CD69⁺_CXCR5
Ref 1 (chr11_118885001)
Ref 2 (chr11_118886001)
Ref 3 (chr11_118886501)
Ref 4 (chr11_118887001)
Ref 5 (chr11_118887501)
CD8 CD69⁺_S1PR4
Ref 2 (chr19_3180001)
CD8 CD69⁺_CCR6
Ref 1 (chr6_167091501)
CD8 CD69⁺_CXCR4
Ref 1 (chr2_136115001)
CD8 CD69⁺_CCR9
DMR 1 (chr3_45872001)
DMR 3 (chr3_45898001)
CD4 CD69⁺_CCR9
DMR 1 (chr3_45872001)
Ref 2 (chr3_45886001)
Ref 3 (chr3_45898001)

c

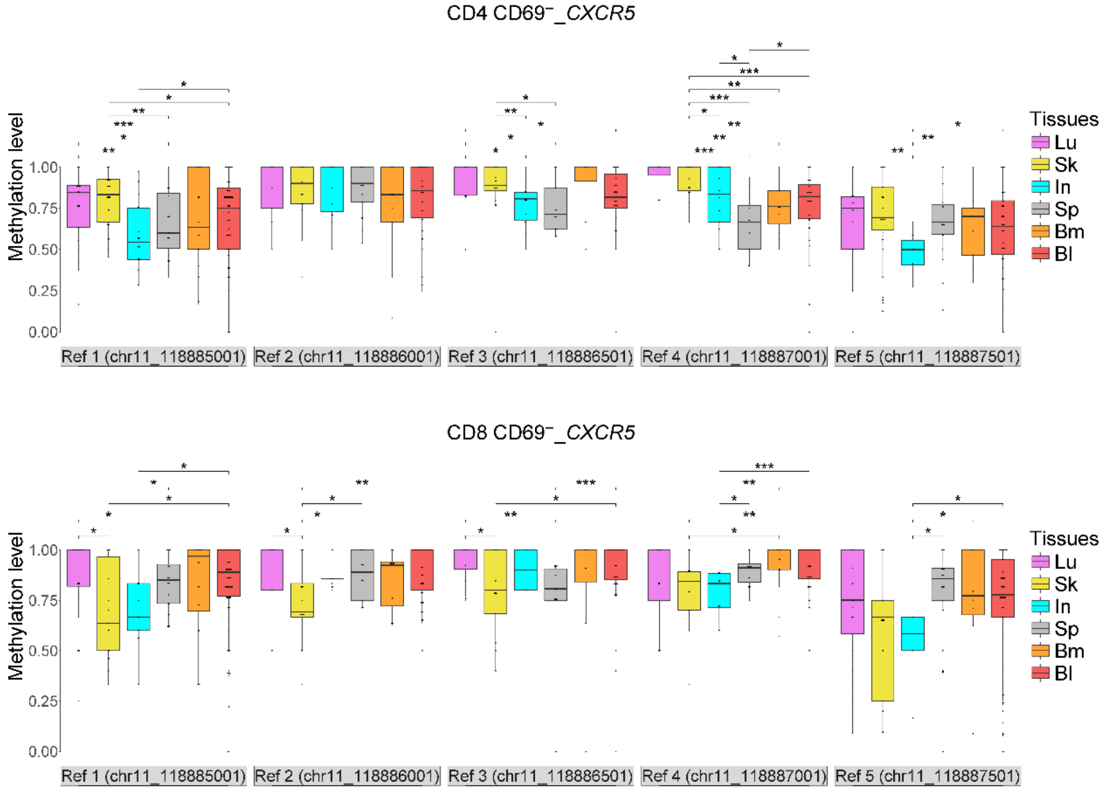

CD4 CD69⁻_CXCR5
Methylation level
1.00
0.75
0.50
0.25
0.00
Ref 1 (chr11_118885001)
Ref 2 (chr11_118886001)
Ref 3 (chr11_118886501)
Ref 4 (chr11_118887001)
Ref 5 (chr11_118887501)
Tissues
Lu
Sk
In
Sp
Bm
Bl
CD8 CD69⁻_CXCR5
Methylation level
1.00
0.75
0.50
0.25
0.00
Ref 1 (chr11_118885001)
Ref 2 (chr11_118886001)
Ref 3 (chr11_118886501)
Ref 4 (chr11_118887001)
Ref 5 (chr11_118887501)
Tissues
Lu
Sk
In
Sp
Bm
Bl

d

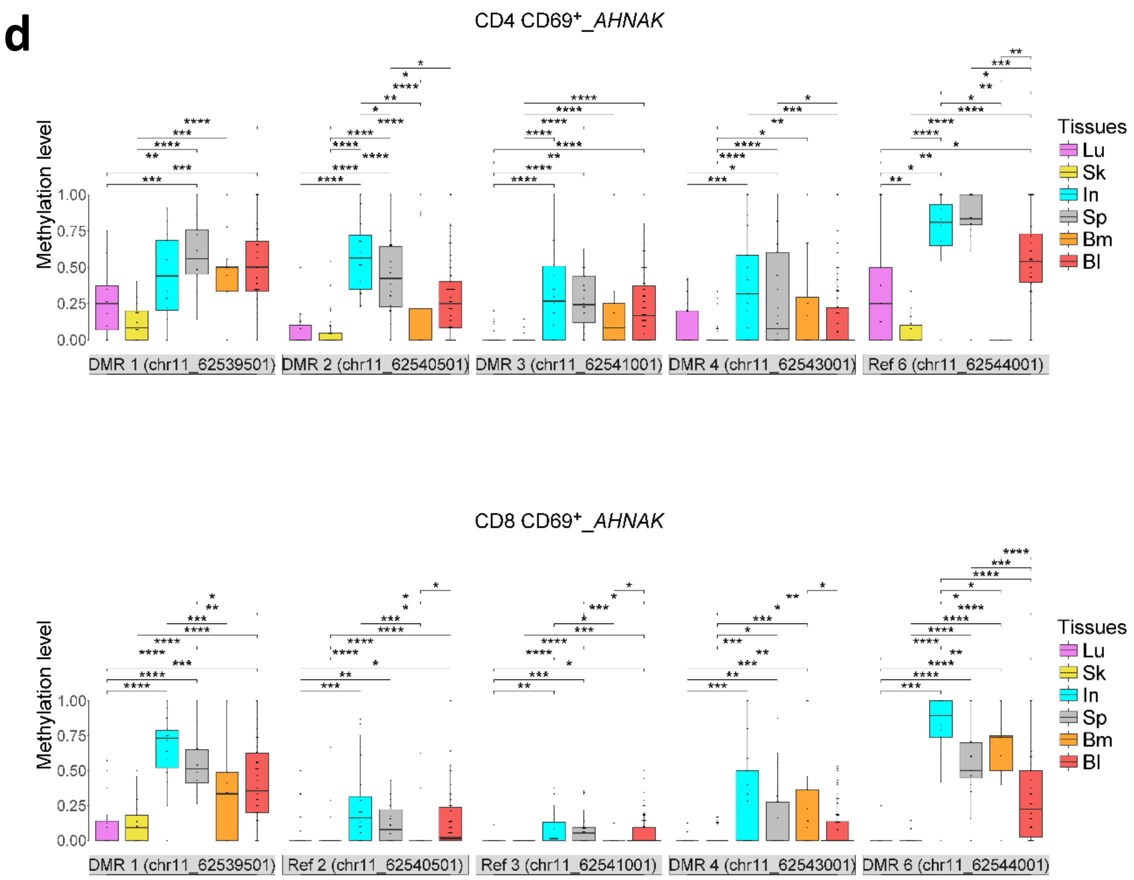

e

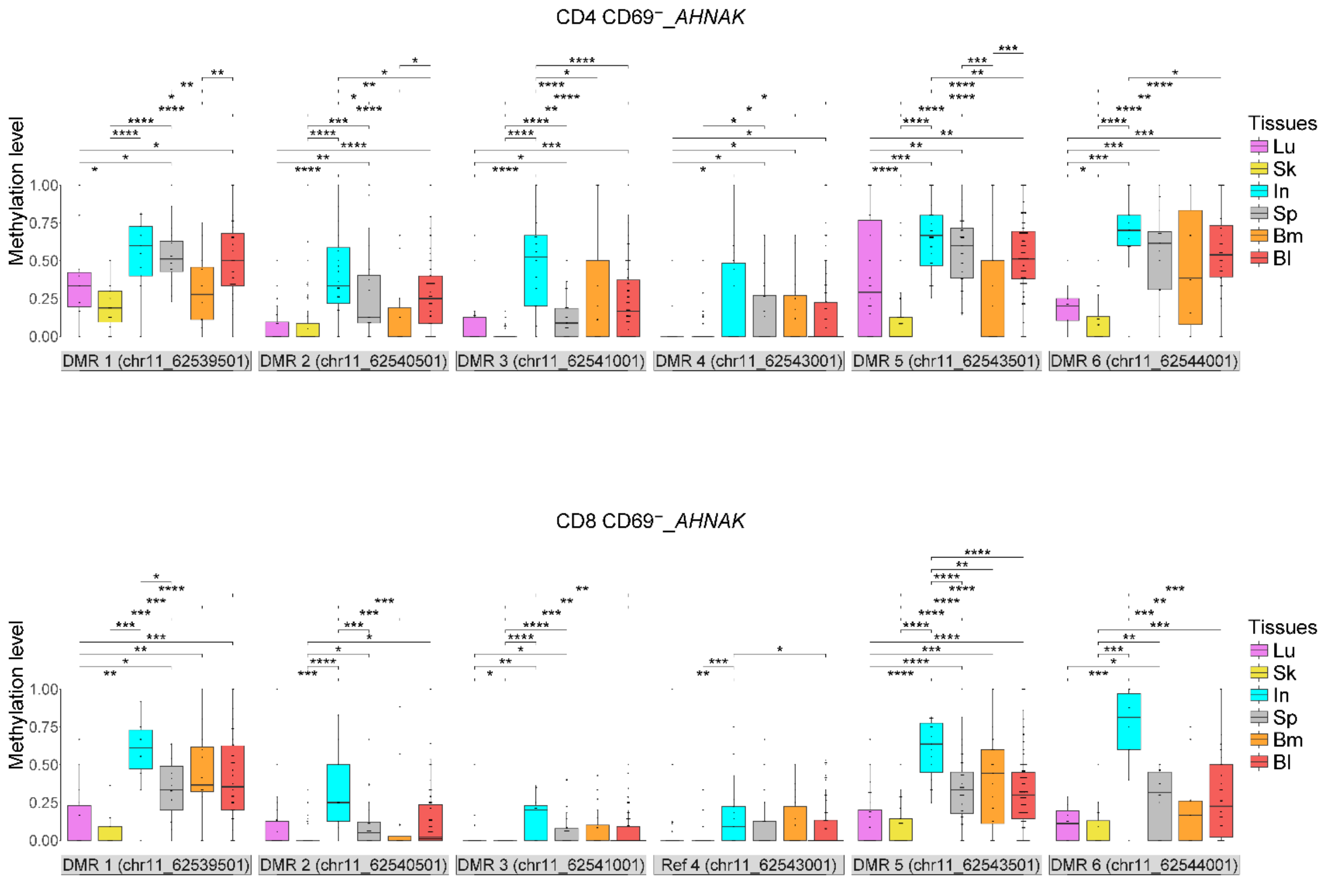

Fig. S8 (Related to Fig. 4; Table S6) Tissue-specific methylation of migration-linked genes between tissue and blood Tm populations. (**a**) Heatmap of the tissue-specific DMRs in promoters and introns associated with chemokine receptor, *S1P*, and *AHNAK* in CD4 and CD8 CD69^{+} Tm across tissues. Genes associated with DMRs discussed in this study are labeled according to their positions on the heatmaps. (**b**-**e**) Quantification of methylation differences across tissue and blood Tm populations for indicated DMRs and reference tiles of indicated genes in CD69^{+} (**b** and **d**) and CD69^{-} (**c** and **d**) CD4 and CD8 Tm. Key symbols and methodologies are as described in Fig. S5.

Fig. S9

a

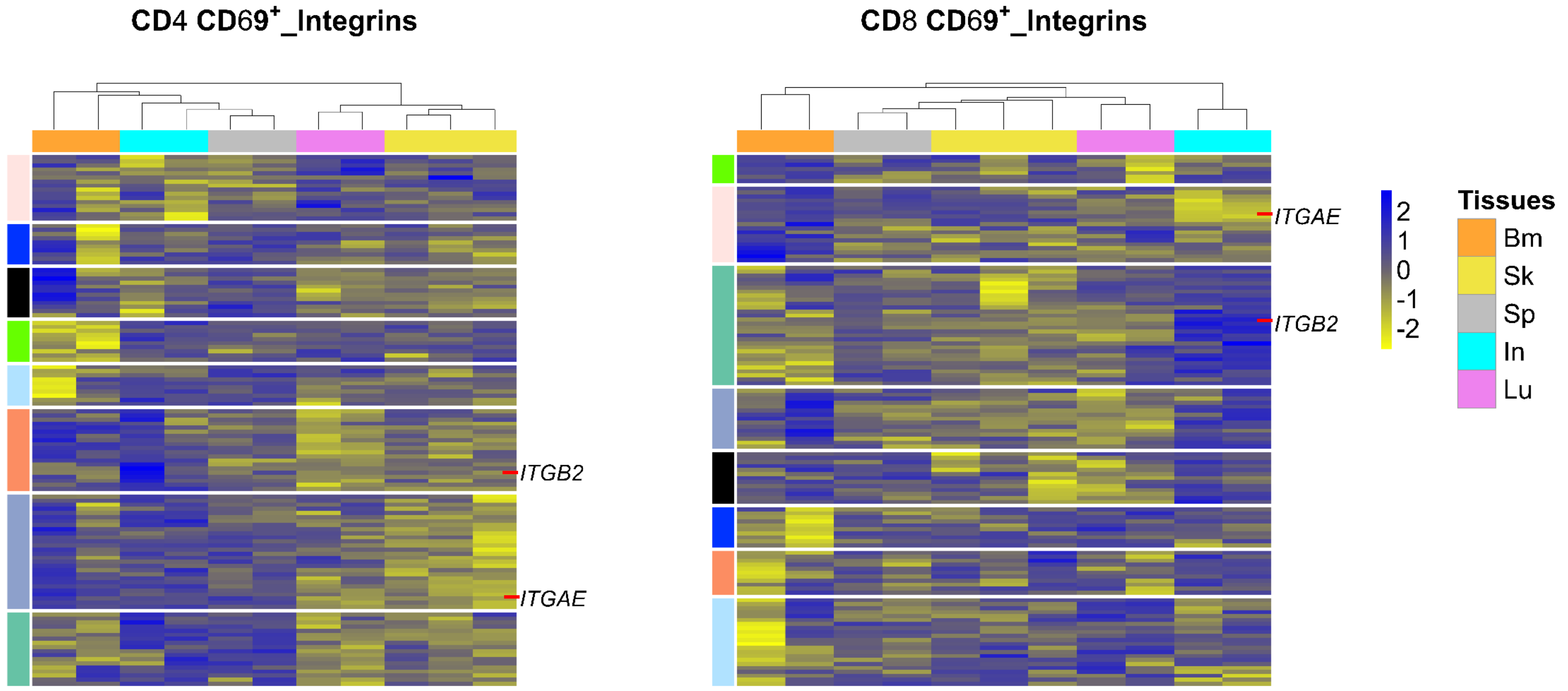
CD4 CD69+_Integrins
ITGB2
ITGAE
CD8 CD69+_Integrins
ITGAE
ITGB2
2
1
0
-1
-2
Tissues
Bm
Sk
Sp
In
Lu

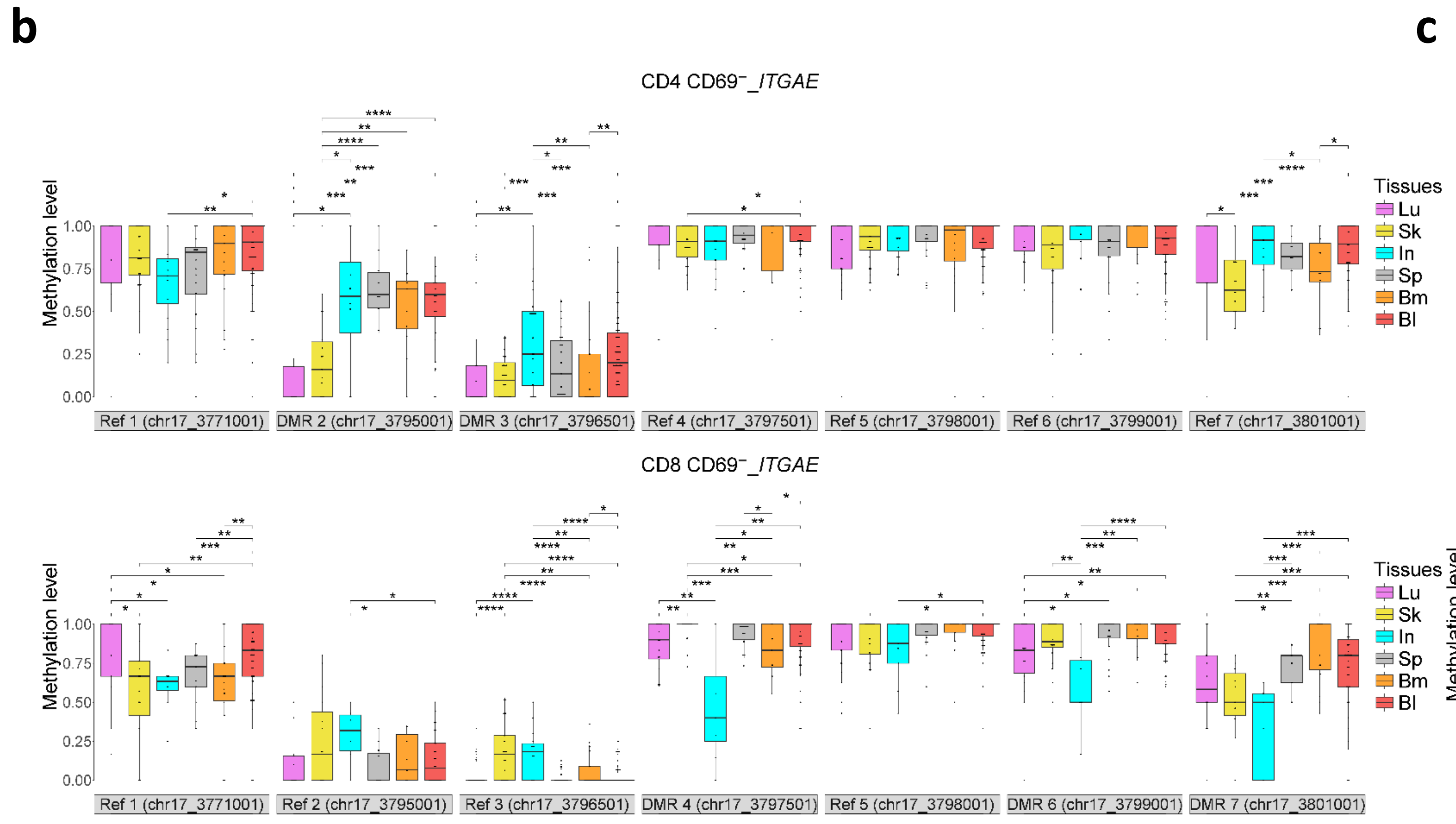

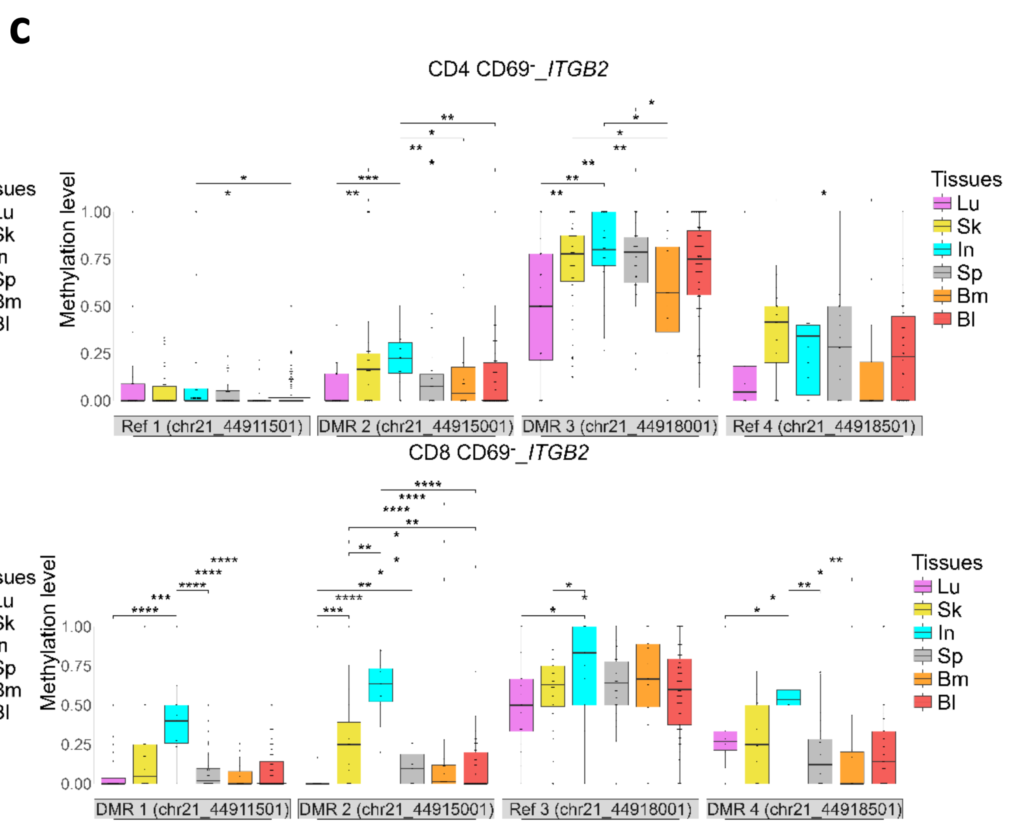

Fig. S9. (Related to Fig. 5 and Table S7) Tissue-specific methylation of integrin genes between tissue and blood Tm populations (**a**) Heatmap of the tissue-specific DMRs in promoters and introns associated with integrin genes in CD69^{+} CD4 and CD8 Tm across tissues. The genes of differential methylation are highlighted for visual comparison. Genes associated with DMRs discussed in this study are labeled according to their positions on the heatmaps. (**b** and **c**) Quantification of methylation differences across tissue and blood CD69^{-} CD4 and CD8 Tm populations for indicated DMRs and respective reference tiles of *ITGAE* (**b**) and *ITGB2* (**c**). Key symbols and methodologies are as described in Fig. S5.

Fig. S10

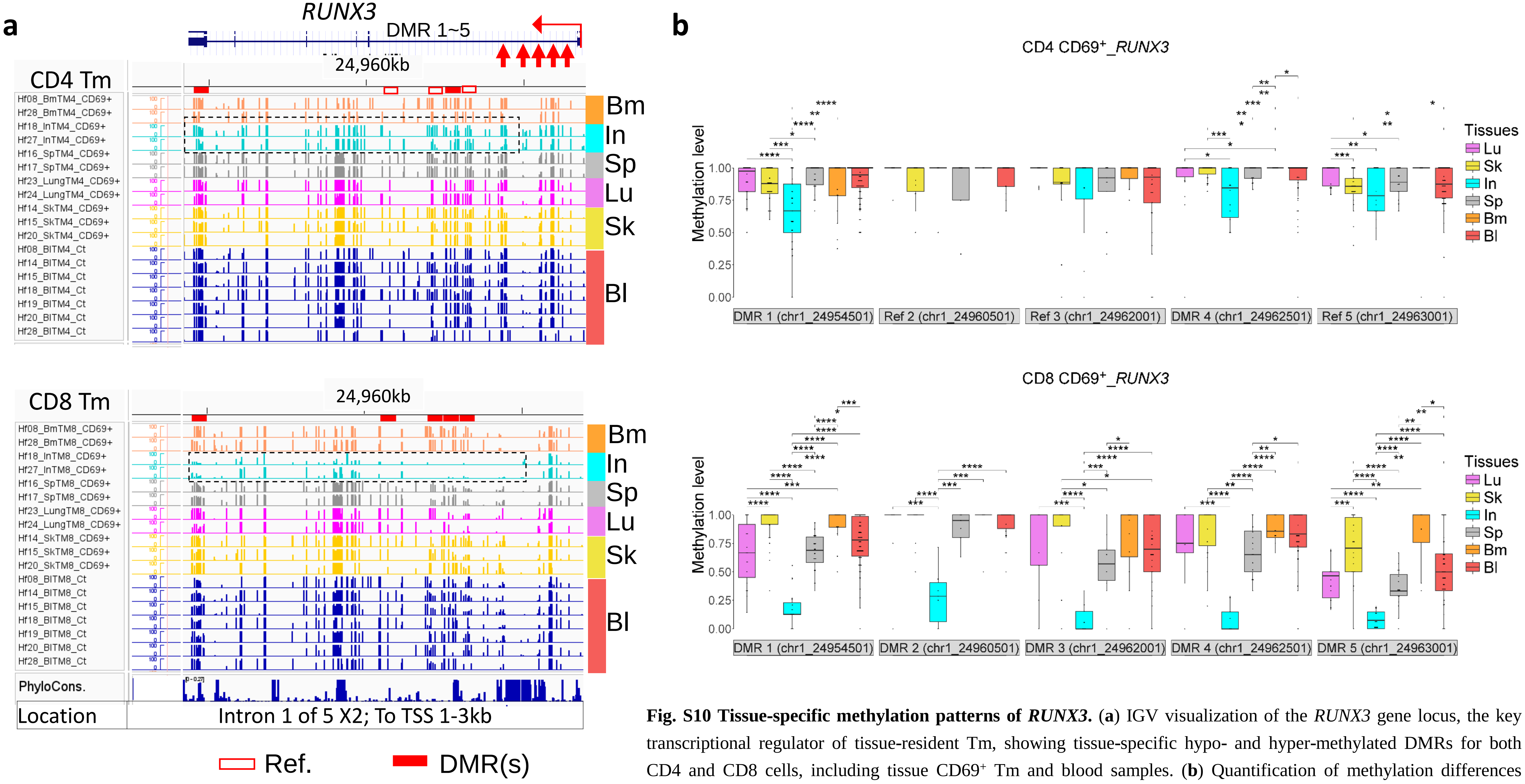

Fig. S10 Tissue-specific methylation patterns of *RUNX3*. (**a**) IGV visualization of the *RUNX3* gene locus, the key transcriptional regulator of tissue-resident Tm, showing tissue-specific hypo- and hyper-methylated DMRs for both CD4 and CD8 cells, including tissue CD69^{+} Tm and blood samples. (**b**) Quantification of methylation differences across tissue and blood Tm populations for indicated DMRs shown in **a**. Key symbols and methodologies are as described in Fig. S5.

Fig. S11

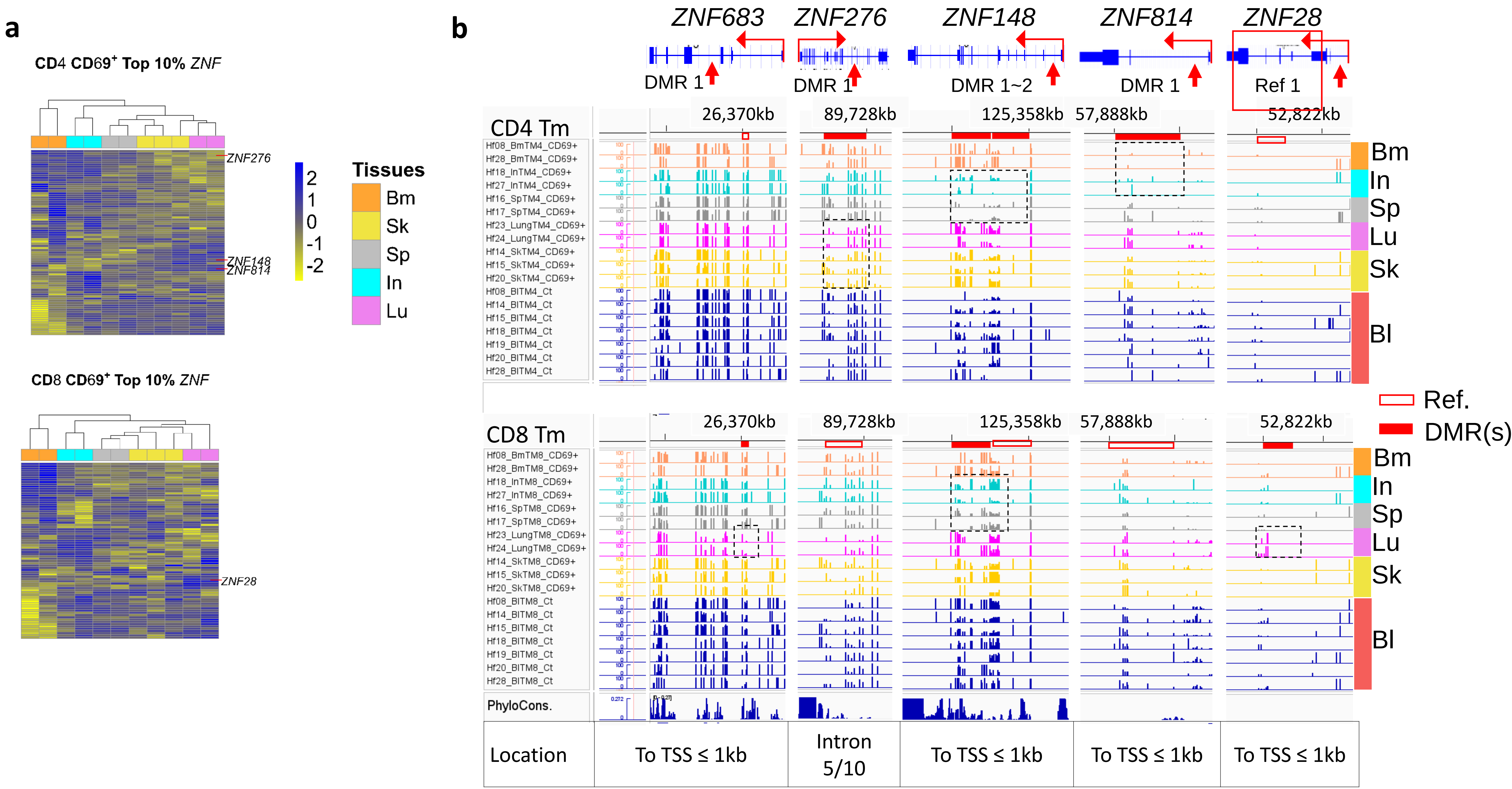
a
CD4 CD69+ Top 10% ZNF
ZNF276
ZNF148
ZNF814
CD8 CD69+ Top 10% ZNF
ZNF28
2
1
0
-1
-2
Tissues
Bm
Sk
Sp
In
Lu
b
ZNF683
ZNF276
ZNF148
ZNF814
ZNF28
DMR 1
DMR 1
DMR 1~2
DMR 1
Ref 1
CD4 Tm
26,370kb
89,728kb
125,358kb
57,888kb
52,822kb
CD8 Tm
PhyloCons.
Bm
In
Sp
Lu
Sk
Bl
Ref.
DMR(s)
Location
To TSS ≤ 1kb
Intron 5/10
To TSS ≤ 1kb
To TSS ≤ 1kb
To TSS ≤ 1kb

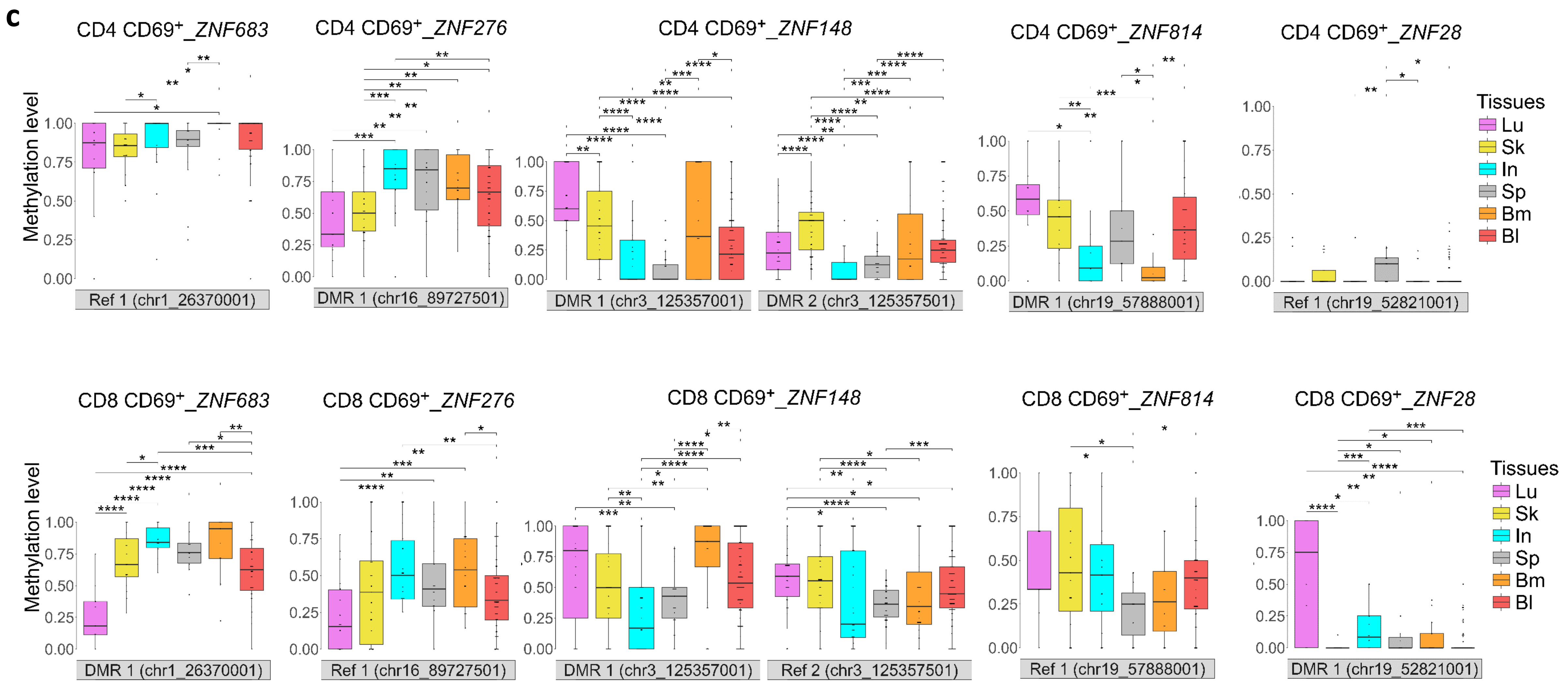

Fig. S11 Tissue-specific methylation landscapes of *ZNF* family members in CD69⁺ Tm of both CD4 and CD8 lineages. (**a**) Heatmap of the top 10% tissue-specific DMRs in promoters and introns associated with the *ZNF* genes. Genes associated with DMRs discussed in this study are labeled according to their positions on the heatmaps. (**b**) IGV visualization of indicated tissue-specific *ZNF* hypo- and hyper-DMRs for both CD4 and CD8 T cells, including tissue CD69⁺ and blood samples. The regions of differential methylation are highlighted for visual comparison. (**c**) Quantification of methylation differences for indicated DMRs or reference tiles across tissue and blood Tm populations. Key symbols and methodologies are as described in Fig. S5.

Fig. S12

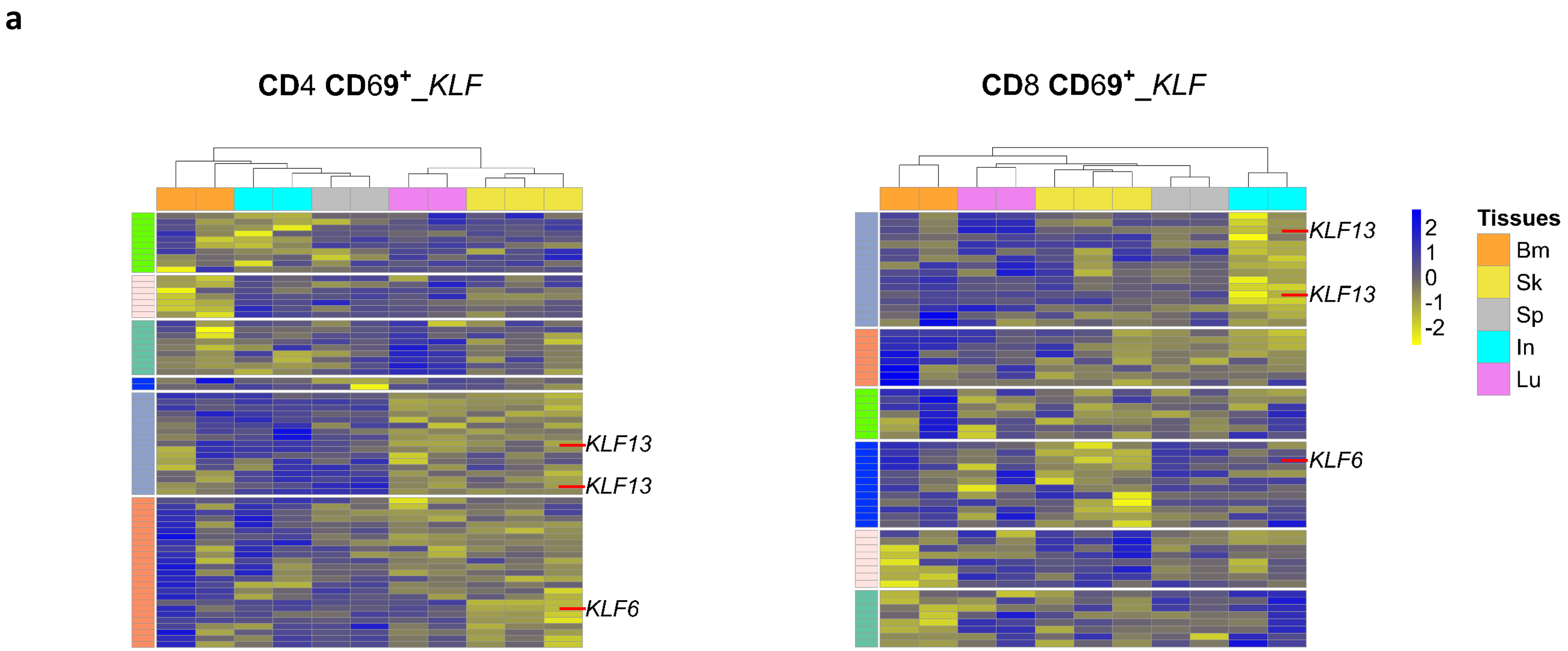
a
CD4 CD69+_KLF
KLF13
KLF13
KLF6
CD8 CD69+_KLF
KLF13
KLF13
KLF6
2
1
0
-1
-2
Tissues
Bm
Sk
Sp
In
Lu

b

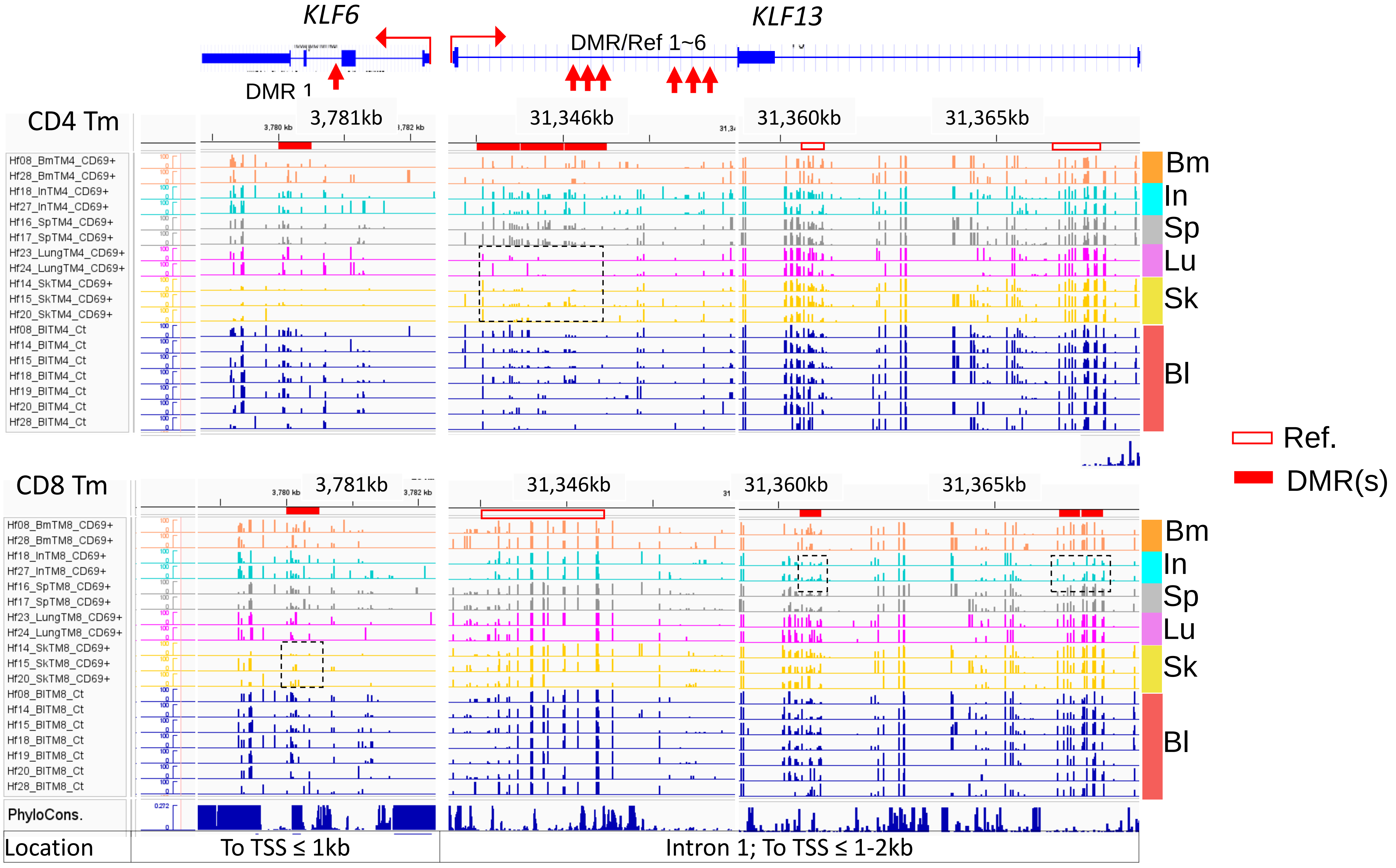
KLF6
KLF13
DMR/Ref 1~6
DMR 1
CD4 Tm
3,781kb
31,346kb
31,360kb
31,365kb
Hf08_BmTM4_CD69+
Hf28_BmTM4_CD69+
Hf18_InTM4_CD69+
Hf27_InTM4_CD69+
Hf16_SpTM4_CD69+
Hf17_SpTM4_CD69+
Hf23_LungTM4_CD69+
Hf24_LungTM4_CD69+
Hf14_SkTM4_CD69+
Hf15_SkTM4_CD69+
Hf20_SkTM4_CD69+
Hf08_BlTM4_Ct
Hf14_BlTM4_Ct
Hf15_BlTM4_Ct
Hf18_BlTM4_Ct
Hf19_BlTM4_Ct
Hf20_BlTM4_Ct
Hf28_BlTM4_Ct
Bm
In
Sp
Lu
Sk
Bl
Ref.
DMR(s)
CD8 Tm
3,781kb
31,346kb
31,360kb
31,365kb
Hf08_BmTM8_CD69+
Hf28_BmTM8_CD69+
Hf18_InTM8_CD69+
Hf27_InTM8_CD69+
Hf16_SpTM8_CD69+
Hf17_SpTM8_CD69+
Hf23_LungTM8_CD69+
Hf24_LungTM8_CD69+
Hf14_SkTM8_CD69+
Hf15_SkTM8_CD69+
Hf20_SkTM8_CD69+
Hf08_BlTM8_Ct
Hf14_BlTM8_Ct
Hf15_BlTM8_Ct
Hf18_BlTM8_Ct
Hf19_BlTM8_Ct
Hf20_BlTM8_Ct
Hf28_BlTM8_Ct
Bm
In
Sp
Lu
Sk
Bl
PhyloCons.
Location
To TSS ≤ 1kb
Intron 1; To TSS ≤ 1-2kb

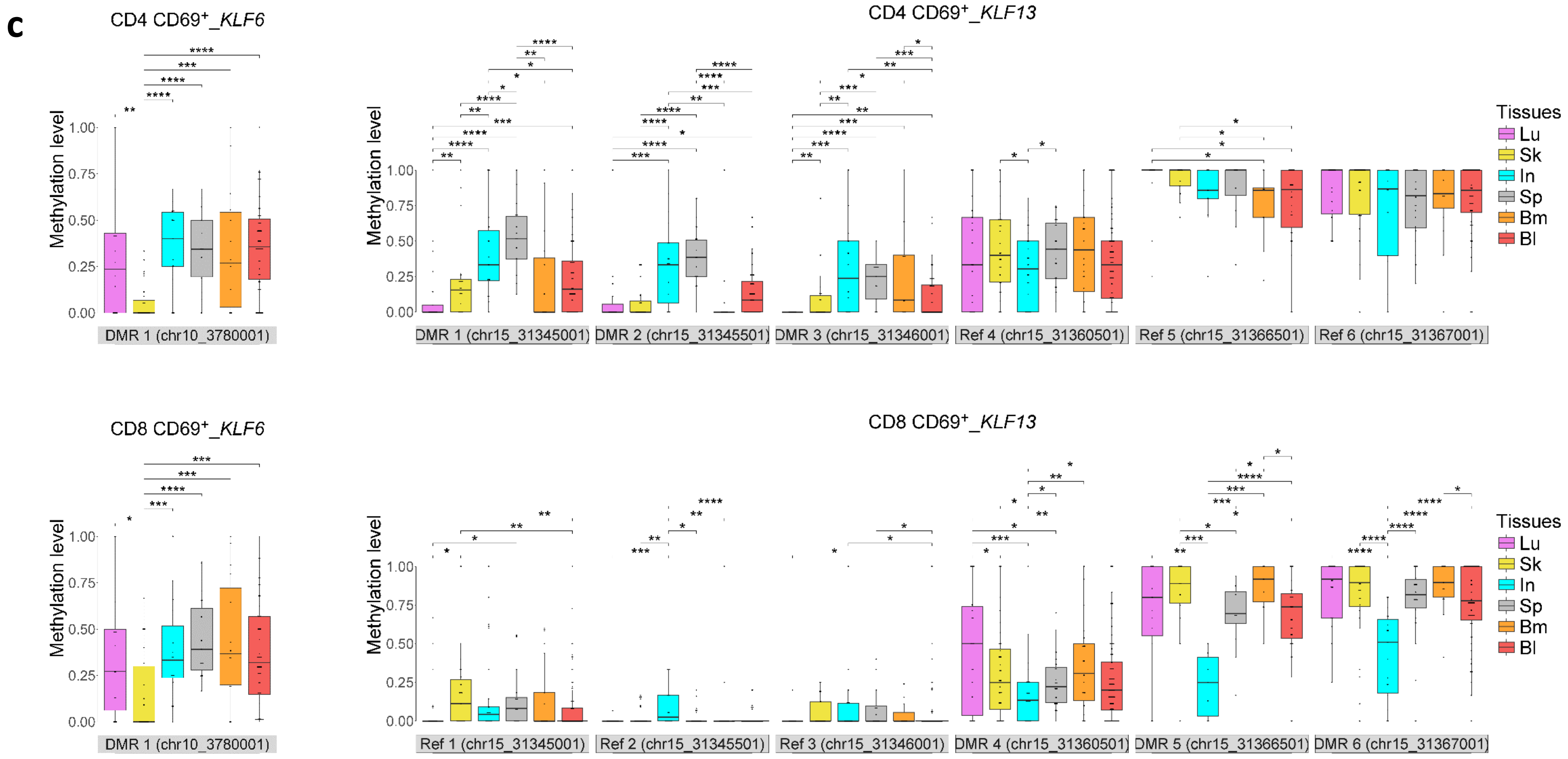
c
CD4 CD69+_KLF6
CD4 CD69+_KLF13
CD8 CD69+_KLF6
CD8 CD69+_KLF13
Methylation level
DMR 1 (chr10_3780001)
DMR 1 (chr15_31345001)
DMR 2 (chr15_31345501)
DMR 3 (chr15_31346001)
Ref 4 (chr15_31360501)
Ref 5 (chr15_31366501)
Ref 6 (chr15_31367001)
Ref 1 (chr15_31345001)
Ref 2 (chr15_31345501)
Ref 3 (chr15_31346001)
DMR 4 (chr15_31360501)
DMR 5 (chr15_31366501)
DMR 6 (chr15_31367001)
Tissues
Lu
Sk
In
Sp
Bm
Bl

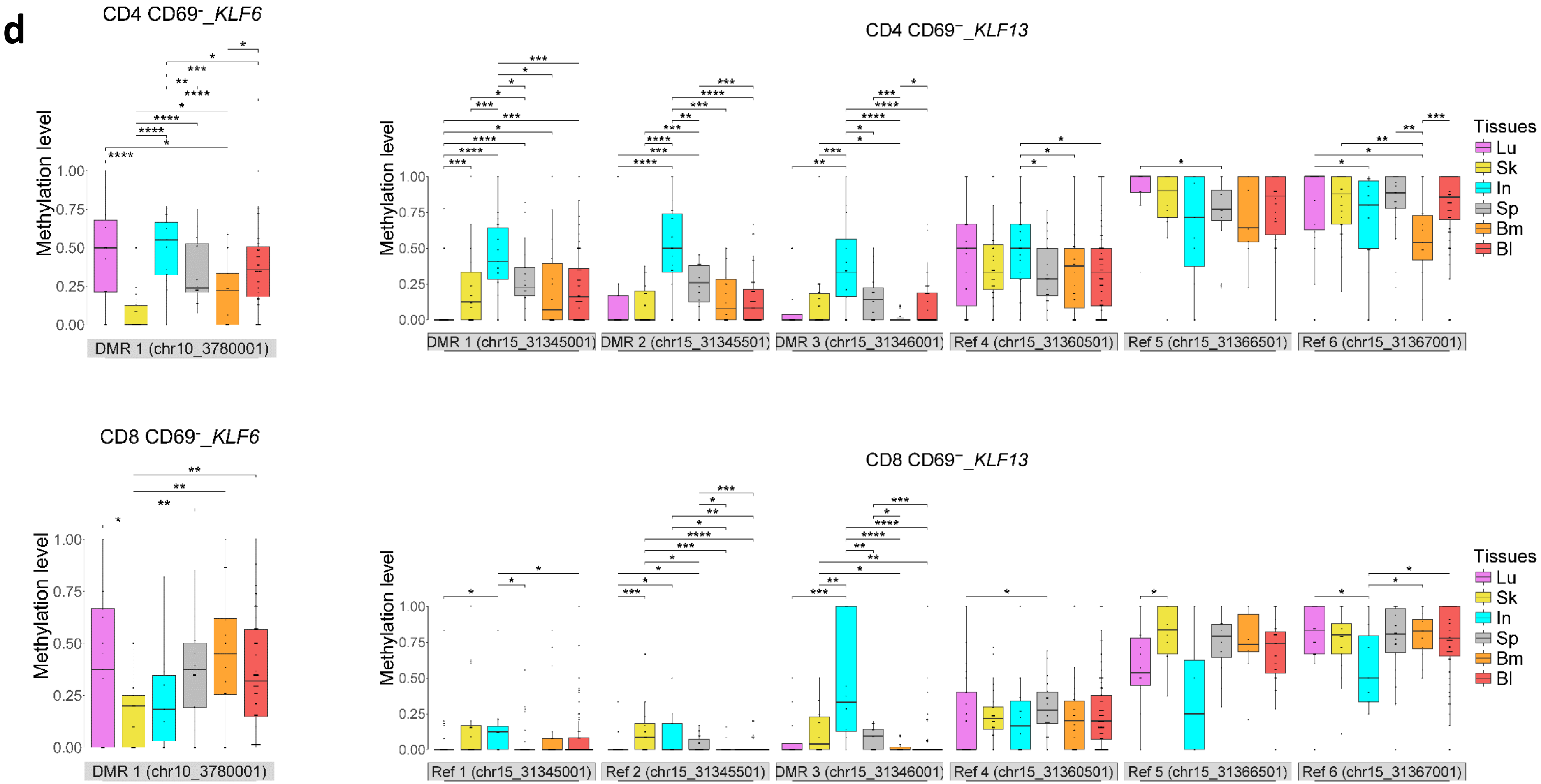

Fig. S12 Dynamic tissue-specific methylation profiles of *KLF* family genes in CD69$^+$ Tm of both CD4 and CD8 lineages. (**a**) Heatmaps of the tissue-specific DMRs in promoters and introns associated with *KLF* genes. Genes associated with DMRs discussed in this study are labeled according to their positions on the heatmaps. (**b-d**) IGV visualization (**b**) of tissue-specific *KLF6* and *KLF13* gene hypo- and hyper-DMRs and quantification of their DNA methylation levels for both CD69$^+$ (**c**) and CD69$^-$ (**d**) CD4 and CD8 Tm, including tissue and blood samples. Mean methylation is compared across distinct in tissue and blood Tm populations. The regions of differential methylation are highlighted for visual comparison. Multiple DMRs of the same gene may be present. Key to symbols as described in Fig. S5.
