# Supplementary material for: Methylomes of human CD4 and CD8 memory T lymphocytes reveal tissue-specific epigenetic signatures for maintenance and recall function
Source: Immun Inflamm. 2025 Oct 1;1(1):13. doi: 10.1007/s44466-025-00009-x (PMC12623507; doi:10.1007/s44466-025-00009-x)
Supplement: Supplementary file 2 — Supplementary Material 2: Fig. S1-S4, related to Fig. 1 and Tables S1 and S2. Fig. S5, related to Fig. 2 and Table S3. Fig. S6, related to Fig. 3 and Table S4. Fig. S7, related to Table S5. Fig. S8, related to Fig. 4 and Table S6. Fig. S9, related to Fig. 5 and Table S7. Fig. S11, related to Table S8. Fig. S12, related to Table S9. [file 44466_2025_9_MOESM2_ESM.zip › SI_Figure_Table_Legends_R2.pdf]

**Supplementary Information**

**Fig. S1** (**Related to Fig. 1s) Selective expression of CD69 by human ex vivo tissue-derived CD4 and CD8 Tm. (a)** Representative flow cytometry histograms showing CD69 expression by human ex vivo tissue-derived Tm (lines) versus blood counterparts or FMO controls (grey areas). The number of positive cells (% parent population) are included in each plot. (**b)** Average of $CD69^+$ cells among CD4 and CD8 Tm across tissue and blood samples. Each dot represents an individual sample. (**c)** Exemplar flow cytometry histograms depicting proliferation marker KI-67, early activation markers CD25 and CD154, and late activation markers such as HLA-DR on indicated tissue $CD45RA^+$ naive and $CD69^+$ and $CD69^-$ CD4 Tm subsets.

**Fig. S2 (Related to Fig. 1) Isolation of Tm subsets from multiple human tissues and blood.** (**a** and **b**) Representative flow cytometry gating strategy (**a**) and purity check plots (**b**) depicting CD69 versus CD25 expression on CD4 Tm before and after isolation of $CD69^+$ and $CD69^-$ cell subsets, both of which are $CD25^-$. The number of positive and/or negative cells (% of parent populations) is included in each plot. Sequential inclusive gates were applied to select $CD45^+$, lymphocytes, and live cells. A CD69 vs. DUMP marker exclusion gate was used to remove only events coexpressing CD69 and DUMP markers (diagonal population: $CD3^-$, $CD19^+$, and $CD14^+$). Events within the $CD69^+DUMP^-$, $CD69^-DUMP^+$, or $CD69^-DUMP^-$ quadrants were retained for downstream analysis.

**Fig. S3 (Related to Fig. 1c) (a and b) Quantification of methylation differences between Tm populations in tissues and/or blood.** The percentage of DMRs among all qualified tiles for each indicated pairwise comparisons between $CD69^+$ and $CD69^-$ populations in CD4 (**a**) and CD8 (**b**) Tm lineages. (**c**) Interindividual differences in DNA methylation (of those in panel **c**) across Tm subsets. Variance in DNA methylation (corresponding to the data shown in panel **c**) is displayed for each Tm subset per donor. '+' and '−' refer to CD69 expression, indicating tissue-$CD69^+$ and $CD69^−$ and blood $CD69^−$ Tm. (**d**) PCA plots of $CD69^-$ CD4 and CD8 tissue Tm populations, including blood counterparts (labelled as Ct; >98% $CD69^-$) for comparison. Each PCA was generated from DMRs derived exclusively from pairwise comparisons as in **a** and **b**. MANOVA results (F- and P-values) for tissue groups are shown on each plot. Replicates from each tissue types are indicated.

**Fig. S4** (**Related to Fig. 1e) Blood-derived Tm data related to tissue-specific methylome patterns of $CD69^+$ and $CD69^-$ Tm populations.** (**a** and **b**) Methylation patterns of blood-derived CD4 (**a**) and CD8 (**b**) Tm populations based on the values of tissue-specific DMRs described in Fig. 1e. Each row represents a DMR and each column a sample. Colors indicate relative DNA methylation levels (Z-score scaled across samples). White lines separate clusters of DMRs identified by hierarchical clustering.

**Fig. S5** (**Related to Fig. 2; Table S3) Epigenetic imprinting of silent Th/Tc effector genes in $CD69^+$ Tm across tissues** (**a** and **b**) Heatmaps of tissue-specific DMRs in promoters and introns associated with the cytokine (**a**) and chemokine (**b**) genes. Genes associated with DMRs discussed in this study are

labeled according to their positions on the heatmaps. (**c)** Quantification of methylation differences for DMR1 of CSF2 in CD69$^-$ CD4 and CD8 Tm. Mean methylation values (horizontal axis) are plotted for CD69$^-$ Tm from various tissue types compared to blood Tm. Box plots display averaged methylation levels, with the median, interquartile range, and whiskers showing data distribution. Significance is assessed with the Wilcoxon test: ****$p < 0.0001$, *** $p < 0.001$, ** $p < 0.01$, * $p < 0.05$.

**Fig. S6 (Related to Fig. 3; Table S4) Overlap of top 3000 gene lists associated with tissue-specific DMRs in CD69$^+$ and CD69$^-$ CD4 and CD8 Tm.** Pie charts illustrate the top 3000 genes associated with tissue-specific promoter/intron DMRs for each group, derived from pairwise comparisons of CD69$^+$ and CD69$^-$ populations in CD4 and CD8 Tm. In each chart, the outer red arc represents the gene list for the CD69$^+$ Tm, while the outer blue arc represents the gene list for the CD69$^-$ Tm. Shared genes between these CD69$^+$ and CD69$^-$ lists are highlighted in dark orange, connected by purple lines. Light blue lines link the genes that differ between the lists but fall under the same ontology term.

**Fig. S7 Differential methylation of core signature genes in Trm populations compared to blood Tm.** (**a** and **b**), PCA and heatmap display differentially methylation of 31 core signature genes, previously identified as Trm markers, comparing tissue CD69$^+$ Tm to blood Tm in CD4 (**a**) and CD8 (**b**) T cell lineages. Data are based on pairwise comparison between tissue and blood Tm populations. (**c** and **d**) IGV visualization and quantification of DMRs and corresponding reference tiles for the PDCD-1 in both CD69$^+$ and CD69$^-$ CD4 (**c**) and CD8 (**d**) Tm. Quantification plots show methylation differences for indicated DMRs or corresponding position tiles (framed and labeled as 'Ref') highlighted in panels **a** and **b**. Mean methylation values (horizontal axis) are plotted for CD69$^+$ or CD69$^-$ from indicated tissue types and blood Tm. (**e**) Correlation analysis between DMRs and their corresponding gene expression levels between indicated CD4 CD69$^+$ tissue Tm and blood cells, using simple linear regression in GraphPad Prism. The line of best fit is shown, with p-values indicating significance. Gene expression levels were calculated from non-log-transformed values derived from different platforms: linear-scale microarray intensity, DESeq2-normalized counts, or CPM, as detailed in the Methods. Key symbols and methodologies are as described in Fig. S5.

**Fig. S8 (Related to Fig. 4) Tissue-specific methylation of migration-linked genes between tissue and blood Tm populations.** (**a**) Heatmap of the tissue-specific DMRs in promoters and introns associated with chemokine receptor, S1P, and AHNAK in CD4 and CD8 CD69$^+$ Tm across tissues. Genes associated with DMRs discussed in this study are labeled according to their positions on the heatmaps. (**b-e**) Quantification of DMRs and reference tiles of indicated genes in CD69$^+$ (**b** and **d**) and CD69$^-$ (**c** and **e**) CD4 and CD8 Tm. Key symbols and methodologies are as described in Fig. S5.

**Fig. S9 (Related to Fig. 5; Table S7) Tissue-specific methylation of integrin genes between tissue and blood Tm populations.** (**a**) Heatmap of the tissue-specific DMRs in promoters and introns associated with integrin genes in CD69$^+$ CD4 and CD8 Tm across tissues. The genes of differential

methylation are highlighted for visual comparison. Genes associated with DMRs discussed in this study are labeled according to their positions on the heatmaps. (**b** and **c**) Quantification of DMRs and respective reference tiles of ITGAE (**b**) and ITGB2 (**c**) in tissue and blood CD69$^-$ CD4 and CD8 Tm. Key symbols and methodologies are as described in Fig. S5.

**Fig. S10 Tissue-specific methylation patterns of *RUNX3*.** (**a**) IGV visualization of the *RUNX3* gene locus, the key transcriptional regulator, showing tissue-specific hypo- and hyper-methylated DMRs for both CD4 and CD8 cells, including tissue CD69$^+$ Tm and blood samples. (**b**) Quantification of methylation difference for indicated DMRs shown in **a**. Key symbols and methodologies are as described in Fig. S5.

**Fig. S11 Tissue-specific methylation landscapes of *ZNF* family members in CD69$^+$ Tm of both CD4 and CD8 lineages.** (**a**) Heatmap of the top 10% tissue-specific DMRs in promoters and introns associated with the ZNF genes. Genes associated with DMRs discussed in this study are labeled according to their positions on the heatmaps. (**b**) IGV visualization of indicated tissue-specific ZNF hypo- and hyper-DMRs for both CD4 and CD8 T cells, including tissue CD69$^+$ and blood samples. The regions of differential methylation are highlighted for visual comparison. (**c**) Quantification of methylation differences for indicated DMRs or reference tiles. Key symbols and methodologies are as described in Fig. S5.

**Fig. S12 Dynamic tissue-specific methylation profiles of *KLF* family genes in CD69$^+$ Tm of both CD4 and CD8 lineages.** (**a**) Heatmaps of the tissue-specific DMRs in promoters and introns associated with KLF genes. Genes associated with DMRs discussed in this study are labeled according to their positions on the heatmaps. (**b-d**) IGV visualization (**b**) of tissue-specific KLF6 and KLF13 gene hypo- and hyper-DMRs and quantification of their DNA methylation levels for both CD69$^+$ (**c**) and CD69$^-$ (**d**) CD4 and CD8 Tm, including tissue and blood samples. The regions of differential methylation are highlighted for visual comparison. Multiple DMRs of the same gene may be present. Key to symbols as described in Fig. S5.

***Supplementary Tables***

**Table S1 (Related to Fig. 1a) Study subjects and corresponding methylome data**. Methylomes for samples labeled with ‘a’ or ‘b’ were generated in our previous work (Durek et al. 2016; Cendon et al. 2022) and have been deposited to the European Genome-Phenome Archive under accession numbers EGAS0000100624 (a) and EGAS00001005475 (b), respectively. Newly generated methylomes from the current study are deposited under accession number EGAS50000000085. Tm, memory T cells; Bm, bone marrow; Bl, blood; In, intestine; Sp, spleen; Sk, skin; Lu, lung; SP1, tissue CD69$^+$ Tm (i.e. conventional Trm); SP2, tissue CD69$^-$ Tm; Ct, blood (CD69$^-$) Tm. Sample labels follow the format Hfn (n), where H and f indicate ‘Human’ and ‘female’, and n denotes the sample number.

**Table S2 (Related to Fig. 1c and 1d) Genomic coordinates and annotation of all DMRs identified in this study**. This table lists the genomic coordinates and annotations of all DMRs identified, including associated gene features and locations. Annotations were performed as described in the methods section.

**Table S3 (Related to Fig. 2 and Fig. S5) Methylation values of identified DMRs associated with cytokine and chemokine genes.** This table presents DMRs associated with cytokine and chemokine genes for CD69$^+$ Tm across tissues and blood Tm. Each entry includes methylation values, genomic coordinates, and annotations of the DMRs. Genes were selected from all DMRs identified in this study, as shown in Table S2.

**Table S4 (Related to Fig. 3a,b, and Fig. S6) Top 3000 genes associated with DMRs in tissue CD69$^+$ and CD69$^-$ CD4 and CD8 Tm.** This table lists the top 3000 genes derived from the compiled tissue-specific promoter/intron DMRs across all comparisons of the four tissue-specific DMR set: CD69$^+$ and CD69$^-$ CD4 and CD8 Tm populations. Each datasheet includes methylation values, genomic coordinates, and annotations of the DMRs. Genes selection was performed as described in Fig. S5.

**Table S5 (Related to Fig. S7) Methylation values of DMRs associated with reported Trm core signature genes.** This table presents DMRs associated with the core signature genes for CD69$^+$ Tm across tissues and blood Tm. Each entry includes methylation values, genomic coordinates, and annotations of the DMRs. Genes were selected from all DMRs identified in this study, as shown in Table S2.

**Table S6 (Related to Fig. 4 and Fig. S10) Methylation values of DMRs associated migration-linked genes.** This table presents DMRs associated with migration-linked genes for CD69$^+$ Tm across tissues and blood Tm. Each entry includes methylation values, genomic coordinates, and annotations of the DMRs. Genes were selected from all DMRs identified in this study, as shown in Table S2.

**Table S7 (Related to Fig. 5 and Fig. S11) Methylation values of DMRs associated with integrin genes.** This table presents DMRs associated with integrin genes for CD69$^+$ Tm across tissues and blood Tm. Each entry includes methylation values, genomic coordinates, and annotations of the DMRs. Genes were selected from all DMRs identified in this study, as shown in Table S2.

**Table S8 (Related to Fig. S8) Methylation values of top 10% DMRs associated with *ZNF*s.** This table presents DMRs associated with *ZNF* genes for CD69$^+$ Tm across tissues and blood Tm. Each entry includes methylation values, genomic coordinates, and annotations of the DMRs. Genes were selected from all DMRs identified in this study, as shown in Table S2.

**Table S9 (Related to Fig. S9) Methylation values of DMRs associated with *KLF* family genes.** This table presents DMRs associated with *KLF* genes for CD69$^+$ Tm across tissues and blood Tm. Each entry includes methylation values, genomic coordinates, and annotations of the DMRs. Genes were selected from all DMRs identified in this study, as shown in Table S2.

**Table S10 Tissue-specific signature DMRs.** This table presents a summary of tissue-specific signature DMRs identified in this study.
