# Supplementary material for: Methylomes of human CD4 and CD8 memory T lymphocytes reveal tissue-specific epigenetic signatures for maintenance and recall function
Source: Immun Inflamm. 2025 Oct 1;1(1):13. doi: 10.1007/s44466-025-00009-x (PMC12623507; doi:10.1007/s44466-025-00009-x)
Supplement: Supplementary file 3 — Supplementary Material 3: Tables S1-S10. [file 44466_2025_9_MOESM3_ESM.zip › Suppl_Table_1. Study subjects and corresponding methylome data_R2.docx]

| Cell type (name data_file) | Donor pool or individual | # of mapped reads | Reference |
| --- | --- | --- | --- |
| CD4^+^CD69^+^ BM-Tm (51_Hf08_BmTM4_SP1) | Source: paired blood and bone marrow samples  Pool of 3  Mean age: 61y (75y, 52y, 57y)  Medical condition: Hip osteoarthritis (replacement surgery) | 80572605 | 1 |
| CD4^+^CD69^-^ BM-Tm (51_Hf08_BmTM4_SP2) |  | 99384971 | 1 |
| CD4^+^CD69^-^ Bl-Tm (51_Hf08_BlTM4_Ct) |  | 102528046 |  |
| CD8^+^CD69^+^ BM-Tm (51_Hf08_BmTM8_SP1) |  | 110329863 |  |
| CD8^+^CD69^-^ BM-Tm (51_Hf08_BmTM8_SP2) |  | 118345808 |  |
| CD8^+^CD69^-^ Bl-Tm (51_Hf08_BlTM8_Ct) |  | 105857267 |  |
| CD4^+^CD69^-^ Bl-Tm (51_Hf19_BlTM4_Ct) | Source: blood samples**;** Pool of 3; Mean age: 67y (69y. 74y, 59y)  Medical condition: Hip osteoarthritis (replacement surgery) | 31474956 |  |
| CD8^+^CD69^-^ Bl-Tm (51_Hf19_BlTM8_Ct) |  | 43838102 |  |
| CD4^+^CD69^+^ BM-Tm (51_Hf28_BmTM4_SP1) | Source: paired blood and bone marrow samples  Pool of 3 (n.a, 64y, 79y)  Mean age: 71y  Medical condition: Hip osteoarthritis (replacement surgery) | 38634958 | 2 |
| CD4^+^CD69^-^ BM-Tm (51_Hf28_BmTM4_SP2) |  | 51089044 |  |
| CD4^+^CD69^-^ Bl-Tm (51_Hf28_BlTM4_Ct) |  | 42018273 |  |
| CD8^+^CD69^+^ BM-Tm (51_Hf28_BmTM8_SP1) |  | 44210342 |  |
| CD8^+^CD69^-^ BM-Tm (51_Hf28_BmTM8_SP2) |  | 38023763 |  |
| CD8^+^CD69^-^ Bl-Tm (51_Hf28_BlTM8_Ct) |  | 54344089 |  |
| CD4^+^CD69^+^ In-Tm (51_Hf18_InTM4_SP1) | Source: paired blood and intestine samples  Pool of 3  Mean age: 41y (76y, 20y, 28y)  Medical condition: Colitis ulcerous (resection, healthy margin) | 49740872 |  |
| CD4^+^CD69^-^ In-Tm (51_Hf18_InTM4_SP2) |  | 25711399 |  |
| CD4^+^CD69^-^ Bl-Tm (51_Hf18_BlTM4_Ct) |  | 65370980 |  |
| CD8^+^CD69^+^ In-Tm (51_Hf18_InTM8_SP1) |  | 42836878 |  |
| CD8^+^CD69^-^ In-Tm (51_Hf18_InTM8_SP2) |  | 49093818 |  |
| CD8^+^CD69^-^ Bl-Tm (51_Hf18_BlTM8_Ct) |  | 40494355 |  |
| CD4^+^CD69^+^ In-Tm (51_Hf27_InTM4_SP1) | Source: intestine sample  Individual  Age: 33y  Medical condition: Colitis ulcerous (resection, healthy margin) | 71498085 |  |
| CD4^+^CD69^-^ In-Tm (51_Hf27_InTM4_SP2) |  | 83918906 |  |
| CD8^+^CD69^+^ In-Tm (51_Hf27_InTM8_SP1) |  | 106122673 |  |
| CD4^+^CD69^+^ Sp-Tm (51_Hf16_SpTM4_SP1) | Source: spleen samples  Pool of 3  Mean age: 43y (63y, 48y, 18y)  Medical condition: Diagnostic procedure (cryopreserved spleen mononuclear cells) | 66288243 | 2 |
| CD4^+^CD69^-^ Sp-Tm (51_Hf16_SpTM4_SP2) |  | 100838037 |  |
| CD8^+^CD69^+^ Sp-Tm (51_Hf16_SpTM8_SP1) |  | 122296114 |  |
| CD8^+^CD69^-^ Sp-Tm (51_Hf16_SpTM8_SP2) |  | 107018938 |  |
| CD4^+^CD69^+^ Sp-Tm (51_Hf17_SpTM4_SP1) | Source: spleen sample  Individual  Age: 49y  Medical condition: Diagnostic procedure (cryopreserved spleen mononuclear cells) | 95574894 | 2 |
| CD4^+^CD69^-^ Sp-Tm (51_Hf17_SpTM4_SP2) |  | 95915059 |  |
| CD8^+^CD69^+^ Sp-Tm (51_Hf17_SpTM8_SP1) |  | 107368853 |  |
| CD8^+^CD69^-^ Sp-Tm (51_Hf17_SpTM8_SP2) |  | 93573635 |  |
| CD4^+^CD69^+^ Sk-Tm (51_Hf14_SkTM4_SP1) | Source: paired blood and skin samples  Pool of 3 (33y, 28, 75y)  Mean age: 45y  Medical condition: Healthy donor (plastic surgery discard tissue) | 88662377 |  |
| CD4^+^CD69^-^ Sk-Tm (51_Hf14_SkTM4_SP2) |  | 82209786 |  |
| CD4^+^CD69^-^ Bl-Tm (51_Hf14_BlTM4_Ct) |  | 96502101 |  |
| CD8^+^CD69^+^ Sk-Tm (51_Hf14_SkTM8_SP1) |  | 65801825 |  |
| CD8^+^CD69^-^ Sk-Tm (51_Hf14_SkTM8_SP2) |  | 68293334 |  |
| CD8^+^CD69^-^ Bl-Tm (51_Hf14_BlTM8_Ct) |  | 100028060 |  |
| CD4^+^CD69^+^ Sk-Tm (51_Hf15_SkTM4_SP1) | Source: paired blood and skin samples  Individual  Age: 65y  Medical condition: Healthy donor (plastic surgery discard tissue) | 115026504 |  |
| CD4^+^CD69^-^ Sk-Tm (51_Hf15_SkTM4_SP2) |  | 86003720 |  |
| CD4^+^CD69^-^ Bl-Tm (51_Hf15_BlTM4_Ct) |  | 103433251 |  |
| CD8^+^CD69^+^ Sk-Tm (51_Hf15_SkTM8_SP1) |  | 45852890 |  |
| CD8^+^CD69^-^ Sk-Tm (51_Hf15_SkTM8_SP2) |  | 72798301 |  |
| CD8^+^CD69^-^ Bl-Tm (51_Hf15_BlTM8_Ct) |  | 89860497 |  |
| CD4^+^CD69^+^ Sk-Tm (51_Hf20_SkTM4_SP1) | Source: paired blood and skin samples  Pool of 3 (77y, 55, 53y)  Mean age: 62y  Medical condition: Healthy donor (plastic surgery discard tissue) | 36312095 |  |
| CD4^+^CD69^-^ Sk-Tm (51_Hf20_SkTM4_SP2) |  | 47172910 |  |
| CD4^+^CD69^-^ Bl-Tm (51_Hf20_BlTM4_Ct) |  | 43026783 |  |
| CD8^+^CD69^+^ Sk-Tm (51_Hf20_SkTM8_SP1) |  | 31920727 |  |
| CD8^+^CD69^-^ Bl-Tm (51_Hf20_BlTM8_Ct) |  | 39907103 |  |
| CD4^+^CD69^+^ Lu-Tm (51_Hf23_LuTM4_SP1) | Source: lung sample  Individual  Age: undefined adult  Medical condition: Adjacent healthy lung tissue (resected during surgery) | 44599323 |  |
| CD4^+^CD69^-^ Lu-Tm (51_Hf23_LuTM4_SP2) |  | 48609947 |  |
| CD8^+^CD69^+^ Lu-Tm (51_Hf23_LuTM8_SP1) |  | 46692532 |  |
| CD8^+^CD69^-^ Lu-Tm (51_Hf23_LuTM8_SP2) |  | 35049554 |  |
| CD4^+^CD69^+^ Lu-Tm (51_Hf24_LuTM4_SP1) | Source: lung sample  Individual  Age: undefined adult  Medical condition: Adjacent healthy lung tissue (resected during surgery) | 45357715 |  |
| CD4^+^CD69^-^ Lu-Tm (51_Hf24_LuTM4_SP2) |  | 34239220 |  |
| CD8^+^CD69^+^ Lu-Tm (51_Hf24_LuTM8_SP1) |  | 37523525 |  |
| CD8^+^CD69^-^ Lu-Tm (51_Hf24_LuTM8_SP2) |  | 41508991 |  |

**Table S1 Study subjects and corresponding methylome data**. Methylomes for samples labeled with ‘a’ or ‘b’ were generated in our previous work (Durek et al. 2016; Cendon et al. 2022) and have been deposited to the European Genome-Phenome Archive under accession numbers EGAS0000100624 (a) and EGAS00001005475 (b), respectively. Newly generated methylomes from the current study are deposited under accession number EGAS50000000085. Tm, memory T cells; Bm, bone marrow; Bl, blood; In, intestine; Sp, spleen; Sk, skin; Lu, lung; SP1, tissue CD69^+^ Tm (i.e. conventional Trm); SP2, tissue CD69^-^ Tm; Ct, blood (CD69^-^) Tm. Sample labels follow the format Hfn (n), where H and f indicate ‘Human’ and ‘female’, and n denotes the sample number.

**References:**

1. Durek, P., K. Nordstrom, G. Gasparoni, A. Salhab, C. Kressler, M. de Almeida, K. Bassler, T. Ulas, F. Schmidt, J. Xiong, P. Glazar, F. Klironomos, A. Sinha, S. Kinkley, X. Yang, L. Arrigoni, A. D. Amirabad, F. B. Ardakani, L. Feuerbach, O. Gorka, P. Ebert, F. Muller, N. Li, S. Frischbutter, S. Schlickeiser, C. Cendon, S. Frohler, B. Felder, N. Gasparoni, C. D. Imbusch, B. Hutter, G. Zipprich, Y. Tauchmann, S. Reinke, G. Wassilew, U. Hoffmann, A. S. Richter, L. Sieverling, Deep Consortium, H. D. Chang, U. Syrbe, U. Kalus, J. Eils, B. Brors, T. Manke, J. Ruland, T. Lengauer, N. Rajewsky, W. Chen, J. Dong, B. Sawitzki, H. R. Chung, P. Rosenstiel, M. H. Schulz, J. L. Schultze, A. Radbruch, J. Walter, A. Hamann, and J. K. Polansky. 2016. 'Epigenomic Profiling of Human CD4+ T Cells Supports a Linear Differentiation Model and Highlights Molecular Regulators of Memory Development', *Immunity*, 45: 1148-61.
2. Cendon, C., W. Du, P. Durek, Y. C. Liu, T. Alexander, L. Serene, X. Yang, G. Gasparoni, A. Salhab, K. Nordstrom, T. Lai, A. R. Schulz, A. Rao, G. A. Heinz, A. L. Stefanski, A. Claussnitzer, K. Siewert, T. Dorner, H. D. Chang, H. D. Volk, C. Romagnani, Z. Qin, S. Hardt, C. Perka, S. Reinke, J. Walter, M. F. Mashreghi, K. Thurley, A. Radbruch, and J. Dong. 2022. 'Resident memory CD4+ T lymphocytes mobilize from bone marrow to contribute to a systemic secondary immune reaction', *Eur J Immunol*.
